# Vascular adhesion protein-1 blockade in primary sclerosing cholangitis: Open-label, multicenter, single-arm, phase II trial

**DOI:** 10.1097/HC9.0000000000000426

**Published:** 2024-04-26

**Authors:** Gideon M. Hirschfield, Katherine Arndtz, Amanda Kirkham, Yung-Yi Chen, Richard Fox, Anna Rowe, Jessica Douglas-Pugh, Douglas Thorburn, Eleanor Barnes, Guruprasad P. Aithal, Diana Hull, Khushpreet Bhandal, Kathryn Olsen, Paul Woodward, Siân Lax, Philip Newsome, David J. Smith, Antero Kallio, David H. Adams, Victoria Homer, Chris J. Weston

**Affiliations:** 1 Institute of Immunology and Immunotherapy, University of Birmingham, Birmingham, UK; 2 National Institute for Health and Care Research (NIHR) Birmingham Biomedical Research Centre, Birmingham, UK; 3 Division of Gastroenterology and Hepatology, Toronto Centre for Liver Disease, University Health Network, Toronto, Ontario, Canada; 4 Cancer Research UK Clinical Trials Unit, Institute of Cancer and Genomic Sciences, University of Birmingham, Birmingham, UK; 5 Parexel International, Sheffield, UK; 6 Liver Services, Royal Free London NHS Foundation Trust, London, UK; 7 Nuffield Department of Medicine, University of Oxford, Oxford, UK; 8 Nottingham Digestive Diseases Centre, Translational Medical Sciences, School of Medicine, Faculty of Medicine and Health Sciences, The University of Nottingham, Nottingham, UK; 9 NIHR Nottingham Biomedical Research Centre, Nottingham University Hospitals and University of Nottingham, Nottingham, UK; 10 Biotie Therapies Corp., Turku, Finland

## Abstract

**Background:**

Primary sclerosing cholangitis is a progressive inflammatory liver disease characterized by biliary and liver fibrosis. Vascular adhesion protein-1 (VAP-1) is important in the inflammatory process driving liver fibrosis. We evaluated the safety and efficacy of VAP-1 blockade with a monoclonal antibody (timolumab, BTT1023) in patients with primary sclerosing cholangitis.

**Methods:**

BUTEO was a prospective, single-arm, open-label, multicenter, phase II trial, conducted in 6 centers in the United Kingdom. Patients with primary sclerosing cholangitis aged 18–75 years had an alkaline phosphatase value of >1.5 times the upper limit of normal. The dose-confirmatory stage aimed to confirm the safety of timolumab through the incidence of dose-limiting toxicity and sufficient trough levels of circulating antibody to block VAP-1 function. The primary outcome of the dose-expansion portion of the trial was patient’s response to timolumab at day 99, as measured by a reduction in serum alkaline phosphatase by 25% or more from baseline to day 99.

**Results:**

Twenty-three patients were recruited: 7 into the initial dose-confirmatory stage and a further 16 into an expansion stage. Timolumab (8 mg/kg) was confirmed to be safe for the duration of administration with sufficient circulating levels. Only 2 of the 18 evaluable patients (11.1%) achieved a reduction in alkaline phosphatase levels of 25% or more, and both the proportion of circulating inflammatory cell populations and biomarkers of fibrosis remained unchanged from baseline.

**Conclusions:**

The BUTEO trial confirmed 8 mg/kg timolumab had no short-term safety signals and resulted in sufficient circulating levels of VAP-1 blocking timolumab. However, the trial was stopped after an interim assessment due to a lack of efficacy as determined by no significant change in serum liver tests.

## INTRODUCTION

Primary sclerosing cholangitis (PSC) is a progressive inflammatory liver disease characterized by progressive biliary fibrosis, seen across all ages, and frequently in association with inflammatory bowel disease (IBD). PSC has an incidence of between 0.5 and 1.0/100,000 annually, and a prevalence of at least 16.2 per 100,000.^1–5^ More than 50% of patients require liver transplantation within 10–15 years of symptomatic presentation,^6,7^ reflecting the failure of medical therapies to sufficiently impact clinical outcomes. Progression to end-stage cirrhosis and/or hepatobiliary malignancies is considered to be driven by a chronic inflammatory response and immune cell–mediated injury to medium-large bile ducts.^8^

Studies have demonstrated that the 170-kDa homodimeric transmembrane sialoglycoprotein, vascular adhesion protein-1 (VAP-1), is constitutively expressed on human hepatic endothelium, and plays an important role in the inflammatory process that drives fibrogenesis in liver disease.^9^ VAP-1 functions as an adhesion molecule mediating the transmigration of leukocytes from the blood through the endothelial lining into surrounding tissues.^10^ VAP-1 is also a copper-dependent semicarbazide-sensitive amine oxidase which catalyzes the oxidative deamination of exogenous and endogenous primary amines resulting in the generation of aldehyde, ammonia, and hydrogen peroxide. These products activate NFκB-dependent chemokine secretion and adhesion molecule expression in liver endothelium.

Concentrations of VAP-1 in hepatic tissue, and soluble (s)VAP-1 in serum, are elevated in chronic liver diseases and correlate with histological fibrosis.^11,12^ Moreover, in vivo data demonstrate that blocking VAP-1 function with an anti-mouse VAP-1 antibody significantly alleviates inflammation in mouse models of arthritis and lung inflammation. Treatment with an antibody against VAP-1 also prevents hepatic fibrosis in murine models of liver injury, with loss of VAP-1 enzyme activity or antibody blockade leading to a reduction in the recruitment of immune cells into inflamed tissue.^13^

Timolumab (BTT1023) is a fully human, monoclonal, anti-VAP-1 antibody, which blocks the adhesion function of VAP-1 thereby diminishing leukocyte entry into sites of tissue inflammation, without affecting its semicarbazide-sensitive amine oxidase activity toward small molecular substrates (Biotie Therapies, unpublished data). Timolumab appears to be safe and well-tolerated in humans having been given in doses up to 8 mg/kg in patients with rheumatoid arthritis and psoriasis after oral premedication (cetirizine and ibuprofen). In addition, timolumab appears to be safe and well-tolerated when administered through repeated i.v. dosing at 2 weekly intervals, with no cytokine release syndrome experienced by patients. With no current medical therapy shown to be effective in altering the progression of PSC, the aim of the BUTEO trial (a single-arm, two-stage, multicenter, phase II clinical trial investigating the safety and activity of the use of BTT1023, a human monoclonal antibody targeting vascular adhesion protein [VAP-1], in the treatment of patients with PSC) was to evaluate the short-term biochemical safety and efficacy of VAP-1 blockade by timolumab in patients with PSC.

## METHODS

### Study design

The BUTEO trial was a single-arm, open-label, two-stage phase II clinical trial recruiting patients from 6 hospitals in the United Kingdom. The study protocol (ultimately Version 5.0 dated July 31, 2018) was conducted in accordance with both the Declarations of Helsinki and Istanbul as reflected in a priori approval by the National Research Ethics Committee East Midlands—Derby (Ref: 14/EM/1272) and local institutional review boards and ethical committees. Written consent was given in writing by all subjects. The BUTEO trial protocol has already been published^14^ and is included in Supplemental Appendix 1, http://links.lww.com/HC9/A865. Therefore, only key and/or updated information has been included.

BUTEO was prospectively registered at EudraCT (Number: 2014-002393-37); ISRCTN (Number: 11233255); Clinicaltrials.gov (Identifier: NCT02239211).

### Study drug

Timolumab (BTT1023) is a fully human, anti-VAP-1, modified IgG4 monoclonal antibody developed by Biotie Therapies Ltd and manufactured under Good Manufacturing Practice by Rentschler Biopharma SE. The investigational drug was supplied to the study consortium by Biotie Therapies.

### Patients

Clinician investigators identified eligible patients from ambulatory hepatology clinics. Participants were aged 18–75 years and had a clinical diagnosis of PSC, as evidenced by chronic cholestasis of more than 6 months duration with either an MRI or liver biopsy consistent with PSC and in the absence of a documented alternative etiology. Alkaline phosphatase (ALP) values were required to be at least 1.5 times the upper limit of normal (Clinical Laboratory Services, Queen Elizabeth Hospital, Birmingham, UK).

Pregnant and breast-feeding women were excluded and those with reproductive potential were required to use effective methods of contraception, defined as at least one the following methods for heterosexual intercourse: combined (estrogen and progestogen containing) hormonal contraception associated with inhibition of ovulation; progestogen-only hormonal contraception associated with inhibition of ovulation; intrauterine device; intrauterine hormone-releasing system; bilateral tubal occlusion; vasectomized partner; and/or sexual abstinence. Female participants must also undergo pregnancy tests before treatment.

All patients gave written informed consent. Patient registration into the trial by the treating clinician was by telephone to the central registration service at the Cancer Research UK Clinical Trials Unit (CRCTU) at the University of Birmingham for the dose-confirmation phase and through an electronic remote data capture system for the dose-expansion phase.

### Interventions and procedures

Patients attended clinical research sites and were cared for by registered nurses. Following premedication with cetirizine 10 mg and ibuprofen 400 mg orally (in the absence of any contraindications) plus i.v. hydrocortisone 100 mg, 1–2 hours preinfusion, all registered patients received timolumab through i.v. infusion at 8 mg/kg body weight. Up to a maximum of 7 doses were given during outpatient hospital visits; the first dose was infused over 120 minutes, with subsequent doses infused over 60 minutes. Doses were given on days 1, 8, 22, 36, 50, 64, and 78 (±3 d). Follow-up data were collected on days 99 and 120 (±3 d) after treatment initiation. All patients received 1-dose level and no dose reductions were permitted.

Pretreatment parameters recorded included Mayo PSC Risk Score (the established risk score at the time of BUTEO’s inception^15^), Model for End-Stage Liver Disease score,^16^ and a Fibroscan, which were repeated at day 99. In addition, a LiverMultiscan MRI was performed^17^ where possible. This was repeated on day 120.

All adverse events according to NCI-Common Terminology Criteria for Adverse Events (CTCAE) v4.0^18^ were recorded.

Quality of life questionnaires (EQ-5D 5L,^19^ Fatigue Severity Scale,^20^ pruritus visual analog score^21^) were administered by research nurses, preinfusion of the first and fourth doses of treatment and on day 99 of follow-up. If a patient had IBD, an IBD diary was given to them within the screening visit, to be completed before their first and fourth treatment visits as well as before the day 99 follow-up visit. All questionnaires and diaries were completed independently by patients.

### Trial outcomes

For the dose-confirmatory portion of the trial, outcomes were incidence of dose-limiting toxicities (DLTs), where the acceptable level was 1 in 6 patients (~17%) in line with a 3+3 design, and timolumab activity level, as ascertained through trough levels of timolumab. The stipulated acceptable trough levels of timolumab were set at 3 µg/mL free circulating timolumab at 8 weeks from the first infusion, which is ~100-fold the dissociation constant (Kd) of timolumab from VAP-1 resulting in target occupancy of ~90%.

A DLT was defined as an adverse event that meets the criteria of grade 3 cytokine release syndrome or grade 4 or 5 for any criteria, as defined in the NCI-CTCAE v4.0,^18^ and considered to be at least possibly related to timolumab treatment. The DLT reporting period was defined as the treatment period from the first treatment dose (day 1) to day 99 after treatment.

The primary outcome measure was the patient’s response to treatment at day 99, as measured by a reduction in serum ALP levels by 25% or more from baseline to day 99. If a patient’s day 99 ALP sample is missing, they are treated as a nonresponder. Serum ALP was analyzed at a central laboratory in Birmingham, UK to reduce between-laboratory analytical variability and ensure all samples were analyzed using the same equipment and procedures.

Secondary outcome measures of safety and tolerability are detailed in Supplemental Appendix 2, http://links.lww.com/HC9/A865. Additional, nonprotocol-defined exploratory outcomes included measuring circulating markers of inflammatory cells, extracellular matrix degradation, fibrotic tissue remodeling, and fibrogenesis/fibrolysis.

### Biological procedures

#### Measurement of circulating timolumab, sVAP-1, circulating markers of fibrosis, and amine oxidase activity

Circulating concentrations of timolumab and sVAP-1 were measured for visits 3 to 11 by Envigo. Novel circulating markers of fibrosis development and remodeling were analyzed by Nordic Bioscience using validated competitive ELISAs. See Supplemental Appendix 2, http://links.lww.com/HC9/A865 for details. Serum amine oxidase activity was measured using an Amplex UltraRed-based assay to detect the evolution of hydrogen peroxide as described in Supplemental Appendix 2, http://links.lww.com/HC9/A865.

#### Determination of circulating immune cell populations by flow cytometry

Peripheral blood mononuclear cells were isolated with inflammatory cell populations and were analyzed by flow cytometry as described in Supplemental Appendix 2, http://links.lww.com/HC9/A865.

### Statistical analysis

#### Phase I: Dose confirmation

The BUTEO trial incorporated a conventional 3+3 cohort design to confirm the therapeutic dose of timolumab, with decisions regarding continuation based on toxicity and pharmacokinetic data. The first 6 patients registered received the starting dose (8 mg/kg). Recruitment was then paused to await the results of trough blood serum levels of circulating timolumab from all patients after receiving their seventh dose (day 50) and until the DLT reporting period (day 99).

#### Phase II: Simon’s 2-stage minimax design

Once a dose had been confirmed, the trial’s design permitted expansion up to a total of 37 patients receiving the confirmed dose of timolumab. Statistical analyses were carried out on a modified intention-to-treat basis in which only patients who have received at least 1 infusion at the confirmed dose of timolumab were analyzed. Those patients not receiving the confirmed dose were not included in the final analyses.

The sample size for the phase II expansion was calculated based on a single-arm Simon’s 2-stage minimax design^22^ with lower and upper acceptability bounds of 15% and 30%, respectively, and error rates of α=0.1 and β=0.2 and inflated for ~10% patient drop out. A response was a patient experiencing a reduction in cholestasis as indicated by a reduced serum ALP by 25% or more when comparing baseline to day 99.

Additional statistical considerations are described in Supplemental Appendix 2, http://links.lww.com/HC9/A865 with the trial Statistical Analysis Plan included in Supplemental Appendix 3, http://links.lww.com/HC9/A865.

Descriptive statistics are presented as mean and SD or median, IQR, and minimum and maximum range for numerical variables (dependent on distribution), with frequency and percentage given for categorical variables.

Analyses were carried out using R, version 3.6.0.

#### Biological procedures

Data were plotted and analyzed using GraphPad Prism (v9.2.0). Shapiro-Wilk analysis was used to test for normal distribution and data were subsequently analyzed by Kruskal-Wallis test with Dunn multiple comparison test.

### Role of the funding source

Funding came from the Efficacy and Mechanism Evaluation (EME) Programme, a Medical Research Council (MRC) and National Institute for Health and Care Research (NIHR) partnership. The trial was initiated and conducted independently by the trial investigators. The funder had no role in trial design, data collection, data analysis, data interpretation, or writing of the report. The corresponding author had full access to all the data in the trial and had final responsibility for the decision to submit for publication.

## RESULTS

### Participants

**FIGURE 1:**
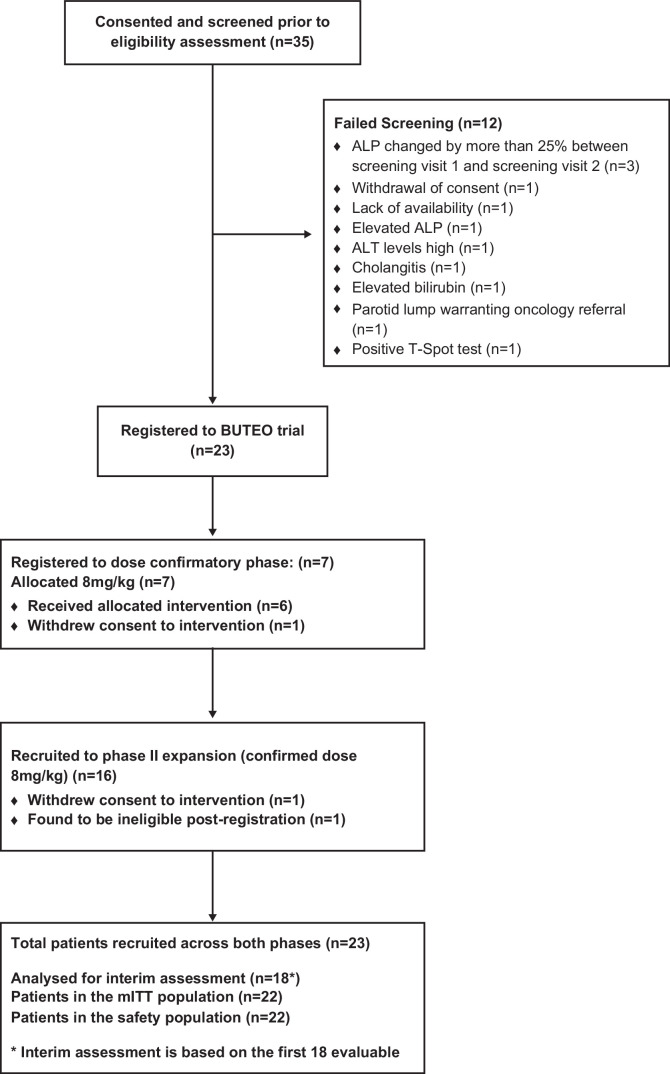
BUTEO trial profile. CONSORT diagram of the BUTEO trial. One patient was registered and then found to be ineligible; they are therefore counted as both a screen failure and as a patient recruited. Abbreviations: ALP, alkaline phosphatase; mITT, modified intention-to-treat.

Between April 14, 2015, and June 19, 2018, 35 patients were screened for the BUTEO trial with a total of 23 patients registered (Figure 1). Seven patients were recruited into the phase I, dose confirmation stage; 1 patient withdrew after 1 dose of timolumab and was replaced. Sixteen patients were recruited into the first stage of the phase II dose expansion of which 1 patient was found to be ineligible after registration and was excluded from all analyses, with a second withdrawing consent having received 5 doses of timolumab who, as they had received at least 1 timolumab infusion, were evaluable for analysis and provided both samples required for the primary analysis (Supplemental Appendix 4, http://links.lww.com/HC9/A865).

Baseline patient characteristics are described in Table 1. The median age for patients in the trial was 45.3 years (range: 22–69), with 19 (83%) male, a mean age of PSC onset of 36.6 years (range: 17–65), and 14 (61%) with a history of IBD.

**TABLE 1:** Patient characteristics

Patient baseline characteristics, n (%)	N=23
Age (y)	
Mean (SD)	45.3 (13)
Range	22–69
Sex	
Male	19 (83)
Female	4 (17)
Ethnicity	
White	20 (87)
South Asian	2 (9)
Unrecorded	1 (4)
ALP values at the screening visit (IU/L)^a^	
Median (IQR)	450.77 (311, 566)
Range	215–1075
Age at PSC onset (y)	
Mean (SD)	36.6 (13)
Range	17–65
Diagnosis of disease	
Established	12 (52)
New	8 (35)
Not known	3 (13)
UDCA at screening	
No	11 (48)
Yes	12 (52)
Disease diagnosis	
Small duct PSC	6 (26)
Large duct PSC	17 (74)
Fibroscan in the cirrhotic range^b^	
No	11 (48)
Yes	11 (48)
Unknown	1 (4)
History of inflammatory bowel disease	
No	8 (35)
Yes	14 (61)
Not known	1 (4)

a ALP at screening was unreported for 1 patient; thus, based on 22 patients.

b As defined as a Fibroscan result of >14.4 kPa.^23^

Abbreviations: ALP, alkaline phosphatase; PSC, primary sclerosing cholangitis; UDCA, ursodeoxycholic acid.

### Dose confirmation

Six patients evaluated in the dose confirmation phase received the 7 scheduled 8 mg/kg dose of timolumab. No DLTs were observed in any of the patients and the circulating concentrations of timolumab at 8 weeks after the first infusion exceeded the minimum requirement of 3 μg/mL for all patients (Supplemental Appendix 5, http://links.lww.com/HC9/A865). Therefore, the dose of timolumab was confirmed and deemed safe at 8 mg/kg, and the trial expanded into the phase II stage.

A further 12 patients were recruited into the dose-expansion phase to determine whether there was any evidence of treatment efficacy; 1 patient missed 1 timolumab dose, and 1 withdrew and discontinued treatment after 5 doses (Supplemental Appendix 4, http://links.lww.com/HC9/A865).

#### No change in ALP was observed over the duration of the study

**FIGURE 2:**
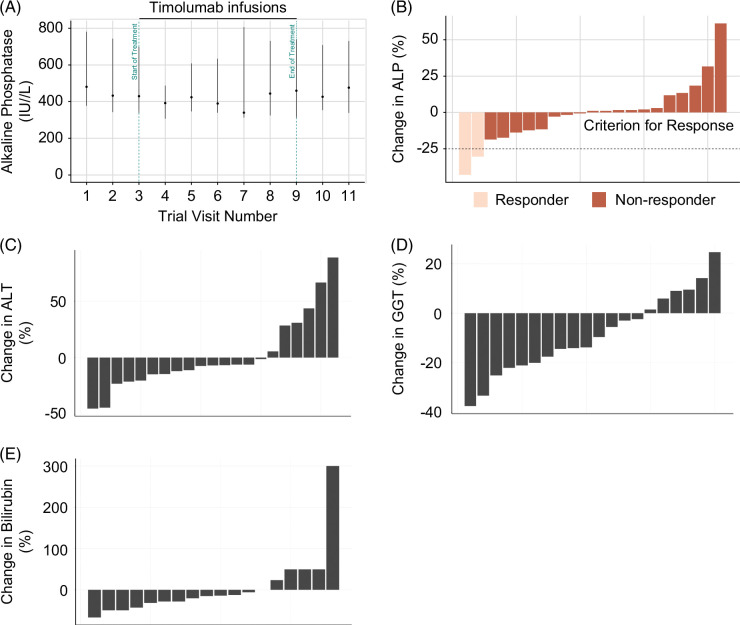
Liver biochemistry pre-timolumab, during and post-timolumab treatment. (A) Median with IQRs of ALP (IU/L) in all evaluable patients during the BUTEO trial. Percentage change of ALP (B), ALT (C), GGT (D), and bilirubin (E) levels comparing pretreatment and posttreatment in all evaluable patients. Abbreviations: ALP, alkaline phosphatase; GGT, gamma-glutamyl transferase.

During the interim assessment, 9 of the 18 patients (50.0%) recruited experienced a reduction in ALP levels, with 8/18 (44.4%) experiencing an increase (Figures 2A, B). One patient was missing their day-99 follow-up data but was still included in the interim analysis data set. Only 2 of the 18 evaluable patients (11.1%) achieved a reduction in ALP of 25% or more, meaning that the BUTEO trial failed to reach the minimum requirement to continue recruitment. The trial was, therefore, stopped early due to a lack of efficacy.

Four patients were recruited into the trial during the interim assessment (Supplemental Appendix 4, http://links.lww.com/HC9/A865); therefore, data collected from all patients during the trial were analyzed together, resulting in a total sample size of 22. None of these additional patients achieved a reduction in ALP of ≥25% resulting in a total of 2/22 patients (9.1%) who achieved a response (Supplemental Appendix 6, http://links.lww.com/HC9/A865).

### Secondary outcomes

#### Serum liver tests and liver inflammation and fibrosis Multiscan scores remained unchanged using a safely identified dose of timolumab

Liver biochemistry remained unchanged when comparing pre-timolumab and post-timolumab treatment (Figures 2B–D, Supplemental Appendix 6, http://links.lww.com/HC9/A865, and Supplemental Table S6A, http://links.lww.com/HC9/A865), as did liver inflammation and fibrosis Multiscan scores (Supplemental Appendix 6, http://links.lww.com/HC9/A865 and Supplemental Table S6B, http://links.lww.com/HC9/A865). In addition, no deaths or DLTs were reported during the trial. A total of 1133 adverse events were reported, affecting all 22 registered patients (Table 2). There were 4 severe adverse events affecting 4 patients (18.2%); these included 2 suspected unexpected serious adverse reactions that were due to a hypersensitivity response and requirement for a transplant earlier than anticipated pre-trial.

**TABLE 2:** Summary of safety data

Safety data	N=22 (%)
Serious adverse events	
Incidence	4
Patients affected	4 (18.2)
Category	
Unrelated SAE	2
SAR	0
Nonfatal/life-threatening SUSAR	2
Fatal/life-threatening SUSAR	0
Reason	
Death	0
Life-threatening	0
Hospitalization	2
Disability	0
Congenital anomaly	0
Other	2
Event	
Infusion-related reaction	1
Diarrhea	1
Blood bilirubin increased	1
Colitis	1
Grade	
1	0
2	0
3	3
4	1
5	0
Dose-limiting toxicity	
Incidence	0
Patients affected	0
Adverse events	
Incidence	1133
Patients affected	22 (100.0)
Grade	
1	828
2	221
3	74
4	11
5	0
Relatedness	
Unrelated	864
Unlikely to be related	126
Possibly related	108
Probably related	24
Definitely related	12

Abbreviations: SAR, serious adverse reaction; SUSAR, suspected unexpected serious adverse reaction.

#### Unchanged quality of life and acceptable adherence to timolumab

No apparent changes were observed in disease-related symptoms when questionnaire scores were compared pre- and post-timolumab treatment (Table 3). Nineteen patients (83.4%) were fully adherent with treatment visits (Supplemental Appendix 4, http://links.lww.com/HC9/A865). The reasons for nonadherence included withdrawal from the study (2 patients) and 1 missed treatment cycle for unknown reasons (1 patient).

**TABLE 3:** Quality of life scores

Questionnaire	Pretreatment visit 3	Posttreatment visit 10	Difference
EQ-5D 5L			
Median (IQR)	1 (0.88, 1)	1 (0.85, 1)	0 (−0.084, 0)
Range	(0.77, 1)	(0.23, 1)	(−0.48, 0.17)
EQ-VAS			
Median (IQR)	83 (75, 95)	82.5 (64.5, 90)	0 (−10, 5)
Range	(75, 95)	(64.5, 90)	(−10, 5)
FSS			
Median (IQR)	34 (24, 46)	38 (20.25, 49.25)	−1 (−4, 3)
Range	(24, 46)	(9, 58)	(−10, 10)
Pruritus VAS			
Median (IQR)	29 (12, 38)	24 (13, 30)	−9 (−14.25, 4.25)
Range	(2, 92)	(1, 78)	(−53, 51)

Abbreviations: EQ-5D 5L, EuroQol-5 Dimension, 5 Levels; EQ-VAS, EuroQol visual analog scale; FSS, fatigue severity scale.

#### Timolumab reduced serum sVAP concentrations

**FIGURE 3:**
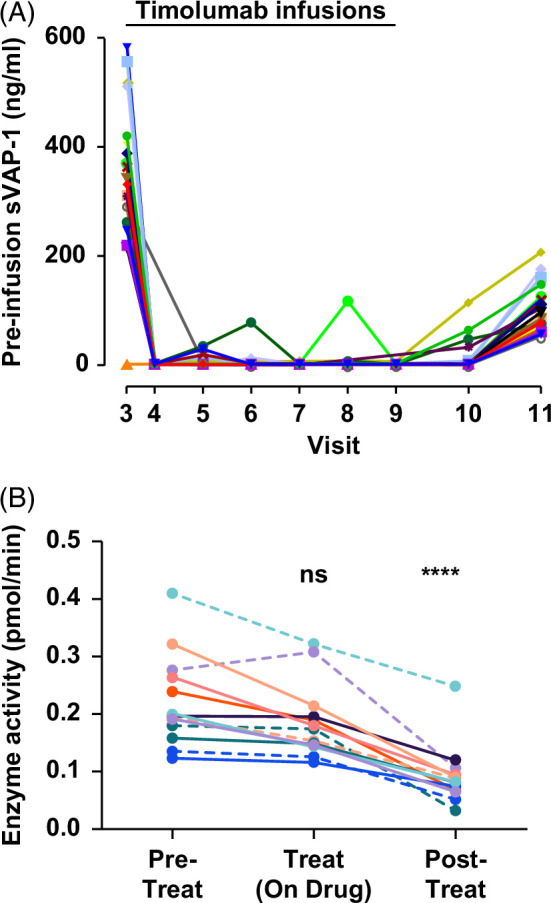
Concentration and enzymatic activity of circulating sVAP-1. (A) Concentration of sVAP-1 in patient serum was measured before each infusion of timolumab and during the posttreatment phase (n=21). (B) Amine oxidase activity of patient serum was determined by Amplex UltraRed assay. Data were background-corrected using the VAP-1 inhibitor LJP1586 (1 μM). Data for 13 patients shown with enzyme activity determined at each visit and then averages taken for pretreatment, treatment, and posttreatment (follow-up visits 10–11: days 99 and 120). Mean±IQR are shown. Kruskal-Wallis test with Dunn multiple comparison test vs. pretreatment group (ns, not significant, ****p<0.001). Abbreviation: sVAP-1, soluble vascular adhesion protein-1.

Administration of timolumab led to a significant reduction in sVAP-1 protein concentration, suggesting successful target engagement of the antibody (*p*<0.0001, Figure 3A). This was maintained until the final timolumab dose (visit 9) after which the concentration began to rise in all patients. Furthermore, the enzymatic activity of circulating VAP-1 (measured by amine oxidase activity toward the primary amine benzylamine) was significantly reduced (*p*<0.001) in patients having received all scheduled timolumab treatments (Figure 3B).

#### Variable numbers of circulating inflammatory cell subtypes were observed

**FIGURE 4:**
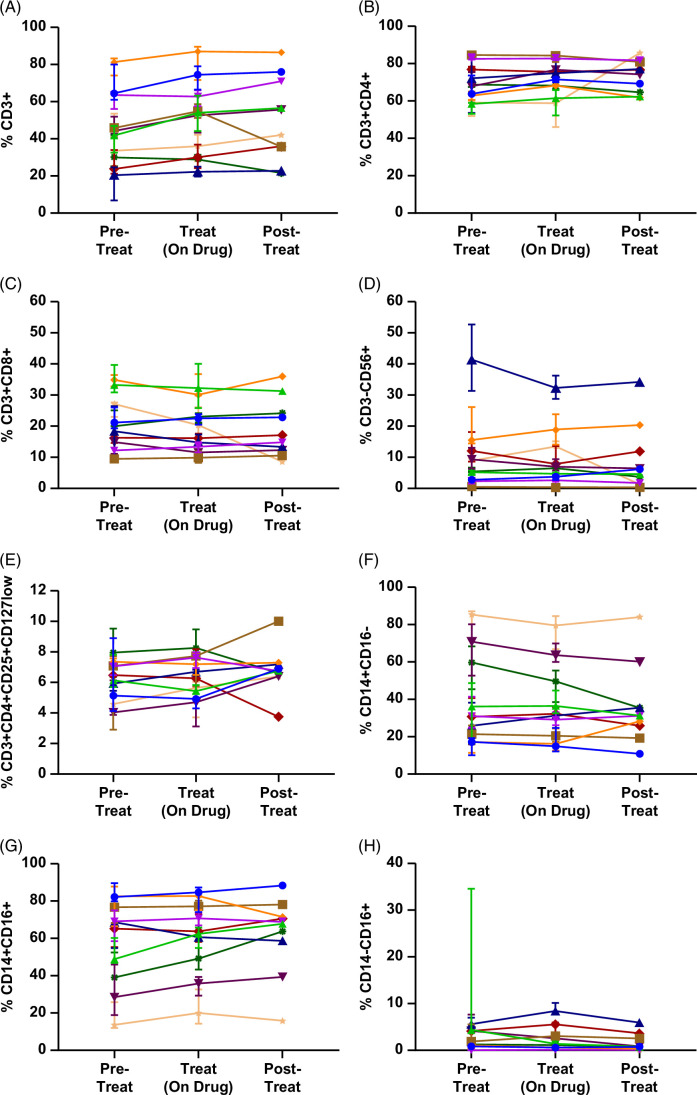
Proportions of circulating inflammatory cell populations that have been shown to be recruited into vascular beds by VAP-1. Cryopreserved peripheral blood mononuclear cells were thawed and immunophenotyped using flow cytometry for (A) CD3+, (B) CD4+, and (C) CD8+ T cells, (D) CD3-CD56+ NK cells, (E) regulatory T cells (Treg), and (F) classical (CD14+CD16−), (G) intermediate (CD14+CD16+), and (H) nonclassical (CD14−CD16+) monocyte populations. Aggregated data collected during pretreatment (visits 1–3: screening visits 1 and 2, pre-first infusion visit 3 [day 1]), treatment (infusion visits 4–9: days 8, 22, 36, 50, 64, and 78), and posttreatment (follow-up visits 10–11: days 99 and 120) groups. There were no significant differences between groups (Kruskal-Wallis test with Dunn multiple comparison test). Median±IQR. Abbreviation: VAP-1, vascular adhesion protein-1.

Despite reductions in sVAP-1 concentration and enzyme activity, circulating proportions of inflammatory cell populations commonly associated with VAP-1 activity remained unchanged from baseline during and following treatment; proportions were variable in patients, pre-timolumab treatment (mean pretreatment/on-treatment/posttreatment values for CD3+ cells 45.4/49.1/50.4%, for CD3+CD4+ cells 69.7/72.0/73.5%, for CD3+CD8+ cells 20.8/19.6/19.1%, for NK cells 10.8/10.0/9.1%, for Treg 6.2/6.4/6.8%, for classical monocytes 38.9/36.9/36.2%, for intermediate monocytes 57.8/60.9/62.3%, and for nonclassical monocytes 3.7/2.3/1.6%) (Figure 4). Similar results were also seen for other inflammatory cell subtypes that may contribute to PSC pathology (Supplemental Appendix 7, http://links.lww.com/HC9/A865).

#### No change in markers of liver fibrosis/stiffness was observed

No clinically meaningful changes were observed in liver fibrosis when measured by the enhanced liver fibrosis score (median [range]: pretreatment 10.2 [8.6–17.3], posttreatment 10.4 [8.6–12.7]) or Fibroscan (median difference: 0.31, IQR: −0.04 to 0.75) pre- and post-timolumab treatment (Table 4 and Supplemental Appendix 8, http://links.lww.com/HC9/A865 and Supplemental Figures S8A, B, http://links.lww.com/HC9/A865). In addition, using a more sensitive approach to determine the impact of timolumab administration on fibrogenesis and fibrolysis, no significant differences were observed between the concentration of serum extracellular matrix turnover biomarkers measured at baseline and those taken during and/or following treatment (Supplemental Appendix 8, http://links.lww.com/HC9/A865, and Supplemental Figures S8C, D, http://links.lww.com/HC9/A865). Baseline concentrations of fibrogenesis and fibrolysis markers, and enhanced liver fibrosis score reported during the BUTEO trial were comparable to those reported previously (Table 4).

**TABLE 4:** Concentrations of markers of fibrogenesis and fibrolysis at baseline compared to selected published liver injury data and healthy controls

	BUTEO trial (this study)	Nonadvanced PSC	Advanced PSC	Healthy controls	Advanced NASH
		Vesterhus et al^24^		Kehlet et al^25^	Lou et al^26^
Collagen synthesis					
ProC3					
Median	19.5	13.3	42.2	7.9	24.6 (F3 fibrosis) 23.5 (F4 fibrosis)
Range	8.6–60.5	6.8–84.7	11.7–104	4.0–27.3	NA
ProC5					
Median	344.6	808.0	795.9	NA	345.1 (F3 fibrosis) 262.5 (F4 fibrosis)
Range	0–1289	359.5–2990.2	56.1–3035.2	NA	NA
Collagen degradation					
C3M					
Median	15.5	10.7	12.8	9.7	8.4 (F3 fibrosis) 8.2 (F4 fibrosis)
Range	8.4–22.6	5.6–31.4	7.2–48.9	5.0–22.7	NA
C4M2					
Median	27.2	27.2	29.4	18	22.6 (F3 fibrosis) 22.0 (F4 fibrosis)
Range	8.9–51.2	15.0–73.4	14.6–65.7	9.3–59.4	NA
P4NP7S					
Median	229.0	NA	NA	203.4	151.2 (F3 fibrosis) 137.4 (F4 fibrosis)
Range	52.3–548.8	NA	NA	89.7–593.9	NA
ECM degradation/synthesis					
C3M/ProC3					
Ratio	0.7	0.7	0.3	NA	NA
ELF^a^					
	PRE	Test cohort			
Median	10.2	10.1		NA	NA
Range	8.6–12.3	7.3–14.3			
	POST	Validation cohort			
Median	10.4	9.7		NA	NA
Range	8.6–12.7	7.1–15.7			

*Note*: Data for ProC3, ProC5, C3M, C4M2, and P4NP7S are presented as ng/mL.

a Reference ELF data are taken from Day et al^27^: <7.7 excludes significant fibrosis; ≥7.7 and <9.8 moderate fibrosis; ≥9.8 and <11.3 severe fibrosis; ≥11.3 cirrhosis.

Abbreviations: ECM, extracellular matrix; ELF, enhanced liver fibrosis; NA, not available; POST, post-last infusion of timolumab (BTT1023), Visit 10 in the BUTEO trial; PRE, prior to infusion of timolumab (BTT1023), Visit 3 in the BUTEO trial; PSC, primary sclerosing cholangitis.

## DISCUSSION

Treatment trials in PSC remain challenging, based on a limited understanding of disease pathophysiology, coupled with a heterogeneity of patient outcomes. Furthermore, the disease remains rare, and validated surrogate biomarkers of outcome are lacking. We sought to evaluate the biochemical safety and efficacy of VAP-1 blockade by timolumab in patients with PSC, premised on preclinical data supporting the role of VAP-1 in pathophysiology. The trial concluded that an 8 mg/kg dose of timolumab was safe over the short-term use studied in all patients treated and resulted in biologically relevant circulating concentrations of timolumab above 3 µg/mL. However, the trial was stopped early after an interim assessment on the grounds of futility and a lack of biochemical efficacy as evaluated by serum liver tests.

The heterogeneity of PSC along with its rare orphan disease status poses challenges in trial design. ALP values are prognostic and identify patients at risk of disease progression. However, while ALP has traditionally been used in early-phase PSC trials as an efficacy marker (historically because therapies focused on the biliary tree), ALP values fluctuate during the natural course of the disease, which limits its usefulness as a primary endpoint. While we adopted a traditional study design looking at ALP values, we added exploratory markers of liver fibrosis, recognizing that in a pilot safety and dose-finding study this would remain a limitation to drug evaluation. While a longer placebo-controlled study would have been desirable, funding was not forthcoming for such a design.

While our study did not confirm the benefit, the results are nevertheless important for several reasons. The trial design incorporated a dose-confirmatory and safety stage (based on the traditional 3+3 design), then followed by a phase II Simon’s 2-stage design. It aligned routine trial conduct with an effort to translate laboratory research into a proof-of-concept clinical trial. Accepting the limitations of standard serum liver tests, it also included emerging biomarkers to assess extracellular matrix synthesis and turnover.

While our study demonstrated no apparent safety concerns for VAP-1 blockade, equally we did not see immediate efficacy; a residual challenge in developing any therapy for PSC is to understand when in the disease course utility is predicted to be greatest. In that regard, one consideration for future studies of this, and similar agents, is designing trials, with appropriate biomarkers, that can evaluate efficacy in earlier-stage patients.

The limitations are clear and include a small cohort of patients with established diseases studied over a short time frame. Clinical benefits on liver fibrosis may require more sustained treatment; the optimal duration for a trial testing impact on liver fibrosis in PSC is not clearly known. Clinical trials are inherently expensive, and discourse is needed to decide if fewer longer/larger studies might better serve patients. PSC can have a natural history of over 20 years and hence a 3-month study is a small disease snapshot. The use of a placebo would have been clearly advantageous but required additional funding that was not available; equally at the outset, the study had a dose-finding component to it. As is evident, all the markers measured were variable in nature when measured and our data highlight a key rate-limiting step for future PSC trials, the ongoing absence of stable, robust, and clinically confirmed surrogates of disease.

We hypothesized that timolumab may block inflammatory cell migration, leading to an increased prevalence of certain immune cell populations in the circulation. There were no indicators that VAP-1 blockade was having either a beneficial or detrimental impact on the patient’s immune response. Previous animal models suggested a role for VAP-1 in the progression of hepatic fibrosis. Circulating biomarkers of fibrosis (including enhanced liver fibrosis) and Fibroscan data were unchanged both during and after administration of timolumab. Furthermore, it is possible that efficacy requires blockade of the enzymatic function of VAP-1 which antibody treatment does not deliver. We were unable to obtain longitudinal liver biopsy material from these patients, preventing us from evaluating any changes in the intrahepatic immune cell populations or pathological fibrosis scores arising from timolumab administration. That said if liver biopsy was considered important for a study such as this, it would have necessitated a much longer study duration/treatment exposure. Of note, in the small number of patients studied, VAP-1 blockade did not appear to impact their IBD.

In conclusion, the BUTEO trial confirmed that 8 mg/kg timolumab was safe over the duration of administration and resulted in sufficient circulating levels of timolumab in patients with PSC. However, the trial was stopped after the interim assessment due to a lack of efficacy. Future studies of this and related molecules likely require longer duration administration and more specific and less labile surrogate markers of disease activity.

## Supplementary Material

SUPPLEMENTARY MATERIAL
