# Supplementary material for: Vascular adhesion protein-1 blockade in primary sclerosing cholangitis: Open-label, multicenter, single-arm, phase II trial
Source: Hepatol Commun. 2024 Apr 26;8(5):e0426. doi: 10.1097/HC9.0000000000000426 (PMC12333712; doi:10.1097/HC9.0000000000000426)
Supplement: SUPPLEMENTARY MATERIAL [file hc9-8-e0426-s001.pdf]

**Vascular adhesion protein-1 blockade with a monoclonal antibody in primary sclerosing cholangitis: an open-label, multi-centre, single arm, phase II trial**

**Supplementary Appendices**

Authors:

Gideon Hirschfield, Katherine Arndtz, Amanda Kirkham, Yung-Yi Chen, Richard Fox, Anna Rowe, Jessica Douglas-Pugh, Douglas Thorburn, Eleanor Barnes, Guruprasad Aithal, Diana Hull, Kushpreet Bhandal, Kathryn McQuillan, Paul Woodward, Siân Lax, Philip Newsome, David J. Smith, Antero Kallio, David Adams, Victoria Homer, Chris J Weston.

**Contents**

Supplementary appendix 1: BUTEO trial protocol .... 3

Supplementary appendix 2: Additional methods .... 4

- Trial secondary outcomes .... 4
- Measurement circulating markers of fibrosis .... 5
- Serum amine oxidase activity measurement .... 5
- Determination of circulating immune cell populations by flow cytometry .... 6
- Additional statistical analysis methods .... 8
- Fig. S2: Flow cytometry gating strategy .... 10
- References .... 11

Supplementary appendix 3: BUTEO trial statistical analysis plan .... 12

Supplementary appendix 4: Treatment compliance and analysis populations .... 13

Supplementary appendix 5: Confirmation of timolumab dose .... 15

- Table S5: All grade 3 or higher adverse events experienced by patients during the dose confirmation stage .... 15
- Fig. S5: Repeated measures of circulating timolumab concentration in all patients .... 17

Supplementary appendix 6: Secondary outcomes .... 18

- Table S6A: Serum liver tests and risk scores .... 18
- Table S6B: LiverMultiscan LIF score .... 19

Supplementary appendix 7: Circulating populations of inflammatory cells .... 20

- Fig. S7: Proportions of circulating inflammatory cells that may also contribute to the pathogenesis of PSC .... 20

Supplementary appendix 8: Markers of fibrogenesis and fibrolysis .... 21

- Fig. S8A: Liver fibrosis .... 21
- Fig. S8B: Repeated measures plot of Fibroscan measurements for all evaluable patients in the mITT population. .... 22
- Fig. S8C: Concentration of circulating markers of ECM synthesis and turnover .... 23
- Fig. S8D: Concentration of exploratory circulating markers of fibrotic tissue remodelling .... 24

**Supplementary appendix 1: BUTEO trial protocol**

# A single arm, two-stage, multi-centre, phase II clinical trial investigating the safety and activity of the use of BTT1023, a human monoclonal antibody targeting vascular adhesion protein (VAP-1), in the treatment of patients with primary sclerosing cholangitis (PSC)

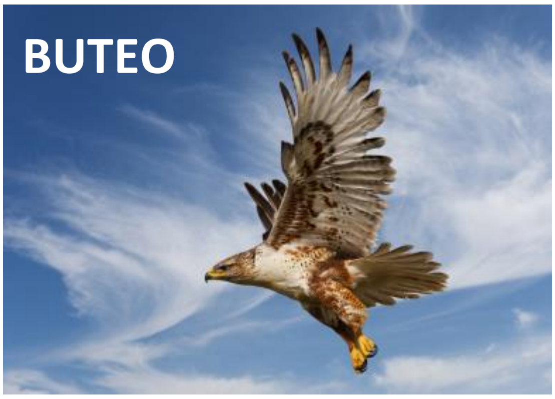


**EudraCT Number: 2014-002393-37**

**Protocol Version: 5.0 31-Jul-2018**

**Sponsor Number: RG_13-027**

**Clinicaltrials.gov Identifier: NCT02239211**

**ISRCTN Number: 11233255**

**CRCTU Number: HE2022**

## TRIAL CONTACTS

**Sponsor:** **University of Birmingham**

**Chief-Investigator:** *Professor Philip Newsome

**Co-Investigator:** *Professor David Adams
Professor Gideon Hirschfield

*Clinical Coordinators

***Professor Philip Newsome**
**Centre of Liver Research, Institute of Biomedical Research, University of Birmingham, B15 2TT**

Cancer Research UK Clinical Trials Unit

**Senior Trial Coordinator:** Dr Anna Rowe
**Trial Coordinator:** Max Kirvan
**Lead Trial Statistician:** Richard Fox
**Trial Statistician:** Amanda Kirkham

**Pharmacy Representative:** Amisha Desai

**BUTEO Trial Office**

BUTEO Trial Office
Cancer Research UK Clinical Trials Unit (CRCTU)
D3B Team, 5th Floor East,
Institute of Translational Medicine,
Heritage Building,
Birmingham
B15 2TH

**Enquiries**
☎ 0121 371 8117 or 0121 371 8158
🖷 0121 371 7246
✉ BUTEO@trials.bham.ac.uk

**Registration – Expansion Phase Only**
www.cancertrials.bham.ac.uk

**Emergency registration – Expansion Phase only**
☎ 0121 371 8158 or 0121 371 8157

**Serious Adverse Event Reporting**
🖷 0121 371 7246 (primary number)
🖷 0121 414 7989 (secondary number)
☎ 0121 371 8117 or 0121 371 8158

## CHIEF INVESTIGATOR SIGNATURE PAGE

BUTEO Trial Protocol Version 5.0 31-Jul-2018

**This protocol has been approved by:**

| | | | |
|---|---|---|---|
| **Name:** | Prof Phillip Newsome | **Trial Role:** | Chief Investigator |
| **Signature:** | [signature] | **Date:** | 01/08/2018 |

This protocol describes the BUTEO trial and provides information about procedures for patients taking part in the BUTEO trial. The protocol should not be used as a guide for treatment of patients not taking part in the BUTEO trial.

This protocol was written using CRCTU-PRT-QCD-001, version 1.0

## AMENDMENTS

The following amendments and/or administrative changes have been made to this protocol since the implementation of the first approved version

| Amendment number | Date of amendment | Protocol version number | Type of amendment | Summary of amendment |
|---|---|---|---|---|
| N/A | 18th March 2015 | 1.0a | Non-substantial amendment | - Section 7.6.1 – wording updated to prevent additional samples being taken.<br>- Grammatical errors and line spacing errors corrected. |
| 1 | 27th November 2015 | 2.0 | Substantial | - Updated contact details throughout<br>- Formatting, typographical errors and clarifications throughout<br>- Updated and clarified Inclusion and Exclusion criteria<br>- Screening period reduced to 6-8 weeks<br>- Schedule of Events updated including:<br>▪ ELF test being included as a research sample<br>▪ central ALP for Visits 3-11<br>▪ ADA, PD and QoL not performed at Screening visit 1 & 2<br>▪ MRI scans clarified<br>- 5.2 updated to include Screening Number details<br>- 6.2 updated to clarify and include Screening Number<br>- 7.3 Visit tests corrected and updated<br>- 7.5 & 7.6 updated to account for amendment changes<br>- 7.7 Clarified<br>- 7.9 Updated regarding delay to infusion<br>- 7.11 Contraception and pregnancy section added<br>- 8.1 & 8.2 clarified<br>- 9.1 Details updated and CRF list updated<br>- References updated<br>- Appendices updated |
| 2 | 16th March 2016 | 3.0 | Substantial | - Inclusion criteria for patients ALP value has been reduced from >2 x ULN to >1.5 x ULN<br>- Pre-medications updated (7.9 and where |

| | | | | |
|---|---|---|---|---|
| | | | | applicable) to include hydrocortisone at Visit 3-5<br>- Section 8.1.2 "Hypersensitivity, Infusion Reactions and Infusion Related Reactions" added<br>- Grammatical and continuity errors corrected<br>- Additional telephone number for Trials Office added throughout |
| 3 | 27th March 2018 | 4.0 | Substantial amendment | - Contact telephone and fax details updated throughout protocol<br>- 4.1 point 3 in the inclusion criteria has been clarified; validity of colonoscopy results have been altered from within 1 year to within the patient's standard of care<br>- Inclusion criteria updated and clarified (minimum weight criteria added)<br>- Footers in Schedule of Events amended to match text in written protocol<br>- Grammatical errors and line spacing errors corrected<br>- Timing of interim analysis clarified<br>- Information added regarding Acorda, Biotie's parent company<br>- Section 3.3 Clarification of replaceable patients in stage 2 of the trial<br>- Section 7.13 Deleted sentence for clarity<br>- Section 12.2 Additional sentence added for completeness; and also a sentence amended for clarity |
| 4 | 31st July 2018 | 5.0 | Substantial Amendment | - Change of CI<br>- Change of Trial Coordinator<br>- Updated text regarding GDPR |

## TRIAL SYNOPSIS

### Title

A single arm, two-stage, multi-centre, phase II clinical trial investigating the safety and activity of the use of BTT1023, a human monoclonal antibody targeting vascular adhesion protein (VAP-1), in the treatment of patients with primary sclerosing cholangitis (PSC).

### Trial Design

This is a two-stage, single-arm, open-label, multi-centre phase II trial.

### Objectives

**Primary:**

1) To determine the activity of the anti-VAP-1 antibody BTT1023 in patients with PSC as measured by a decrease in alkaline phosphatase levels (ALP) (primary endpoint) with secondary endpoints to include various measures of liver injury and fibrosis.
2) To evaluate the safety, effective dose, and tolerability of BTT1023 in patients with PSC.

**Secondary:**

1) To determine the mechanisms of action of BTT1023 through in vivo assessment of VAP-1/semicarbazide-sensitive amine oxidase (SSAO) enzyme activity and immune cell function.
2) To evaluate the potential of a novel Magnetic Resonance Imaging (MRI) based assessment of liver fibrosis and biliary strictures for assessing therapeutic response in PSC.
3) Assess the use of sVAP-1/SSAO as a biomarker to monitor disease progression in PSC.

### Outcome Measures

**Primary:**

1) Response at Day 99: a reduction in serum alkaline phosphatase (ALP) by 25% or more from baseline to Day 99.

**Secondary:**

1) Safety and tolerability
   - Treatment Compliance (including patient withdrawal) and Serious Adverse Event (SAE) and Adverse Event (AE) frequency.
2) Calculation of any change (*improvement or worsening*) from baseline to Day 99 in:
   - Quality of life questionnaires: EQ-5D, Fatigue Severity Scale, Pruritus Visual Analogue Score (VAS), Inflammatory Bowel Disease (IBD) Diaries (if applicable).
   - Tests of liver fibrosis: enhanced liver fibrosis (ELF) and Fibroscan.
   - Individual markers of liver biochemistry and function (aspartate transaminase [AST], alanine transaminase [ALT], ALP, gamma glutamyl transferase [GGT], bilirubin, Albumin, International Normalised Ratio [INR]) and composite risk scores (Mayo PSC Risk score and model for end-stage liver disease [MELD] score).

- Change (*improvement or worsening* of) in LiverMultiscan® MRI imaging – Liver MRI is an emerging method for monitoring liver disease and its treatment. We will evaluate changes in MRI imaging pre and post therapy using the LiverMultiscan® protocol (or equivalent methodology, at sites where this is possible).

3) Evaluate changes in sVAP-1/SSAO as a biomarker of liver disease activity across the trial period.

## Patient Population

Patients who have been clinically diagnosed with PSC, as confirmed in one of the trial centres, and who also meet the inclusion and exclusion criteria.

## Sample Size

The sample size is a maximum of 59 patients.

## Inclusion and Exclusion Criteria

**Inclusion criteria**

Subjects must meet *all* of the following inclusion criteria to be eligible for participation in the clinical trial:

1. Males and females 18 - 75 years of age who are willing and able to provide informed, written consent and comply with all trial requirements
2. Clinical diagnosis of PSC as evident by chronic cholestasis of more than six months duration with either a consistent MRI showing sclerosing cholangitis or a liver biopsy consistent with PSC in the absence of a documented alternative aetiology for sclerosing cholangitis
3. In those with concomitant Inflammatory Bowel Disease, clinical and colonoscopic evidence (in line with the patient's standard of care; within 18 months) of stable disease without findings of high grade dysplasia
4. In those on treatment with ursodeoxycholic acid (UDCA), therapy must be stable for at least 8 weeks and at a dose not greater than 20 mg/kg/day. In those not on treatment with UDCA at the time of screening, a minimum of 8 weeks since the last dose of UDCA should be recorded
5. Serum ALP greater than 1.5 x upper limit of normal (ULN)
6. Stable serum ALP levels (levels must not change by more than 25% from Screening Visit 1 and Screening Visit 2)
7. Female subjects of childbearing potential must have a negative pregnancy test prior to starting trial treatment. For the purposes of this trial, a female subject of childbearing potential is a woman who has not had a hysterectomy, bilateral oophorectomy, or medically-documented ovarian failure. Women ≤ 50 years of age with amenorrhea of any duration will be considered to be of childbearing potential
8. All sexually active women of childbearing potential must agree to use two forms of highly effective method of contraception from the Screening Visit throughout the trial period and for 99 days following the last dose of trial drug. If using hormonal agents the same method must have been used for at least 1 month before trial dosing and subjects must use a barrier

method as the other form of contraception. Lactating women must agree to discontinue breast feeding before trial investigational medicinal product administration

9. Men, if not vasectomised, must agree to use barrier contraception (condom plus spermicide) during heterosexual intercourse from screening through to trial completion and for 99 days from the last dose of trial investigational medicinal product
10. Patients must weigh ≥ 40 kg

**Exclusion criteria**

Subjects who meet *any* of the following exclusion criteria are excluded from participating in the BUTEO trial:

1. Presence of documented secondary sclerosing cholangitis on prior clinical investigations
2. Presence of alternative causes of liver disease, that are considered by the Investigator to be the predominant active liver injury at the time of screening, including viral hepatitis, alcoholic liver disease, non-alcoholic steatohepatitis, primary biliary cirrhosis. Patients with possible overlap syndrome with autoimmune hepatitis are excluded if the Investigator considers autoimmune hepatitis as the predominant liver injury
3. AST and ALT >10 x ULN or bilirubin >3 x ULN or INR >1.3 in the absence of anti-coagulants
4. Serum creatinine >130μmol/L or platelet count <50 x $10^{9}$/L
5. Any evidence of hepatic decompensation past or present, including ascites, episodes of hepatic encephalopathy or variceal bleeding
6. Recent cholangitis within last 90 days or ongoing need for prophylactic antibiotics
7. Pregnancy or breast feeding
8. Harmful alcohol consumption as evaluated by the Investigator
9. Flare in colitis activity within last 90 days requiring intensification of therapy beyond baseline maintenance treatment; use of oral prednisolone >10 mg/day, biologics (i.e. monoclonal antibodies) and or hospitalisation for colitis within 90 days. Prior use of biologics is not a contraindication to screening
10. Diagnosed cholangiocarcinoma or high clinical suspicion of cholangiocarcinoma either clinically or by imaging
11. Concurrent malignancies or invasive cancers diagnosed within past 3 years except for adequately treated basal cell and squamous cell carcinoma of the skin and in situ carcinoma of the uterine cervix
12. Presence of a percutaneous drain or bile duct stent
13. Major surgical procedure within 30 days of screening
14. Prior organ transplantation
15. Known hypersensitivity to the investigational product or any of its formulation excipients
16. Unavailable for follow-up assessment or concern for subject's compliance
17. Participation in an investigational trial of a drug or device within 60 days of screening or 5 half-lives of the last dose of investigational drug, where the trial drug half-life is greater than 12 days

18. Any other condition that in the opinion of the Investigator renders the subject a poor risk for inclusion into the trial
19. Positive screening test for tuberculosis (TB) (including T-SPOT.TB TB test), unless respiratory review confirms false positive test results
20. Receipt of live vaccination within 6 weeks prior to Screening Visit 2
21. Known HIV positive status

### Trial Duration

It is anticipated that recruitment will take 2 years. The treatment period is 78 days and all patients will be followed up until Day 120, i.e. 42 days after the last administration of trial treatment.

### Trials Office Contact Details

BUTEO Trial Office
Cancer Research UK Clinical Trials Unit (CRCTU)
D3B Team, 5th Floor East,
Institute of Translational Medicine,
Heritage Building,
Birmingham
B15 2TH

☎ 0121 371 8117 or 0121 371 8158

🖷 0121 371 7246

✉ BUTEO@trials.bham.ac.uk

## Trial Schema

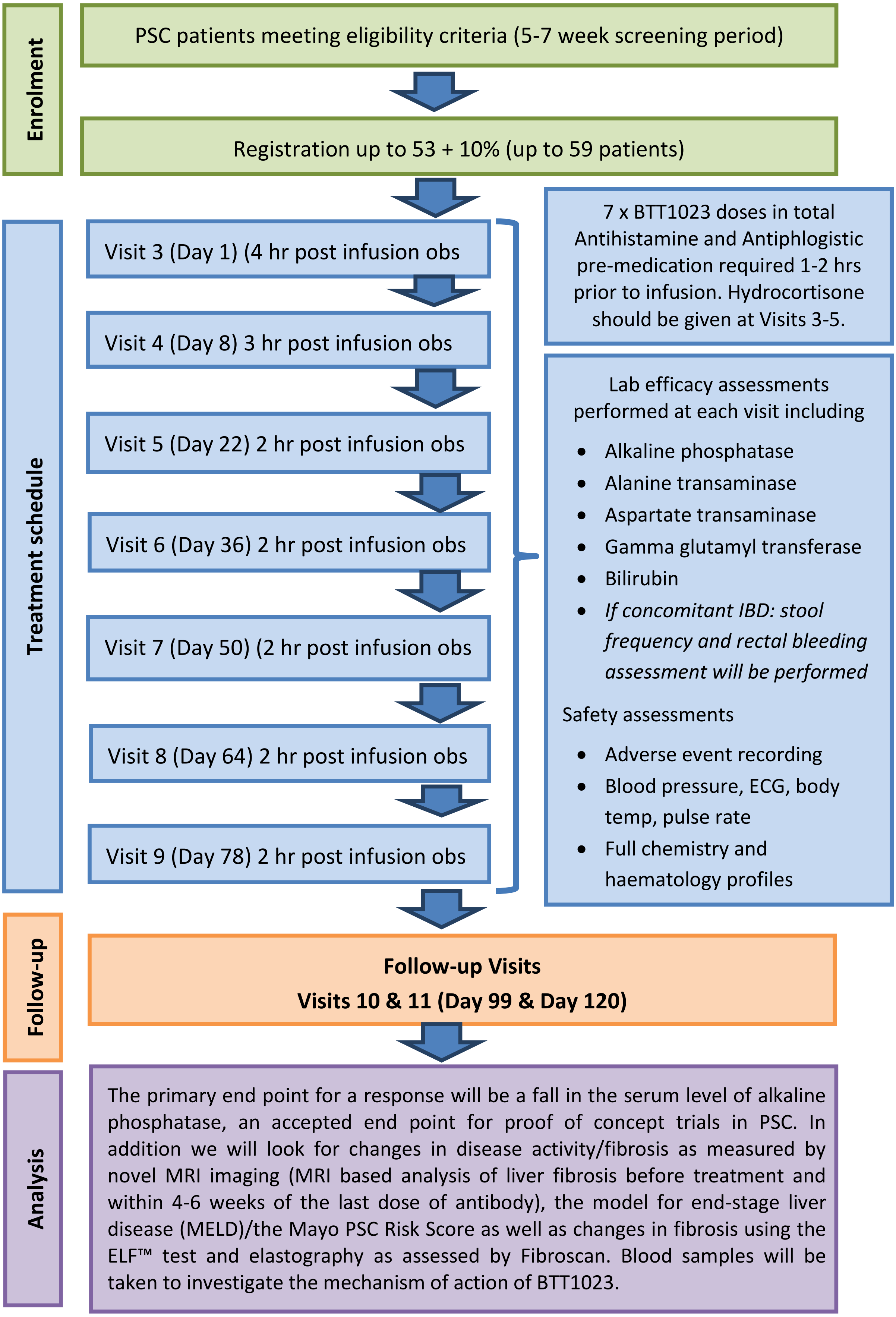

## Schedule of Events

| | Screening | | Treatment | | | | | | | Follow-up | |
|---|---|---|---|---|---|---|---|---|---|---|---|
| | Screening Visit 1 (within 5-7 weeks of Visit 3) | Screening Visit 2 (within 1 week of Visit 3) | Visit 3 (Infusion visit Day 1) | Visit 4 (Infusion visit Day 8) +/- 3 days | Visit 5 (Infusion visit Day 22) +/- 3 days | Visit 6 (Infusion visit Day 36) +/- 3 days | Visit 7 (Infusion visit Day 50) +/- 3 days | Visit 8 (Infusion visit Day 64) +/- 3 days | Visit 9 (Infusion visit Day 78) +/- 3 days | Visit 10 (Day 99) +/- 3 days | Visit 11 (Day 120) +/- 3 days |
| Informed consent | X | | | | | | | | | | |
| Eligibility assessment | X | X | | | | | | | | | |
| Patient history | X | X | | | | | | | | | |
| Physical exam | X | | X | | X | | X | | X | X | X |
| Vital signs [1] | X | | X | X | X | X | X | X | X | X | X |
| Vital signs during and post infusion [2] | | | X | X | X | X | X | X | X | | |
| Electrocardiogram [3] | X | | X | X | X | X | X | X | X | X | |
| BTT1023 infusion | | | X | X | X | X | X | X | X | | |
| Standard blood tests [4] | X | X | X | X | X | X | X | X | X | X | X |
| ALP sample for central analysis (pre infusion) | | | X | X | X | X | X | X | X | X | X |

| | Screening | | Treatment | | | | | | | Follow-up | |
|---|---|---|---|---|---|---|---|---|---|---|---|
| | Screening Visit 1 (within 5-7 weeks of Visit 3) | Screening Visit 2 (within 1 week of Visit 3) | Visit 3 (Infusion visit Day 1) | Visit 4 (Infusion visit Day 8) +/- 3 days | Visit 5 (Infusion visit Day 22) +/- 3 days | Visit 6 (Infusion visit Day 36) +/- 3 days | Visit 7 (Infusion visit Day 50) +/- 3 days | Visit 8 (Infusion visit Day 64) +/- 3 days | Visit 9 (Infusion visit Day 78) +/- 3 days | Visit 10 (Day 99) +/- 3 days | Visit 11 (Day 120) +/- 3 days |
| Standard blood tests 2 hrs post infusion [5] | | | **X** | **X** | **X** | **X** | **X** | **X** | **X** | | |
| Blood + serum sample for biomarker and research blood analysis (inc ELF) | **X** | **X** | **X** | **X** | **X** | **X** | **X** | **X** | **X** | **X** | **X** |
| Blood serum for PK [6] | | | **X** | **X** | **X** | **X** | **X** | **X** | **X** | **X** | **X** |
| Blood serum for Anti-Drug Antibodies (ADA) | | | **X**[8] | | | | **X**[8] | | **X**[8] | **X** | **X** |
| Blood serum for PD (sVAP-1) | | | **X**[9] | **X**[8] | **X**[8] | **X**[8] | **X**[8] | **X**[8] | **X**[10] | **X** | **X** |
| MRI scan [7] | **X** | | | | | | | | | | **X** |
| | | | | | | | | | | | |

| | Screening | | Treatment | | | | | | | Follow-up | |
|---|---|---|---|---|---|---|---|---|---|---|---|
| | Screening Visit 1 (within 5-7 weeks of Visit 3) | Screening Visit 2 (within 1 week of Visit 3) | Visit 3 (Infusion visit Day 1) | Visit 4 (Infusion visit Day 8) +/- 3 days | Visit 5 (Infusion visit Day 22) +/- 3 days | Visit 6 (Infusion visit Day 36) +/- 3 days | Visit 7 (Infusion visit Day 50) +/- 3 days | Visit 8 (Infusion visit Day 64) +/- 3 days | Visit 9 (Infusion visit Day 78) +/- 3 days | Visit 10 (Day 99) +/- 3 days | Visit 11 (Day 120) +/- 3 days |
| Pregnancy test (female patients of childbearing potential only) | **X** | **X** | | | **X** | | **X** | | **X** | | **X** |
| Mayo PSC Risk Score | | **X** | | | | | | | | **X** | |
| MELD score | | **X** | | | | | | | | **X** | |
| Fibroscan® | | **X** | | | | | | | | **X** | |
| T-SPOT.TB test | **X** | | | | | | | | | | |
| Quality of Life questionnaires [11] | | **X (IBD diary only if applicable)** | **X** | | | **X** | | | | **X** | |
| Adverse/ Clinical events | **X** | **X** | **X** | **X** | **X** | **X** | **X** | **X** | **X** | **X** | **X** |
| Concomitant medications | **X** | **X** | **X** | **X** | **X** | **X** | **X** | **X** | **X** | **X** | **X** |

1 Vital signs: heart rate, blood pressure, temperature and respiratory rate to be performed pre-infusion Visits 3-11.

2 Vital signs: heart rate, blood pressure, temperature and respiratory rate to be performed:

- **Visit 3:** 30 and 60 minutes after the start of the infusion, 30, 60, 90, 120, 180 and 240 minutes post end of drug infusion.
- **Visit 4:** 30 and 60 minutes after the start of the infusion, 30, 60, 90, 120 and 180 minutes post end of drug infusion.
- **Visits 5-9:** 30 and 60 minutes after the start of the infusion, 30, 90, and 120 minutes post end of drug infusion.

3 12 lead electrocardiogram (ECG) to be performed pre-infusion and 60 minutes post end of drug infusion.

4 Full blood count (FBC), urea, electrolytes, liver function tests (LFTs), eGFR (calculated by Cockcroft-Gault equation or alternative as per institutional practice – Appendix 1) international normalised ratio (INR) to be performed pre-infusion **Visits 3-9**.

5 FBC, urea, electrolytes, LFTs, INR to be performed 120 minutes after the end of infusion.

6 Blood serum for pharmacokinetics. See Tables 6-8 for timings.

7 MRI to be performed during screening period and 4-6 weeks of end of treatment (last dose of antibody). A Liver multiscan should be performed where possible (or equivalent methodology, at sites where this is possible). Where this is not possible please perform an MRI with MRCP.

8 Blood serum samples: Pre-infusion.

9 Blood serum samples: Pre-infusion, 180 and 360 minutes post infusion start and 24 hrs post end of drug infusion.

10 Blood serum samples: Pre-infusion, 120 minutes post infusion start and 24 hrs post end of drug infusion.

11 Quality of Life Questionnaires to be completed pre-infusion on Visit 3 and Visit 6. If patient has IBD, please give the IBD diary to patient at Screening Visit 2, to be completed prior to Visit 3.

**Abbreviations**

| | |
|---|---|
| ADA | Anti-Drug Antibodies |
| AE | Adverse Event |
| ALP | Alkaline Phosphatase |
| ALT | Alanine Transaminase |
| APTT | Activated Partial Thromboplastin Time |
| AST | Aspartate Transaminase |
| CRCTU | Cancer Research UK Clinical Trials Unit |
| CRF | Case Report Form |
| CTCAE | Common Terminology Criteria for Adverse Events |
| DMC | Data Monitoring Committee |
| DoH | Department of Health |
| ECG | Electrocardiogram |
| eGFR | Estimated Glomerular Filtration Rate |
| ELF | Enhanced Liver Fibrosis |
| eRDC | Electronic Remote Data Capture |
| FBC | Full Blood Count |
| GCP | Good Clinical Practice |
| GGT | Gamma Glutamyl Transferase |
| HRS | Hours |
| IBD | Inflammatory Bowel Disease |
| IFN | Interferon |
| IgG | Immunoglobulin G |
| IL | Interleukin |
| INR | International Normalised Ratio |
| IMP | Investigational Medicinal Product |
| ISF | Investigator Site File |
| LFT | Liver Function Test |
| LPLV | Last Patient Last Visit |
| MELD | Model for End-stage Liver Disease |
| MHRA | Medicines and Healthcare Products Regulatory Agency |
| MRI | Magnetic Resonance Imaging |

| | |
|---|---|
| NCI | National Cancer Institute |
| NIHR | National Institute of Health Research |
| NSAID | Non-steroidal Anti-Inflammatory Drug |
| OBS | Observations |
| PD | Pharmacodynamics |
| PK | Pharmacokinetics |
| PSC | Primary Sclerosing Cholangitis |
| QoL | Quality of Life |
| RA | Rheumatoid Arthritis |
| REC | Research Ethics Committee |
| SAE | Serious Adverse Event |
| SAR | Serious Adverse Reaction |
| SSAO | Semicarbazide-Sensitive Amine Oxidase |
| SUSAR | Suspected Unexpected Serious Adverse Reaction |
| sVAP-1 | Serum Vascular Adhesion Protein-1 |
| TMG | Trial Management Group |
| TNF | Tumour Necrosis Factor |
| UDCA | Ursodeoxycholic Acid |
| UE | Urea and Electrolytes |
| VAP-1 | Vascular Adhesion Protein-1 |
| ULN | Upper Limit of Normal |

Table of contents


**1. Background and Rationale ........ 1**
1.1 Background ........ 1
1.1.1 Existing Research ........ 1
1.1.2 Pharmacology and Toxicology of BTT1023 ........ 2
1.2 Trial Rationale ........ 3
1.2.1 Justification for Patient Population ........ 3
1.2.2 Justification for Design and Dose Levels ........ 3
1.2.3 Choice of Treatment ........ 3
**2. Aims, Objectives and Outcome Measures ........ 4**
2.1 Aims and Objectives ........ 4
2.1.1 Primary Objectives ........ 4
2.1.2 Secondary Objectives ........ 5
2.2 Outcome Measures ........ 5
**3. Trial Design ........ 5**
3.1 Dose Limiting Toxicity ........ 6
3.2 Dose Limiting Reporting Procedure ........ 8
3.3 Dose Escalation Range ........ 8
**4. Eligibility ........ 11**
4.1 Inclusion Criteria* ........ 11
4.2 Exclusion Criteria* ........ 12
**5. Consent and Screening ........ 13**
5.1 Informed Consent ........ 13
5.2 Screening ........ 13
**6. Trial Entry ........ 15**
6.1 Pre-registration ........ 15
6.2 Patient Registration ........ 15
6.2.1 Patient Registration During Dose Confirmation Phase ........ 15
6.2.2 Patient Registration During Dose Expansion Phase (Via Electronic Remote Data Capture System) 16
6.3 Emergency Registration ........ 16
**7. Treatment Details ........ 16**
7.1 Investigational Medicinal Product Preparation ........ 16
7.1.1 Dosage Form and Composition ........ 17
7.1.2 Instructions for Storage ........ 17
7.1.3 Instructions for Handling the Investigational Medicinal Product ........ 17
7.2 Trial Treatment ........ 17
7.3 Treatment Schedule (Visit 3 – Visit 9) ........ 18
7.4 Trial Schema ........ 22
7.5 Assessments ........ 23
7.5.1 Blood Chemistry and Haematology ........ 23

7.5.2 Pharmacokinetics Scheduling ........ 24
7.5.3 Physical Examination/Symptom Assessment ........ 25
7.5.4 Mayo Risk Assessment ........ 25
7.6 Sample Collection ........ 25
7.6.1 Blood Samples ........ 25
7.6.2 Pharmacokinetic, Anti-Drug Antibodies and Pharmacodynamic Samples ........ 25
7.7 Dose Modifications ........ 25
7.8 Treatment Compliance ........ 26
7.9 Pre-Medication ........ 26
7.10 Concomitant Medication ........ 26
7.11 Contraception and Pregnancy ........ 26
7.12 Patient Follow Up ........ 27
7.13 Patient Withdrawal ........ 27
**8. Adverse Event Reporting ........ 28**
8.1 Reporting Requirements ........ 28
8.1.1 Adverse Events ........ 29
8.1.2 Hypersensitivity, Infusion Reactions and Infusion Related Reactions ........ 29
8.1.3 Serious Adverse Events ........ 30
8.1.4 Reporting Period ........ 30
8.2 Reporting Procedure ........ 30
8.2.1 Site ........ 30
8.2.2 Trials Office ........ 31
8.2.3 Reporting to the Competent Authority and Research Ethics Committee ........ 31
8.2.4 Investigators ........ 32
8.2.5 Data Monitoring Committee ........ 32
8.2.6 Provider of Investigational Medicinal Product ........ 32
**9. Data Handling and Record Keeping ........ 32**
9.1 Data Collection ........ 32
9.2 Archiving ........ 35
**10.Quality Management ........ 35**
10.1 Site Set-up and Initiation ........ 35
10.2 On-site Monitoring ........ 36
10.3 Central Monitoring ........ 36
10.4 Audit and Inspection ........ 36
10.5 Notification of Serious Breaches ........ 36
**11.End of Trial Definition ........ 37**
**12.Statistical Considerations ........ 37**
12.1 Definition of Outcome Measures ........ 37
12.1.1 Primary Outcome Measure ........ 37
12.1.2 Secondary Outcome Measures ........ 37

12.2 Analysis of Outcome Measures ..... 37
12.3 Planned Sub Group Analyses ..... 38
12.4 Planned Interim Analysis ..... 38
12.5 Planned Final Analyses ..... 38
12.6 Power Calculations ..... 38
**13.Trial Organisational Structure ..... 39**
13.1 Sponsor ..... 39
13.2 Coordinating Centre ..... 39
13.3 Trial Management Group ..... 39
13.4 Trial Steering Committee ..... 39
13.5 Data Monitoring Committee ..... 39
13.6 Finance ..... 40
**14.Ethical Considerations ..... 40**
**15.Confidentiality and Data Protection ..... 40**
**16.Insurance and Indemnity ..... 41**
**17.Publication Policy ..... 42**
**18.Reference List ..... 43**
**Appendix 1 – Equation For Estimated Glomerular Filtration Rate ..... 44**
**Appendix 2 - Common Toxicity Criteria Gradings ..... 45**
**Appendix 3 – T-SPOT.TB ..... 46**
**Appendix 4 - Definition of Adverse Events ..... 48**
**Appendix 5 - WMA Declaration of Helsinki ..... 50**

**LIST OF FIGURES**

**Figure 1:** Flow diagram showing the trial decision guidelines for the run-in period of the trial design incorporating a conventional 3+3 cohort design if dose escalation is required. ..... 10
**Figure 2:** Trial/Patient Flow Diagram ..... 22

**LIST OF TABLES**

**Table 1:** Suspected DLT Procedure ..... 7
**Table 2:** Dose Level ..... 9
**Table 3:** BTT1023 Drug Infusion Table ..... 18
**Table 4:** Blood Tests ..... 23
**Table 5:** Immunology Blood Tests ..... 24
**Table 6:**Visit 3+9 Pharmacokinetics Scheduling ..... 24
**Table 7:** Visit 4 Pharmacokinetics Scheduling ..... 24
**Table 8:** Visit 5-8 Pharmacokinetic Scheduling ..... 25
**Table 9:** Description of Case Report Forms ..... 33
**Table 10:** Possible Stop/Go Guidelines and Associated Error Rates ..... 38

# 1. BACKGROUND AND RATIONALE

## 1.1 Background

End-stage liver disease, regardless of aetiology, is characterised by hepatic fibrosis culminating in liver cirrhosis and accompanying increasing risks of liver cancer, liver failure, portal hypertension and death. Preventing progressive liver fibrosis represents an important area of interest in the development of new drugs suitable for all patients with liver disease. Primary sclerosing cholangitis (PSC) is a prime example of a progressive inflammatory liver disease which is characterised by relentless liver fibrosis and a high unmet need for new therapies. PSC has a population incidence of 1.3/100,000 annually, with a prevalence of 16.2 per 100,000. It affects men and women with a median age of 41 and in 80% of cases is associated with Inflammatory Bowel Disease (IBD). More than 50% of patients require liver transplantation within 10-15 years of symptomatic presentation, reflecting the failure of medical therapies to make any impact on the clinical outcome. The progression of PSC to scarring, cirrhosis and hepatobiliary cancer is driven by a chronic inflammatory response and immune cell mediated destruction of bile ducts (Hirschfield, Karlsen *et al*. 2013). Our research implicates vascular adhesion protein-1 (VAP-1) in the inflammation that drives fibrogenesis in liver disease (Lalor, Edwards *et al.* 2002) and we have shown levels of sVAP-1 in serum are elevated in chronic liver diseases and correlate with histological fibrosis (Kurkijärvi, Adams *et al*. 1998, Kurkijärvi, Yegutkin *et al.* 2000). These observations underpin our proposal that VAP-1 has an important role in the progression of liver fibrosis. We now plan to test the hypothesis that inhibiting VAP-1 with a neutralising antibody (BTT1023) will reverse or delay fibrogenesis in patients with, PSC and to investigate the potential of VAP-1 as a biomarker in liver disease.

### 1.1.1 Existing Research

The homodimeric transmembrane sialoglycoprotein,VAP-1,is expressed in the vasculature where it functions as an adhesion molecule that mediates the transmigration of leukocytes from blood through the endothelial lining into surrounding tissues (Jalkanen and Salmi 2001). It possesses amine oxidase activity, known as semicarbazide-sensitive amine oxidase (SSAO) and in its soluble form accounts for most amine oxidase activity in human serum (Kurkijärvi, Yegutkin *et al.* 2000). Our experimental data show that VAP-1 acts as an adhesion receptor to support leucocyte recruitment in liver inflammation. In animal models this function is critical during fibrogenesis, when it is secreted by stellate cells in response to inflammatory stimuli. It helps position inflammatory cells and activated myofibroblasts in areas of ongoing fibrogenesis (unpublished). Thus, inhibition of VAP-1 is expected to impact both inflammation and fibrosis: of note treatment with an antibody against VAP-1 prevents fibrosis in murine models of liver injury. Based on the strong up-regulation of hepatic VAP-1 in PSC patients, we hypothesise that levels of sVAP-1 will correlate with the severity of fibrosis in PSC and predict patients at risk of progressive disease. We propose that BTT1023 will work in PSC by inhibiting leucocyte recruitment into the inflamed liver. Additionally, our findings that hepatic expression of VAP-1 and its release from the liver into blood are markedly increased in chronic inflammatory liver disease suggest it may act as an organ-specific marker of liver inflammation. Our unpublished data show particularly high levels of VAP-1 in patients with PSC, with another study (Weston, Shepherd *et al.* 2015) showing a strong correlation between sVAP-1/SSAO activity in serum and histological fibrosis scores in patients with fatty liver disease. These findings suggest that circulating sVAP-1 may correlate with hepatic fibrosis stage. Our data further suggests stellate cells

secrete sVAP-1 in response to inflammatory stimuli (unpublished). Based on the strong up-regulation of hepatic VAP-1 we have reported in PSC patients (Liaskou, Karikoski *et al.* 2011), we hypothesise that levels of sVAP-1/SSAO will correlate with the severity of fibrosis in PSC and predict patients at risk of progressive disease.

### 1.1.2 Pharmacology and Toxicology of BTT1023

BTT1023 is a fully human, monoclonal, anti-VAP-1 antibody. It blocks the adhesion function of VAP-1 thereby diminishing leukocyte entry into sites of tissue inflammation, and is thought to alleviate inflammatory and fibrotic conditions. BTT1023 is a monoclonal IgG4 antibody that has been produced in a mammalian cell line and is delivered as concentrate for solution for infusion at a strength of 20 mg/ml. BTT1023 binds human recombinant and soluble VAP-1 with equilibrium dissociation constants of 130-210 pM. *In vitro,* BTT1023 effectively reduces the chemo attractant-evoked migration of lymphocytes through an endothelial cell monolayer. BTT1023 does not affect the semicarbazide-sensitive amine oxidase activity of VAP-1. *In vivo*, blocking VAP-1 function with an anti-mouse VAP-1 antibody significantly alleviates inflammation in mouse models of arthritis and lung inflammation. Treatment with an antibody against VAP-1 also prevents hepatic fibrosis in murine models of liver injury. Based on a tissue cross-reactivity study, BTT1023 recognises human and cynomolgus monkey tissues similarly, with VAP-1 expression detected mainly in vascular endothelia, liver sinusoidal endothelium, smooth muscle, adipose tissue and alveolar wall. Deducing the elimination rate constant for BTT1023 has not so far been possible in the primate studies. Based on the accumulation upon repeated dosing, the elimination half-life appears in the range of 10-20 days in the cynomolgus monkey. Intravenous administration of BTT1023 weekly for four weeks at 10, 30 or 100 mg/kg and biweekly for 14 weeks at 4, 20 or 100 mg/kg was well tolerated in cynomolgus monkeys. No toxicologically relevant effects were observed in in-life recordings or laboratory tests, or in macroscopic or histopathology. Toxicokinetic monitoring showed an adequate exposure throughout the studies. The estimated no observed adverse effect level was 100 mg/kg when administered biweekly for 14 weeks (Biotie Therapies Corp., 2008).

No adverse safety pharmacological effects were detected in conjunction with the toxicity studies when cardiovascular and ophthalmic signs were investigated. BTT1023 was free of irritant effects in both repeat dose toxicity studies. Secondary antibodies were not detected in the 4-week toxicity study. In the 14-week study an immune response against BTT1023 was detected in 7/10 animals in the low 4 mg/kg dose group on day 85. BTT1023 does not stimulate cytokine release in the cynomolgus monkey *in vivo* or from human whole blood *in vitro*. As an IgG molecule, BTT1023 is not anticipated to interfere with chromosomal material. Teratogenicity has not been studied. In the first-in-man trial with BTT1023, infusion-related adverse events (AEs) were seen in two out of five healthy volunteers in the highest dose (2 mg/kg) cohort and included flushing, tingling, numbness and oedema. No cytokine release syndrome was recorded. In subsequent trials up to 8 mg/kg of BTT1023 was dosed to patients with rheumatoid arthritis or psoriasis after a premedication (cetirizine 10 mg, ibuprofen 600 mg orally). The frequency and the intensity of the AEs observed were not increased with dose increments and BTT1023 appeared safe and well-tolerated in repeated intravenous dosing. In a three-month trial in patients with rheumatoid arthritis, 6/10 patients treated with 4 or 8 mg/kg BTT1023 experienced a 50% or greater reduction in disease activity according to American College for Rheumatology response criteria during the treatment period and 4/10 had maintained this response when assessed a month after the last dose. No patient on placebo met these criteria (Biotie Therapies Corp., 2010).

Similarly to other therapeutic proteins, BTT1023 may be immunogenic, but this has not been confirmed to date. No specific antidote to BTT1023 is available.

## 1.2 Trial Rationale

### 1.2.1 Justification for Patient Population

PSC is a progressive immune mediated biliary disease characterised by bile duct inflammation and fibrosis and accompanying hepatic fibrosis. For patients with elevated alkaline phosphatase (ALP) in particular, progressive disease is predicted, which currently results in a need for liver transplantation in the majority of cases. No current medical therapy has as yet been shown to be effective in altering the natural process of the disease. For this reason patients with PSC who have elevated ALP values will be recruited to this trial, in order to evaluate the impact of VAP-1 blockade by BTT1023, in an early phase trial focused on biochemical efficacy and safety.

### 1.2.2 Justification for Design and Dose Levels

This is an early phase trial of BTT1023 in immune mediated liver disease, with the rationale to identify biochemical efficacy of effect (reduction in ALP) and safety, in an orphan disease for PSC that presently lacks any other medical therapy. The trial design therefore focuses on identifying early biochemical efficacy signals to justify larger scale, randomised controlled trials of a longer duration.

The selection of the proposed dose and dosing interval for the trial is based on information obtained from previous multiple-dose clinical trials with BTT1023. The proposed dose level of 8 mg/kg was well tolerated by RA and psoriasis patients in previous clinical trials (BTT12-CD015and BTT2-CD016 respectively), and is therefore considered to provide appropriate bridging into existing clinical data.

Due to its mechanism of action, it is likely that a therapeutic response with BTT1023 will be best achieved through continuous exposure at therapeutic plasma levels. Consequently, the preferred dosing regimen should ensure saturation of the endogenous VAP-1 antigen pool as rapidly as possible. In the RA trial, patients received five 8 mg/kg doses of BTT1023 administered at biweekly intervals. With this dosing regimen, potentially efficacious trough concentrations of BTT1023 were observed by day 42 (i.e. before the $4^{th}$ infusion). In an attempt to accelerate saturation of the endogenous VAP-1 antigen pool, a more rapid loading scheme was utilised in the psoriasis trial, in which patients received three doses of BTT1023 on days 1, 8 and 22. Due to the limited amount of pharmacokinetic data, a detailed comparison of exposures reached with the two dosing regimens is not feasible. Most importantly however, both administration schemes were well tolerated, with no infusion-related adverse events reported in either trial. Therefore, it is proposed that the accelerated dosing scheme (i.e. dosing on days 1, 8, and biweekly thereafter) be deployed in the current study.

### 1.2.3 Choice of Treatment

A key factor in establishing and maintaining chronic inflammation is the excessive accumulation of leukocytes in tissue after they exit the blood and move towards the site of inflammation through a series of processes which include attachment to specific adhesion proteins. VAP-1 is one such adhesion molecule up-regulated principally at the site of inflammation, limiting potential side effects of a product targeting this pathway. Our research in particular implicates VAP-1 in progressive fibrosis in liver disease and we would now like to test the hypothesis that inhibiting VAP-1 with a neutralising antibody (BTT1023) will reverse or delay fibrogenesis in patients with PSC. A single arm, rather than placebo controlled design, was chosen to allow efficient enrolment of patients into the

study due to its intensive nature in which a significant chance of being allocated into the placebo group may act as a substantial barrier for enrolment. The proposed primary endpoint (ALP) is a biochemical measurement and thus not subject to subjective bias as a clinical assessment would be, rendering the necessity of a concurrent control group less vital. Our data on stability of ALP in PSC suggests that responses, as defined in the protocol, occur very seldom during the natural course of the disease, and we can therefore reliably assess changes from baseline and response rates for this proof of concept study to evaluate the therapeutic potential of the investigational product. In addition we will measure serial ALP levels before starting treatment, at Day 1, and exclude patients in whom the levels change significantly (>25%). ALP levels will also be measured at Visit 10 (Day 99) which is 21 days after the last treatment on Visit 9 (Day 78). Toxicology data with BTT1023 is available for up to 14 weeks, limiting dosing of BTT1023 to patients to three months.

We also want to investigate the potential of soluble VAP-1 as a biomarker of liver disease. The rationale for this study is based on our findings that VAP-1 release from the liver into blood is markedly increased in chronic inflammatory liver disease suggesting it may act as an organ-specific marker of liver inflammation (Kurkijärvi, Yegutkin *et al.* 2000). Our recent data shows particularly high levels in patients with PSC. In another study submitted for publication we found a strong correlation between sVAP-1/SSAO activity in serum and histological fibrosis scores in patients with fatty liver disease. This suggests that circulating sVAP-1/SSAO may correlate with hepatic fibrosis stage. Based on the strong up-regulation of hepatic VAP-1 we have previously reported in PSC patients, we hypothesise that levels of sVAP-1/SSAO will correlate with the severity of fibrosis in PSC and predict patients at risk of progressive disease. Reliable biomarkers that correlate with fibrosis stage and progression of liver disease are in demand in order to predict outcome and to stage disease without the need for invasive liver biopsy. This research will allow us to translate laboratory research into a proof of activity clinical trial incorporating experimental medicine studies that will elucidate the role of VAP-1 in liver fibrosis and its potential as a therapeutic target and biomarker.

## 2. AIMS, OBJECTIVES AND OUTCOME MEASURES

### 2.1 Aims and Objectives

The overall aims of the trial are:

1) To determine the short term activity and safety of an anti-VAP-1 antibody BTT1023 in patients with PSC
2) To confirm the safe and effective dose of BTT1023 in patients with PSC
3) To provide insights into the mechanisms of action of VAP-1/SSAO
4) To create the framework for subsequent larger scale interventions in chronic liver disease with anti-VAP-1 antibody
5) To develop soluble VAP-1/SSAO as a biomarker for liver disease by correlating serum VAP-1 (sVAP-1/SSAO) levels to liver fibrosis, severity of inflammation and clinical outcome

#### 2.1.1 Primary Objectives

1) To determine the activity of the anti-VAP-1 antibody BTT1023 in patients with PSC as measured by a decrease in ALP levels (primary endpoint) with secondary endpoints to include various measures of liver injury and fibrosis

2) To evaluate the safety, effective dose and tolerability of BTT1023 in patients with PSC

### 2.1.2 Secondary Objectives

1) To determine the mechanisms of action of BTT1023 through in vivo assessment of VAP-1/ (SSAO) enzyme activity and immune cell function
2) To evaluate the potential of a novel MRI based assessment of liver fibrosis and biliary strictures for assessing therapeutic response in PSC
3) Assess the use of sVAP-1/SSAO as a biomarker to monitor disease progression in PSC

## 2.2 Outcome Measures

**Primary:**

1) Response at Visit 10 (Day 99): a reduction in serum ALP by 25% or more from baseline to Visit 10 (Day 99)

**Secondary:**

1) Safety and tolerability:
   - Treatment Compliance (including patient withdrawal) and Serious Adverse Event (SAE) and Adverse Event (AE) frequency
2) An improvement from baseline to Visit 10 (Day 99) in:
   - Quality of life questionnaires: EQ-5D, Fatigue Severity Scale, Pruritus Visual Analogue Score (VAS), IBD Diaries (if applicable)
   - Quality of life questionnaires: EQ-5D, Fatigue Severity Scale, Pruritus Visual Analogue Score (VAS), IBD Diaries (if applicable)
   - Tests of liver fibrosis: Enhanced Liver Fibrosis (ELF) and Fibroscan
   - Individual markers of liver biochemistry and function (AST, ALT, ALP, GGT, Bilirubin, Albumin, INR) and composite risk scores (Mayo PSC Risk score and MELD)
   - Change (improvement) in LiverMultiscan® MRI imaging (or equivalent methodology, at sites where this is possible) – Liver MRI is an emerging method for monitoring liver disease and its treatment. We will evaluate changes in MRI imaging pre and post therapy using the LiverMultiscan® protocol
3) Evaluate changes in sVAP-1/SSAO as a biomarker of liver disease activity across the trial period

# 3. TRIAL DESIGN

This is a two-stage single-arm, open-label, multi-centre hybrid trial. Data from previous studies of RA and psoriasis patients supports the use of a therapeutic dose of 8 mg/kg which was well tolerated. The trial is comprised of two components – an initial run-in period to confirm the therapeutic dose, which leads into a Simon's two-stage design. The success criteria for transition from the dose confirmation period to the Simon's two-stage design have been approximated as reaching a trough concentration of 3 µg/ml free circulating BTT1023 at 8 weeks from first infusion, which is about 100 fold the dissociation constant ($K_d$) of BTT1023 from VAP-1 and should result in target occupancy of approximately 90%, with either the starting dose of 8 mg/kg or following dose escalation up to a maximum of 16 mg/kg.

## 3.1 Dose Limiting Toxicity

A Dose Limiting Toxicity (DLT) is defined as an AE that meets the criteria of grade 4 or grade 5 as defined in the Common Terminology Criteria for Adverse Events (CTCAE V4.0) (Appendix 2). However, if a definite diagnosis of cytokine release syndrome is made, it will be classed as a DLT at grade 3 and above* and must be considered at least possibly related to BTT1023.

Although previous studies have shown no DLTs to be expected with BTT1023, toxicity monitoring will be ongoing throughout the trial.

During the confirmatory stage the DLT reporting period is defined as the treatment period from treatment Visit 3 up to Visit 10 (Day 99 follow up visit).

The decision process upon notification of a second suspected DLT will be made in accordance to Table 1 below.

**In order to confirm the case of cytokine release syndrome, an additional blood sample will need to be taken for cytokine assay (Tumour necrosis factor-alpha (TNF-α), Interferon-gamma (IFN-γ), Interleukin-6 (IL-6). This information is to be provided as part of the SAE form.*

**Table 1:** Suspected DLT Procedure.

| Stage | Description | Action |
|---|---|---|
| 1 | Notification of second potential DLT received by trial team | - All relevant medical data is received and reviewed by clinical coordinator according to standard SAE timelines (7 or 15 days)<br>NB: it is encouraged that the team will meet and report all DLTs to the Data Monitoring Committee (DMC) as and when they occur depend on the timing (interval between DLTs) it may be necessary to have more frequent DMC meetings or discuss both DLTs at the same time. |
| 2 | Formal confirmation of 2nd DLT by designated clinical coordinator (member of Trial Management Group [TMG]) | - Recruitment is temporarily suspended due to safety concerns<br>- Sites immediately informed / local R&D offices informed<br>NB: any patients currently receiving therapy will continue to receive therapy at the local treating clinician's discretion, in consultation with the clinical coordinator. This decision is to be documented in the patient record. |
| 3 | DMC meeting called to review all safety information | - Arranged within 14-21 days ideally (arranged by TMG)<br>- Safety report provided to DMC by TMG<br>- DMC Chair- person to make formal recommendation to TSC/TMG |
| 4 | Trial Steering Committee (TSC) meeting to be arranged | - To review DMC recommendations (within 7-21 days of DMC meeting) |
| 5 | Inform Medicines and Healthcare products Regulatory Authority (MHRA) and Research Ethics Committee (REC) of decision if trial to close due to safety concerns is reached | - All sites will be informed immediately (within 24 hrs) by the TMG upon notification of the recommendation. Recruitment into the trial will stop with immediate effect<br>- MHRA and REC will be informed of permanent closure of the trial in accordance with UK regulations<br>- Specific circumstances of patients still receiving therapy will be assessed on an individual basis with the clinical coordinator and the local trial team. A decision will be made whether to continue therapy for the individual concerned and documented in the medical records |

## 3.2 Dose Limiting Reporting Procedure

On becoming aware that a patient has experienced a DLT, the Investigator (or delegate) must complete, date and sign an SAE Form (ensuring the DLT section is completed appropriately). The form should be faxed together with a SAE Fax Cover Sheet to the Trials Office using one of the numbers listed below as soon as possible and no later than 24 hrs after first becoming aware of the event:

To report a DLT, fax the completed SAE Form with an SAE Fax Cover Sheet to:

**0121 371 7246 (primary number)**

**0121 414 7989 (secondary number)**

NB: in addition to the above, sites are requested to directly contact the Trials Office, via phone (0121 371 8117 or 0121 371 8158) or e-mail (BUTEO@trials.bham.ac.uk) stating a suspected DLT has occurred.

## 3.3 Dose Escalation Range

Dose escalation of BTT1023 is dependent upon the levels of BTT1023 in circulating blood serum. Data previously obtained from studies in RA and psoriasis patients indicates that a therapeutic dose of 8 mg/kg should be effective. However, if successful pharmacokinetic criteria are not met following exposure at 8 mg/kg, the doses of BTT1023 will be escalated to 12 or 16 mg/kg as appropriate. In the event that the 16 mg/kg is tested and found not to meet the success criteria then the trial will stop. If, following a dose escalation, there is evidence of concerns regarding toxicity, an intermediate dose may be chosen for clinical evaluation. Any subsequent change to dose levels outside those specified in protocol (8, 12 and 16 mg/kg) will need approval by the DMC. A substantial amendment will also need to be prepared and approved by the national ethics and regulatory authorities before this intermediate dose can be clinically evaluated.

**Run-In Dose Confirmatory Period**

The run-in component of the trial has the potential to incorporate a conventional 3+3 cohort design with decision guidelines based on toxicity and pharmacokinetic (PK) data. The flow diagram in Figure 1, illustrates the trial's progression during the run-in period, specifying decision stages based on observed patient results. The trial begins with the recruitment of 6 patients all starting on the treatment dose of 8 mg/kg. During Visit 7 (Day 50), blood tests will be carried out to measure the serum BTT1023 to assess circulating PK levels at trough.

Recruitment will be paused after the sixth patient while awaiting PK results (this should take no longer than 7 weeks) and until the DLT reporting period is completed for each patient (up to Visit 10 [Day 99]). Toxicity monitoring will be on-going continually throughout the duration of the trial. An acceptable DLT rate has been established for the trial as a maximum of 1 incidence in 6 patients (~17%).

If the DLT rate rises to 2 or more, at any stage during the DLT reporting period, (Visit 3 [Day 1] to Visit 10 [Day 99]) the trial will be halted in consultation with the DMC (see Table 1).

The trial will continue into Phase II if results from the initial cohort of 6 patients, on the 8 mg/kg dose, show an acceptable DLT rate and trough levels of BTT1023 meet the stipulated success criteria of 3 µg/ml free circulating antibody. In the event that DLT rate is acceptable but the PK levels do not meet the success criteria then the trial moves into a conventional 3+3 design. In this event, the original 6 patient cohort will no longer be evaluated, but a new cohort of 3 patients will be recruited to receive the newly identified test dose. If there are no DLTs at Visit 10 [Day 99], an additional cohort of 3 patients will be recruited at the new test dose. During Visit 6 [Day 50], blood tests will be carried out to assess the level of serum BTT1023. If the circulating level of BTT1023 is found to be too low there are 2 potential dose escalation increments of 12 and 16 mg/kg (Table 2), and if it is found to be too high (such as resulting in trough levels consistently exceeding 100 µg/ml) then there is potential to de-escalate the dose in agreement with the DMC.
In the escalation phase, if a patient does not complete the Visit 7 (Day 50) visit, they will be replaced.
Once a confirmed dose has been established, the trial will continue into Phase II, continuing to include those patients who have previously been receiving the confirmed dose. Those patients not receiving the confirmed dose will not be included in the Phase II evaluation. If dose escalation occurs and the maximum dose of 16 mg/kg is utilised and found not to result in sufficient blood levels of BTT1023 then the trial will be stopped. The trial will be stopped at any stage where patient safety is compromised.

**Table 2:** Dose Level

| Dose Level | Dose |
|---|---|
| 1 | 8 mg/kg |
| 2 | 12 mg/kg |
| 3 | 16 mg/kg |

**Phase II – Single-arm Simon's 2-stage design**

The trial design incorporates an interim analysis of the accumulating data. The interim analysis (stage-1) takes place once 18 patients have been evaluated for the primary outcome – which is based on the response level of ALP. If three or more responses are observed in stage-1 then the trial will continue into stage-2. Recruitment will not be halted while stage-1 is assessed. Further patients will be recruited during stage-2 in order to obtain the required sample size of 37 patients. This figure will be permitted to increase to a total of 41 recruited patients in order to allow for a 10% patient drop-out rate - where patients will be replaced if they fail to receive any dose of BTT1023.
If overall there are nine or more responses from 37 evaluable patients then we conclude that the treatment warrants further investigation. If the prescribed patient number is not met then the design parameters will be re-evaluated. Patients treated at the confirmed dose during the run-in period will contribute to the total patient requirement.

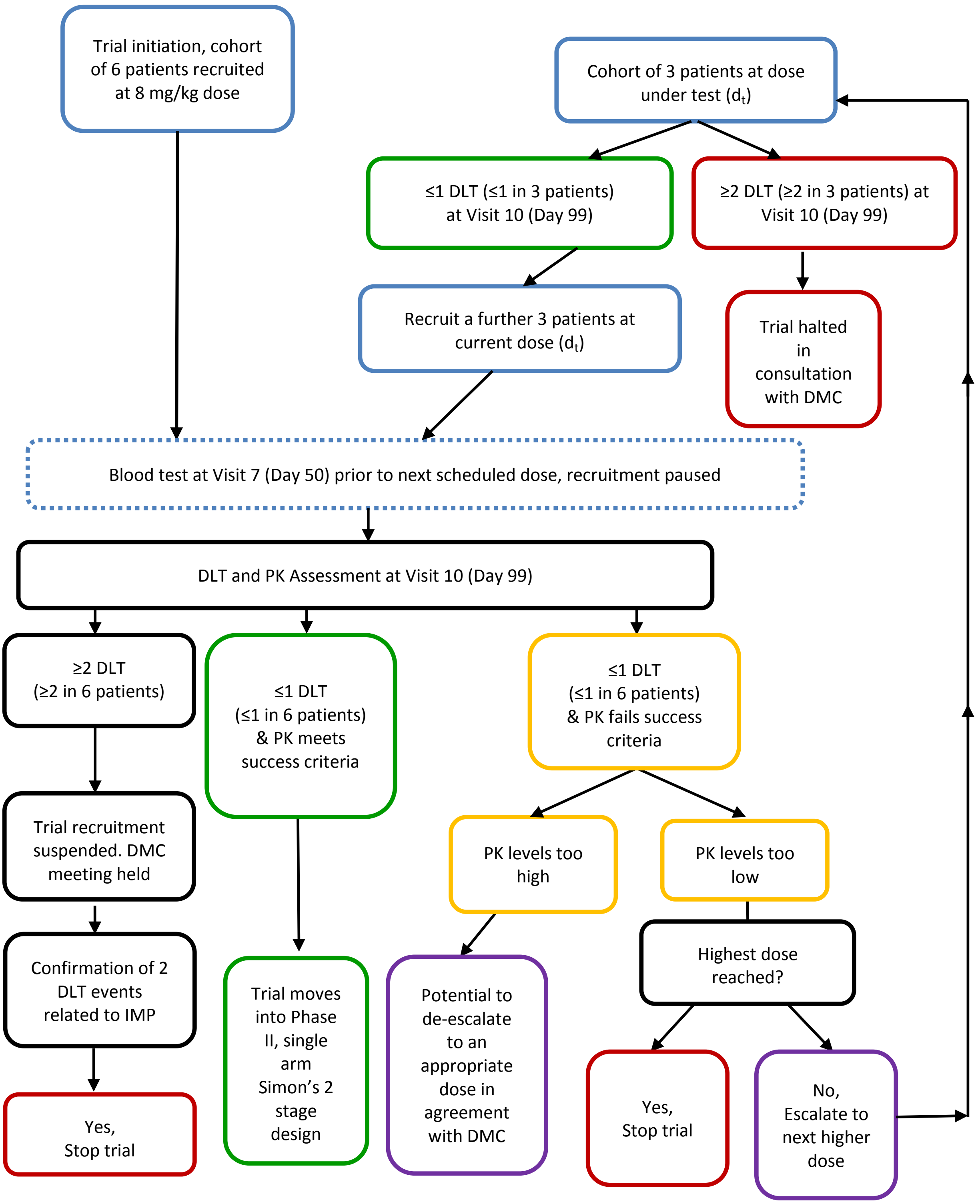


**Figure 1:** Flow diagram showing the trial decision guidelines for the run-in period of the trial design incorporating a conventional 3+3 cohort design if dose escalation is required.

To enable collection of long-term safety data, if in the opinion of the DMC a sufficiently positive risk-benefit profile has been observed (a clinically meaningful decrease in ALP for individual subjects in the absence of significant safety signals in the trial population), subjects may (pending regulatory approval of an appropriate Substantial Amendment) participate in an open label extension treatment phase not to exceed 1 year in duration. During the open label extension phase, dose level, safety monitoring, safety reporting, and treatment stopping criteria will remain the same as during the initial 11-week treatment phase of the trial.

# 4. ELIGIBILITY

## 4.1 Inclusion Criteria

Subjects must meet ***all*** of the following inclusion criteria to be eligible for participation in the clinical trial:

1. Males and females 18 - 75 years of age who are willing and able to provide informed, written consent and comply with all trial requirements
2. Clinical diagnosis of PSC as evident by chronic cholestasis of more than six months duration with either a consistent MRI showing sclerosing cholangitis or a liver biopsy consistent with PSC in the absence of a documented alternative aetiology for sclerosing cholangitis
3. In those with concomitant Inflammatory Bowel Disease, clinical and colonoscopic evidence, (in line with the patient's standard of care; within 18 months) of stable disease, without findings of high grade dysplasia
4. In those on treatment with UDCA, therapy must be stable for at least 3 months, and at a dose not greater than 20 mg/kg/day. In those not on treatment with UDCA at the time of screening, a minimum of 8 weeks since the last dose of UDCA should be recorded
5. Serum ALP greater than 1.5 x ULN
6. Stable serum ALP levels (levels must not change by more than 25% from Screening Visit 1 and Screening Visit 2)
7. Female subjects of childbearing potential must have a negative pregnancy test prior to starting trial treatment. For the purposes of this trial, a female subject of childbearing potential is a woman who has not had a hysterectomy, bilateral oophorectomy, or medically-documented ovarian failure. Women ≤ 50 years of age with amenorrhea of any duration will be considered to be of childbearing potential
8. All sexually active women of childbearing potential must agree to use two forms of highly effective method of contraception from the Screening Visit throughout the trial period and for 99 days following the last dose of trial drug. If using hormonal agents the same method must have been used for at least 1 month before trial dosing and subjects must use a barrier method as the other form of contraception. Lactating women must agree to discontinue breast feeding before trial investigational medicinal product administration
9. Men, if not vasectomised, must agree to use barrier contraception (condom plus spermicide) during heterosexual intercourse from screening through to trial completion and for 99 days from the last dose of trial investigational medicinal product
10. Patients must weigh ≥ 40 kg

## 4.2 Exclusion Criteria

Subjects who meet *any* of the following exclusion criteria are excluded from participating in the BUTEO trial:

1. Presence of documented secondary sclerosing cholangitis on prior clinical investigations
2. Presence of alternative causes of liver disease, that are considered by the Investigator to be the predominant active liver injury at the time of screening, including viral hepatitis, alcoholic liver disease, non-alcoholic steatohepatitis, primary biliary cirrhosis. Patients with possible overlap syndrome with autoimmune hepatitis are excluded if the Investigator considers autoimmune hepatitis as the predominant liver injury
3. AST and ALT >10 x ULN or bilirubin >3 x ULN or INR >1.3 in the absence of anti-coagulants
4. Serum creatinine >130µmol/L or platelet count <50 x 109/L
5. Any evidence of hepatic decompensation past or present, including ascites, episodes of hepatic encephalopathy or variceal bleeding
6. Recent cholangitis within last 90 days or ongoing need for prophylactic antibiotics
7. Pregnancy or breast feeding
8. Harmful alcohol consumption as evaluated by the Investigator
9. Flare in colitis activity within last 90 days requiring intensification of therapy beyond baseline maintenance treatment; use of oral prednisolone >10 mg/day, biologics (i.e. monoclonal antibodies) and or hospitalisation for colitis within 90 days. Prior use of biologics is not a contraindication to screening
10. Diagnosed cholangiocarcinoma or high clinical suspicion of cholangiocarcinoma either clinically or by imaging
11. Concurrent malignancies or invasive cancers diagnosed within past 3 years except for adequately treated basal cell and squamous cell carcinoma of the skin and in situ carcinoma of the uterine cervix
12. Presence of a percutaneous drain or bile duct stent
13. Major surgical procedure within 30 days of screening
14. Prior organ transplantation
15. Known hypersensitivity to the investigational product or any of its formulation excipients
16. Unavailable for follow-up assessment or concern for subject's compliance
17. Participation in an investigational trial of a drug or device within 60 days of screening or 5 half-lives of the last dose of investigational drug, where the trial drug half-life is greater than 12 days
18. Any other condition that in the opinion of the Investigator renders the subject a poor risk for inclusion into the trial
19. Positive screening test for tuberculosis (TB) (including T-SPOT.TB TB test), unless respiratory review confirms false positive test results
20. Receipt of live vaccination within 6 weeks prior to Screening Visit 2
21. Known HIV positive status

## 5. CONSENT AND SCREENING

### 5.1 Informed Consent

It is the responsibility of the Principal Investigator (PI) or Co-investigators (detailed in the site delegation log) to obtain written informed consent for each patient prior to performing any trial related procedure. A Patient Information Sheet (PIS) is provided to facilitate this process. Investigators must ensure that they adequately explain the aim, trial treatment, anticipated benefits and potential hazards of taking part in the trial to the patient. The investigator should also stress that the patient is completely free to refuse to take part or withdraw from the trial at any time without any detrimental effect on their standard care. The patient should be given ample time (e.g. 24 hrs) to read the PIS and to discuss their participation with others outside of the site research team. The patient must be given an opportunity to ask questions which should be answered to their satisfaction. The right of the patient to refuse to participate in the trial without giving a reason must be respected.

If the patient expresses an interest in participating in the trial they should be asked to sign and date the latest version of the Informed Consent Form (ICF). The PI or Co-investigator (detailed in the site delegation log) must then sign and date the form. A copy of the ICF should be given to the patient, a copy should be filed in the hospital notes, and the original placed in the Investigator Site File (ISF). Once the patient is entered into the trial the patient's trial number should be entered on the ICF maintained in the ISF. In addition, if the patient has given explicit consent a copy of the signed Informed Consent Form must be sent in the post to the Trials Office for review.

Details of the informed consent discussions should be recorded in the patient's medical notes, this should include date of, and information regarding, the initial discussion, the date consent was given, with the name of the trial and the version number of the PIS and ICF. Throughout the trial the patient should have the opportunity to ask questions about the trial and any new information that may be relevant to the patient's continued participation should be shared with them in a timely manner. On occasion it may be necessary to re-consent the patient in which case the process above should be followed and the patient's right to withdraw from the trial respected.

Electronic copies of the PIS and ICF are available from the BUTEO Trials Office and should be printed or photocopied onto the headed paper of the local institution.

Details of all patients approached about the trial should be recorded on the Patient Screening/Enrolment Log and with the patient's prior consent their General Practitioner (GP) should also be informed that they are taking part in the trial. A GP Letter is provided electronically for this purpose.

### 5.2 Screening

Prior to formal screening process patients will be required to provide written informed consent **BEFORE** any non-standard of care assessment can be performed, as detailed in section 5.1. It is anticipated that the majority of the screening process (except MRI imaging) will be completed at the same visit (i.e. all blood tests performed on the same day).

Each patient who has consented, and enters the screening process, will be allocated a screening number. Each site will be provided with a Patient Screening Enrolment log, which is pre-populated with site specific screening numbers. Please use this number in any correspondence with the BUTEO

Trial Office. If the patient is eligible for the trial, the screening number should be completed on the Registration Form. A Trial Number will be allocated once the patient is registered.

The screening process consists of two visits which are detailed below:

**Screening Visit 1 (within 5-7 weeks of Visit 3):**

- Vital signs (heart rate, blood pressure, temperature, respiratory rate)
- Physical exam to be performed by clinician
- 12-lead electrocardiogram (ECG)
- Bloods for Full Blood Count (FBC), International Normalised Ratio (INR), Urea and Electrolytes (UEs), Liver Function Tests (LFTs)
- Blood sample (whole blood and serum) for biomarker and research blood analysis
- Medical history including any current symptoms any pre-existing conditions
- Record of patient concomitant medication (only record con-meds that are currently being taken by the patient)
- Evaluation of inclusion and exclusion criteria
- MRI scan (to be performed between consent and registration)
- TB screening - T-SPOT.TB test (see Appendix 3)
- Pregnancy test (for females patients of childbearing potential only)

**Screening Visit 2 (within 1 week of Visit 3):**

- Bloods for FBC, INR, UEs, LFTs
- Blood sample (whole blood and serum) for biomarker and research blood analysis
- Record of patient concomitant medication
- Patient medical history (including current symptom and any conditions since Visit 1)
- Pregnancy test (for females patients of childbearing potential only)
- Mayo PSC Risk Score
- MELD score
- Fibroscan
- Give IBD Diary (if applicable)
- Evaluation of the inclusion/exclusion criteria

***If the repeat ALP measurement: is within +/- 25% of previous sample (Visit 1) and the patient continues to be eligible for the trial, proceed to patient registration (see Section 6 of the trial protocol for procedure)***

# 6. TRIAL ENTRY

## 6.1 Pre-registration

After the results of the screening process (Visits 1 and 2) are available, the following will be verified by the local site PI and/or Co-Investigators before registering the patient onto the trial:

- Patient's ICF
- Confirmation of all the inclusion criteria
- Review of all the exclusion criteria
- Patient's Screening Number
- Completion of Registration Form

All the above must be completed before proceeding onto patient registration.

## 6.2 Patient Registration

All patients must be registered with the CRCTU at the University of Birmingham and their Screening Number provided during the registration process.

### 6.2.1 Patient Registration During Dose Confirmation Phase

During the dose confirmation phase of the clinical trial all participating centres are required to contact the Trial Office directly **before** approaching any potential patient. This is important to confirm whether a slot is available with the BUTEO Trial Office. In addition during this phase of the trial the site office will be in contact on a weekly basis with sites, when any cohort is near to completion. This will ensure that patients are only approached by trial sites, if there is a likelihood of them being recruited into the trial. Site investigators are also required to keep the BUTEO office informed of any circumstances (patient refusal, screen failure, previously recruited patients fail to reach Visit 7 [Day 50] etc.) that may mean that previously allocated slots become available.

All patients that are eligible for inclusion in the trial and have signed an ICF will be registered via the BUTEO Trials Office. Informed consent must be obtained prior to registration. An Eligibility Checklist and Registration Form must be completed and sent to the BUTEO Trial Office.

The patient's eligibility will be confirmed at registration by the BUTEO Trial Office. If eligible for the trial, the patient will be allocated a unique Trial Number. Trial drug prescriptions must include the patient's Trial Number and dose. The registration line will be open during office hours (09:00 – 17:00, Monday-Friday).

| |
|---|
| Registration line: ☎ 0121 371 8158 or 0121 371 8157<br>🖷 0121 371 7246<br>(09:00 – 17:00, Monday-Friday) |

At this time point, patients will be allocated a unique trial number to preserve patient confidentiality. The schedule for investigations and follow up visits is summarised in section 7.3.

### 6.2.2 Patient Registration During Dose Expansion Phase (Via Electronic Remote Data Capture System)

**All sites will be informed and provided with full instructions when the online electronic Remote Data Capture system (eRDC) has been implemented.**

All patients that are eligible for inclusion in the trial and have signed an ICF will be registered via the online electronic remote data capture system (eRDC). Informed consent must be obtained prior to registration. An Eligibility Checklist must also be completed and faxed to the Trials Office before the online Registration Form is completed.

A copy of the patient's ICF must be sent in the post to the BUTEO Trial Office, if the patient has given explicit consent for this.

In the expansion phase of the trial, registration of patients can be achieved by logging on to:

https://www.cancertrials.bham.ac.uk

Full details will be provided to sites when this is in use.

At this time point, patients will be allocated a unique trial number to preserve patient confidentiality. The schedule for investigations and follow up visits is summarised in Section 7.3.

**NB. Once the eRDC system has been implemented, patient's will NOT be registered via the method in section 6.2.1.**

## 6.3 Emergency Registration

Once the eRDC has been implemented, in case of any problems with online registration, a paper eligibility checklist and registration form should be completed. These details should then be phoned through to the BUTEO Trial Office at CRCTU, University of Birmingham using the below numbers:

**Contact details for emergency registration**

Emergency Registration line: ☎ 0121 371 8158 or 0121 371 8157

🖷 0121 371 7246

(09:00 – 17:00, Monday-Friday)

After an emergency registration, patients will be given a unique trial number which will be allocated in sequence by the CRCTU as detailed above.

# 7. TREATMENT DETAILS

## 7.1 Investigational Medicinal Product Preparation

The Investigational Medicinal Product (IMP) is BTT1023. This is a fully-human IgG4 immunoglobulin with antigen-binding specificity for human VAP-1. It consists of two κ light chains and two heavy chains with a total molecular mass of approximately 146 kDa. BTT1023 is produced in a Chinese hamster ovary cell culture and purified with appropriate methods including specific viral inactivation and removal procedures. Additional information is contained within the BTT1023 Investigator Brochure.

### 7.1.1 Dosage Form and Composition

The dosage form of BTT1023 is concentrate for solution for infusion (see latest version of Biotie Therapies Corp. Investigator's Brochure BTT12-CD011-S04 BTT1023 for further information).
BTT1023 is formulated at 20 mg/ml in a buffer containing 20 mM sodium citrate, 100 mM sodium chloride, 1.5% D-mannitol, 20 µM diethylenetriaminepentaacetic acid and 0.02% polysorbate 80 in water for injection; the pH is 7.0 ± 0.5.

BTT1023 is contained in single-use 10 mL clear type I glass vials with a white-coloured cap. Each vial is filled to the nominal volume of 5.0 mL of the sterile concentrate solution, thus each vial contains a nominal total of 100.0 mg of BTT1023.

BTT1023 requires dilution prior to administration by infusion. The amount of diluent to be added to the BTT1023 concentrate is calculated to consistently provide a total diluted drug product infusion volume of 50 ml. The amount of diluent required is based on the calculated dosage for each patient according to instructions provided to the administering clinicians.

The diluent product is designated as BTT1023 IV Infusion Diluent, 10 ml. It contains 0.9% sodium chloride and 0.02% polysorbate 80 in water for injection; the osmolality is 310 ± 30 mOsmol/kg, within the range for typical physiological value. It is contained in single-use 20 mL clear type I glass vials with a blue-coloured cap. Each vial is filled to the nominal volume of 10.0 mL of the sterile diluent solution.

### 7.1.2 Instructions for Storage

BTT1023 and its diluent are to be stored at 2-8°C.

### 7.1.3 Instructions for Handling the Investigational Medicinal Product

Since no preservative is present, it is recommended that the administration of the diluted solution for infusion is started as soon as possible after dilution. Due to a risk of microbial contamination the maximum shelf life for the diluted infusion solution is 24 hrs when stored at 2 to 8°C.

A 0.5-1 mL aliquot of the BTT1023 infusion solution will be taken at the end of the infusion period and stored at 2-8°C prior to dispatch to the bioanalytical laboratory. These aliquots will be labelled with the trial number, patient number and date of intravenous infusion. The samples will be stored at the bioanalytical laboratory (Envigo) and may be used for analysis of BTT1023 concentration if anomalies in PK data are observed that could be due to errors in IMP preparation. The samples will be discarded after the Clinical Trial Report has been signed.

Further IMP details can be found in the current version of the BUTEO Pharmacy Manual.

## 7.2 Trial Treatment

This clinical trial is a single-arm clinical trial therefore all patients registered on to the trial will receive the same treatment as detailed below in Table 3.

**Treatment group:** patients will receive multiple doses of BTT1023 (maximum of 7 doses) at 8, 12 or 16 mg/kg body weight.

**Table 3:** BTT1023 Drug Infusion Table

| Visit number | Infusion number | Dose | Infusion time * |
|---|---|---|---|
| **3** | **1** | **8, 12 or 16 mg/kg** | 120 minutes |
| **4-9** | **2-7** | **8, 12 or 16 mg/kg** | 60 minutes |

* Infusion rate for subsequent visits will only be increased if the individual patient did not experience a break in infusion due to AEs in the previous visit.

Patients will only receive one-dose level and no dose reductions will be allowed. If a patient experiences any events that require; either a SINGLE infusion to be interrupted, or result in an incomplete dose being given at any drug infusion visit then the patient will be assessed and the treating clinician will determine continuation of therapy based on an evaluation of the clinical reasons for interruption to therapy. If appropriate after discussion with the clinical coordinator and/or chief investigator a joint decision will be made if the patient should remain on the trial and receive additional infusions at protocol defined scheduled visits. This decision will be fully documented in the individual patient medical record and site files as appropriate.

## 7.3 Treatment Schedule (Visit 3 – Visit 9)

**Treatment Visit 3 (Day 1)**

Pre-medication cetirizine 10 mg, ibuprofen 400 mg orally (in the absence of any contra-indication to non-steroidal anti-inflammatory drug [NSAIDs]) 1-2 hrs pre-infusion, and 100 mg hydrocortisone approximately 20-30 minutes pre-infusion

- Vital signs (heart rate, blood pressure, temperature, respiratory rate) pre-infusion
- Physical exam to be performed by clinician
- 12 lead electrocardiogram (ECG) to be performed pre-infusion and 60 minutes post end of drug infusionClinical assessment to document clinical events and AEs
- Record of patient concomitant medication
- Quality of Life Questionnaires- EQ-5D, Fatigue Severity Scale, Pruritus Visual Analogue Score (VAS), collect IBD Diaries (if applicable) **(pre infusion)**
- BTT1023 infusion
- Vital signs (heart rate, blood pressure, temperature, respiratory rate) repeated at 30 and 60 minutes after start of infusion, and 30, 60, 90, 120, 180, 240 minutes post end of drug infusion
- Bloods for full blood count (FBC), international normalised ratio (INR), urea and electrolytes (UEs), liver function tests (LFTs), pre-infusion. See Table 4
- Blood samples (whole blood and serum) for biomarker and research blood analysis pre-infusion
- Bloods for FBC, INR, UE, LFT 2hrs post end of infusion period. See Table 4

- Blood serum for (PK (pre-infusion and then 15, 30, 60, 90 minutes post infusion start, end of infusion, 180, 360 minutes post infusion start. Some patients at the Birmingham site may have a 24 hr (post end of infusion) PK if they agree to this
- Blood serum for ADA (pre-infusion)
- Blood serum for PD (pre-infusion and then 180 and 360 minutes post infusion start). Some patients at the Birmingham site may have a 24 hr (post end of infusion) PD if they agree to this

**Treatment Visit 4 (Day 8 +/- 3 days)**

Pre-medication cetirizine 10 mg, ibuprofen 400 mg orally (in the absence of any contraindication to NSAIDs) 1-2 hrs pre-infusion and 100 mg hydrocortisone approximately 20-30 minutes pre-infusion (See Section 7.9)

- Vital signs (heart rate, blood pressure, temperature, respiratory rate) pre-infusion
- 12 lead electrocardiogram (ECG) to be performed pre-infusion and 60 minutes post end of drug infusion Clinical assessment to document clinical events and AEs
- Record of patient concomitant medication
- Bloods for FBC, INR, UEs, LFTs pre-infusion. See Table 4
- Blood samples (whole blood and serum) for biomarker and research blood analysis pre-infusion
- Blood serum for PK (pre-infusion, end of infusion and then 60, 120 and 180 minutes **post end of drug infusion**)
- Blood serum for PD (pre-infusion)
- BTT1023 infusion
- Vital signs (heart rate, blood pressure, temperature, respiratory rate) repeated at 30 and 60 minutes after start of infusion, and 30, 60,120,180 minutes post end of drug infusion
- Bloods for FBC, INR, UEs, LFTs 2hrs post end of infusion period. See Table 4

**Treatment Visit 5 (Day 22+/- 3 days)**

Pre-medication cetirizine 10 mg, ibuprofen 400 mg orally (in the absence of any contra-indication to NSAIDs) 1-2 hrs pre infusion and 100 mg hydrocortisone approximately 20-30 minutes pre-infusion (See Section 7.9)

- Pregnancy test (for females patients of childbearing potential only)
- Vital signs (heart rate, blood pressure, temperature, respiratory rate) pre-infusion
- Physical exam to be performed by clinician
- 12 lead electrocardiogram (ECG) to be performed pre-infusion and 60 minutes post end of drug infusion Clinical assessment to document clinical events and AEs
- Record of patient concomitant medication
- Blood samples (whole blood and serum) for biomarker and research blood analysis pre-infusion
- Bloods for FBC, INR, UEs, LFTs pre-infusion. See Table 4
- BTT1023 infusion
- Vital signs (heart rate, blood pressure, temperature, respiratory rate) repeated at 30 and 60 minutes after start of infusion, and 30, 90, 120 minutes post end of drug infusion
- Bloods for FBC, INR, UEs, LFTs 2hrs post end of infusion period. See Table 4
- Blood serum for PK (pre-infusion, end of infusion and 60 minutes post end of drug infusion)

- Blood serum for PD (pre-infusion)

**Treatment Visit 6 (Day 36 +/- 3 days)**

Pre-medication cetirizine 10 mg + ibuprofen 400 mg orally (in the absence of any contra-indication to NSAIDs) 1-2 hrs pre infusion (See Section 7.9). 100 mg hydrocortisone can be administered at the clinician's discretion.

- Vital signs (heart rate, blood pressure, temperature, respiratory rate) pre-infusion
- 12 lead electrocardiogram (ECG) to be performed pre-infusion and 60 minutes post end of drug infusion
- Clinical assessment to document clinical events and AEs
- Record of patient concomitant medication
- Bloods for FBC, INR, UEs, LFTs pre-infusion. See Table 4
- Blood samples (whole blood and serum) for biomarker and research blood analysis pre-infusion
- Bloods for FBC, INR, UEs, LFTs 2hrs post end of infusion period. See Table 4
- Blood serum for PK (pre-infusion, end of infusion and 60 minutes post end of drug infusion)
- Blood serum for PD (pre-infusion)
- BTT1023 infusion
- Vital signs (heart rate, blood pressure, temperature, respiratory rate) repeated at 30 and 60 minutes after start of infusion, and 30, 90, 120 minutes post end of drug infusion
- Quality of Life Questionnaires- EQ-5D, Fatigue Severity Scale, Pruritus Visual Analogue Score (VAS), IBD Diaries (if applicable)

**Treatment Visit 7 (Day 50+/- 3 days)**

Pre-medication cetirizine 10 mg + ibuprofen 400 mg orally (in the absence of any contra-indication to NSAIDs) 1-2 hrs pre infusion (See Section 7.9). 100 mg hydrocortisone can be administered at the clinician's discretion.

- Pregnancy test (for females patients of childbearing potential only)
- Vital signs (heart rate, blood pressure, temperature, respiratory rate) pre-infusion
- Physical exam to be performed by clinician
- 12 lead electrocardiogram (ECG) to be performed pre-infusion and 60 minutes post end of drug infusion Clinical assessment to document clinical events and AEs
- Record of patient concomitant medication
- Bloods for FBC, INR, UEs, LFTs pre-infusion. See Table 4
- Blood samples (whole blood and serum) for biomarker and research blood analysis pre-infusion
- Blood serum for PK (pre-infusion, end of infusion and 60 minutes post end of drug infusion)
- Blood serum for PD (pre-infusion)
- Blood serum for ADA (pre-infusion)
- BTT1023 infusion
- Vital signs (heart rate, blood pressure, temperature, respiratory rate) repeated at 30 and 60 minutes after start of infusion, and 30, 90, 120 minutes post end of drug infusion
- Bloods for FBC, INR, UEs, LFTs 2hrs post end of infusion period. See Table 4

**Treatment Visit 8 (Day 64 +/- 3 days)**

Pre-medication cetirizine 10 mg + ibuprofen 400 mg orally (in the absence of any contra-indication to NSAIDs) 1-2 hrs pre infusion (See Section 7.9). 100 mg hydrocortisone can be administered at the clinician's discretion.

- Vital signs (heart rate, blood pressure, temperature, respiratory rate) pre-infusion
- 12 lead electrocardiogram (ECG) to be performed pre-infusion and 60 minutes post end of drug infusion
- Clinical assessment to document clinical events and AEs
- Record of patient concomitant medication
- Bloods for FBC, INR, UEs, LFTs pre-infusion. See Table 4
- Blood samples (whole blood and serum) for biomarker and research blood analysis pre-infusion
- Blood serum for PK (pre-infusion, end of infusion and 60 minutes post end of drug infusion)
- Blood serum for PD (pre-infusion)
- BTT1023 infusion
- Vital signs (heart rate, blood pressure, temperature, respiratory rate) repeated at 30 and 60 minutes after start of infusion, and 30, 90, 120 minutes post end of drug infusion
- Bloods for FBC, INR, UEs, LFTs 2 hrs post end of infusion period. See Table 4

**Treatment Visit 9 (Day 78 +/- 3 days)**

Pre-medication cetirizine 10 mg + ibuprofen 400 mg orally (in the absence of any contra-indication to NSAIDs) 1-2 hrs pre infusion (See Section 7.9). 100 mg hydrocortisone can be administered at the clinician's discretion.

- Pregnancy test (for females patients of childbearing potential only)
- Vital signs (heart rate, blood pressure, temperature, respiratory rate) pre-infusion
- Physical exam to be performed by clinician
- 12 lead electrocardiogram (ECG) to be performed pre-infusion and 60 minutes post end of drug infusion
- Clinical assessment to document clinical events and AEs
- Record of patient concomitant medication
- Bloods for FBC, INR, UEs, LFTs pre-infusion. See Table 4
- Blood samples (whole blood and serum) for biomarker and research blood analysis pre-infusion
- Blood serum for PK (pre-infusion and then 15, 30 minutes post infusion start, end of infusion, and 120 minutes post infusion start). Some patients at the Birmingham site may have a 24 hr PK if they agree to this
- Blood serum for ADA (pre-infusion)
- Blood serum for PD (pre-infusion and then 120 minutes post infusion start). Some patients at the Birmingham site may have a 24 hr (post end of drug infusion) PD if they agree to this
- BTT1023 infusion
- Vital signs (heart rate, blood pressure, temperature, respiratory rate) repeated at 30 and 60 minutes after start of infusion, and 30, 90, 120 minutes post end of drug infusion
- Bloods for FBC, INR, UEs, LFTs 2 hrs post end of infusion period. See Table 4

## 7.4 Trial Schema

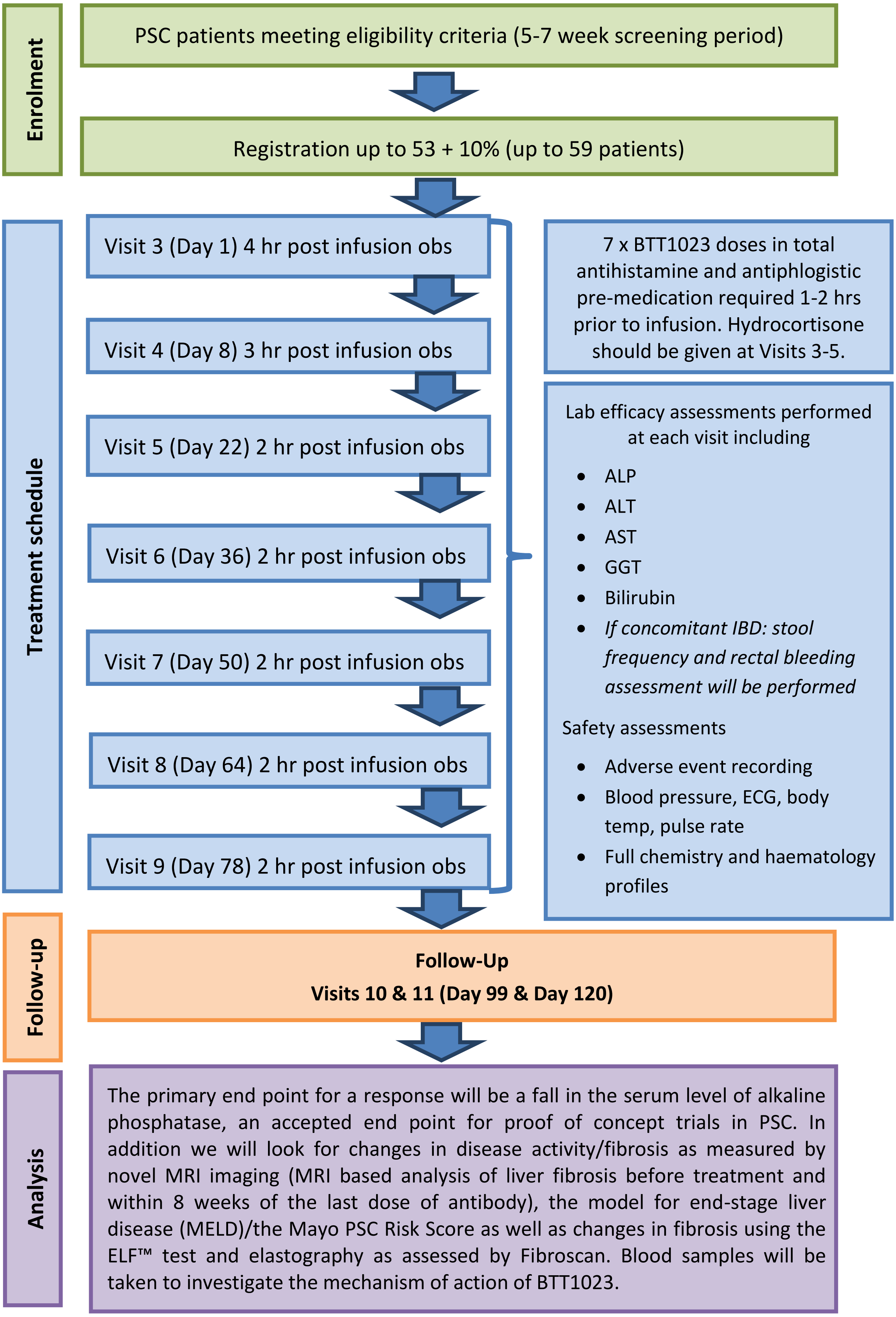


**Figure 2:** Trial/Patient Flow Diagram

## 7.5 Assessments

### 7.5.1 Blood Chemistry and Haematology

The following blood tests will be performed at each visit. Tests used for activity assessment will be performed in a central laboratory, whereas safety laboratory assessments will be done in local laboratories at investigative sites.

**Table 4:** Blood Tests

| | Blood test | Primary reason for assessment |
|---|---|---|
| **Biochemistry** | Alkaline Phosphatase (ALP) - central analysis | Activity |
| | Alkaline Phosphatase (ALP) - local analysis | Safety |
| | Alanine Transaminase (ALT) | |
| | Aspartate Transaminase (AST) | |
| | Albumin | |
| | Bilirubin (direct and indirect if total is ≥ 30) | |
| | Gamma Glutamyl Transferase (GGT) | |
| | Sodium | Safety |
| | Potassium | |
| | Urea | |
| | Creatinine | |
| | Calcium | |
| | Total Protein | |
| | *Estimated Glomerular Filtration Rate (eGFR) | |
| **Haematology** | Haemoglobin | Safety |
| | Platelets | |
| | Red Blood Cells | |
| | White Blood Cells | |
| | Haematocrit | |
| | Mean Cell Volume | |
| | Mean Cell Haemoglobin | |
| | Neutrophils | |
| | Lymphocytes | |
| | Monocytes | |
| | Eosinophils | |
| | Basophils | |
| | INR | |
| | APTT Ratio | |

* calculated by Cockcroft-Gault equation or alternative as per institutional practice – Appendix 1

**ONLY IN THE EVENT OF SUSPECTED CYTOKINE RELEASE SYNDROME**: The following blood tests will need to be performed to confirm adverse event. These tests are considered as part of standard care when investigating this specific syndrome. Please see the Laboratory Manual for further details.

**Table 5:** Immunology Blood Tests

| | | |
|---|---|---|
| **Immunology** | TNF- α | Safety |
| | IFN-ϒ | |
| | IL-6 | |

### 7.5.2 Pharmacokinetics Scheduling

**Table 6:** Visit 3+9 Pharmacokinetics Scheduling

Visit 3 (Day 1) + Visit 9 (Day 78 +/- 3 days)

| **Time point** | **Blood sample type** |
|---|---|
| Pre-infusion | Blood serum |
| 15 minutes post infusion start | Blood serum |
| 30 minutes post infusion start | Blood serum |
| 60 minutes post infusion start* | Blood serum |
| 90 minutes post infusion start* | Blood serum |
| End of infusion | Blood serum |
| 180 minutes** (120 minutes) post infusion start | Blood serum |
| 360 minutes** post infusion start | Blood serum |
| 24 hrs post end of infusion time # | Blood serum |

# will only be performed on a sub-set of patients (treated at the Queen Elizabeth Hospital Birmingham) and only if the individual patients agree.

*due to the length of the infusion (60 minutes) at infusion Visit 9 this time point will not be required.

**at infusion Visit 9 patients only remain for a maximum of 2 hrs post infusion so these PK time points will be ended at 120 minutes on the day of infusion.

**Table 7:** Visit 4 Pharmacokinetics Scheduling

Visit 4 (Day 8 +/- 3 days)

| **Time point** | **Blood sample type** |
|---|---|
| Pre-infusion | Blood serum |
| End of infusion | Blood serum |
| 60 minutes post end of infusion | Blood serum |
| 120 minutes post end of infusion | Blood serum |
| 180 minutes post end of infusion | Blood serum |

**Table 8:** Visit 5-8 Pharmacokinetic Scheduling

Visit 5 (Day 22 +/- 3 days), Visit 6 (Day 36 +/- 3 days), Visit7 (Day 50 +/- 3 days), Visit 8 (Day 64 +/- 3 days)

| **Time point** | **Blood sample type** |
|---|---|
| Pre-infusion | Blood serum |
| End of infusion | Blood serum |
| 60 minutes post end of infusion | Blood serum |

### 7.5.3 Physical Examination/Symptom Assessment

Physical assessments will be performed by the trial clinician and include height (to be performed at Visit 1 only) and weight. A physical examination (including general appearance, respiratory, cardiovascular, skin, head and neck, lymph nodes, thyroid, abdomen, musculo-skeletal) should be performed at Screening Visit 1, prior to infusions during Visits 3, 5, 7 and 9 and during follow up Visits 10 and 11.

### 7.5.4 Mayo Risk Assessment

The patient's age, bilirubin, albumin, AST and history of variceal bleeding will be used to calculate the Mayo PSC Risk score at Visit 2 and Visit 10 [Day 99]. The following link can be used:

http://www.mayoclinic.org/medical-professionals/model-end-stage-liver-disease/revised-natural-history-model-for-primary-sclerosing-chonalgitis

## 7.6 Sample Collection

### 7.6.1 Blood Samples

The following samples will be collected during the trial:

1. Standard blood tests - FBC, INR, UEs, LFTs
2. Blood and serum sample for biomarker and research blood analysis
3. Blood serum for PK
4. Blood serum for ADA
5. Blood serum for PD (sVAP-1)
6. Blood for T-SPOT.TB test *[screening visit 1 only]*

### 7.6.2 Pharmacokinetic, Anti-Drug Antibodies and Pharmacodynamic Samples

For all patients, blood samples for the determination of BTT1023, ADA and sVAP-1 will be collected as is scheduled in the Schedule of Events table. Please see the latest version of the laboratory manual for further information.

## 7.7 Dose Modifications

No patient dose modifications or dose reductions will be allowed during the dose confirmation phase of the trial. In the phase II component of the trial, dose reduction or modifications may be considered at the discretion of the Chief Investigator. Please contact the BUTEO Trials Office to discuss further. The dose should not be reduced or modified until written permission from the Chief Investigator has been received.

## 7.8 Treatment Compliance

The local hospital will keep drug accountability records that can be reviewed by sponsor representatives.

Please refer to the current BUTEO Pharmacy Manual for further details.

## 7.9 Pre-Medication

The pre-medication is cetirizine 10 mg + ibuprofen 400 mg orally (in the absence of any contra-indication to NSAIDs) about 1-2 hrs before the start of every infusion of BTT1023. Additionally, all patients should be given 100 mg hydrocortisone approximately 20-30 minutes prior to the infusions at Visits 3-5. Hydrocortisone can be administered at subsequent infusions at the clinicians' discretion. In case of intolerance/allergy to hydrocortisone or Cetirizine, please contact the BUTEO Trials office as confirmation with the Clinical Coordinator or Medical monitor prior to infusion is required. If there is a delay in administering the BTT1023 infusion consider whether this is delay is sufficient enough to warrant further pre-medication.

## 7.10 Concomitant Medication

All medication that each participant is taking at the time, or within 3 months, of enrolment will be recorded. New medications or changes to current medications during the trial will also be recorded.

The pharmaceutical/trade name, dose, route of administration, indication, start/stop date of each new medication within the trial will be recorded. Any drug that is licensed within the United Kingdom and Europe, that is deemed necessary for the participant's health-care, will be permitted at the discretion of the Chief Investigator.

Prohibited medications include:

- Biological drugs (i.e. monoclonal antibodies) other than the investigational product
- Initiation during the trial period of any therapy that has the potential to change serum alkaline phosphatase, e.g. high dose steroids, methotrexate, fenofibrate, ursodeoxycholic acid (UDCA) or related agents
- In those patients receiving UDCA in compliance with the inclusion criteria, changes in UDCA dosage are not permitted during the trial period
- Live vaccinations in the six weeks prior to Baseline Visit 2 (this is an inclusion criteria) through to six weeks after the last BTT1023 infusion

## 7.11 Contraception and Pregnancy

All sexually active women of childbearing potential must agree to use a highly effective method of contraception from the Screening Visit throughout the trial period and for 99 days following the last dose of trial drug. If using hormonal agents the same method must have been used for at least 1 month before trial dosing and subjects must use a barrier method as the other form of contraception. Lactating women must agree to discontinue breast feeding before trial investigational medicinal product administration.

For the purposes of this trial, a female subject of childbearing potential is a woman who has not had a hysterectomy, bilateral oophorectomy, or medically-documented ovarian failure. Women ≤ 50 years of age with amenorrhea of any duration will be considered to be of childbearing potential.

Men, if not vasectomised, must agree to use barrier contraception (condom plus spermicide) during heterosexual intercourse from screening through to trial completion and for 99 days from the last dose of trial investigational medicinal product.

**Refer to section 8 if a pregnancy is reported, either that of a female participant, or the partner of a male participant.**

## 7.12 Patient Follow Up

**Visit 10*: End of Treatment Visit (Day 99 +/- 3 days)**

- Vital signs (heart rate, blood pressure, temperature, respiratory rate)
- Physical exam (to be performed by clinician)
- Clinical assessment to document clinical events and AEs
- Record of patient concomitant medication
- Bloods for FBC, INR, UEs, LFTs
- Blood serum (PK, ADA and PD)
- Blood sample (whole blood and serum) for biomarker and research blood analysis
- Fibroscan
- ECG
- Quality of Life Questionnaires- EQ-5D, Fatigue Severity Scale, Pruritus Visual Analogue Score (VAS), IBD Diaries (if applicable)

**Visit 11: End of Treatment Visit (Day 120 +/- 3 days)**

- Vital signs (heart rate, blood pressure, temperature, respiratory rate)
- Physical exam (to be performed by clinician)
- Clinical assessment to document clinical events and adverse events
- Record of patient concomitant medication. Bloods for FBC, INR, UEs, LFTs
- Blood serum (PK, ADA and PD)
- Blood sample (whole blood and serum) for biomarker and research blood analysis
- MRI within 4-6 weeks after the last treatment (Visit 9) or last dose of antibody*

* If a patients stops treatment early this visit will be 21 days (+/-3 days) after the last dose of BTT11023 with the MRI within 8 weeks of last dose of BTT11023

NB: All visit dates / time are related to Visit 3 (Day 1). If a visit is delayed for any reason the next visit date will be scheduled in relation to Visit 3. There are no planned treatment delays. If a drug infusion visit cannot be performed within the required timeframe, every attempt should be made to administer the infusion as close as possible to the permitted time window. In extreme situations, protocol-scheduled visits may be cancelled and declared as missed visits at the clinical discretion of the Investigator.

## 7.13 Patient Withdrawal

The Investigator will make every reasonable effort to keep each patient on treatment. However, if the Investigator removes a patient from the trial treatment or if (s)he declines further participation

final assessments will be performed, if possible. All the results of the evaluations and observations, together with a description of the reasons for trial withdrawal, must be recorded in the CRF.

In the event of a patient's decision to withdraw from the trial, the Investigator must ascertain from which aspects of the trial the patient wishes to withdraw, and record the details on the appropriate CRF. All patients will continue to be followed-up (if they agree), and all information and tissue samples collected up until the point of retraction, will be retained and analysed.

Patients who are removed from treatment due to adverse events (clinical or laboratory) will be treated and followed according to accepted medical practice. All pertinent information concerning the outcome of such treatment must be recorded in the CRF.

The following are justifiable reasons for the Investigator to withdraw a patient from trial:

- Unacceptable toxicity
- Unforeseen events - any event which in the judgement of the Investigator makes further treatment inadvisable
- SAE requiring discontinuation of treatment
- Withdrawal of consent
- Serious violation of the trial protocol (including persistent patient attendance failure and persistent non-compliance)
- Withdrawal by the Investigator for clinical reasons not related to the trial drug treatment

# 8. ADVERSE EVENT REPORTING

The collection and reporting of AEs will be in accordance with the Medicines for Human Use Clinical Trials Regulations 2004 and its subsequent amendments. Definitions of different types of AE are listed in Appendix 4. The Investigator should assess the seriousness and causality (relatedness) of all AEs experienced by the patient (this should be documented in the source data) with reference to the current BTT1023 Investigator Brochure.

## 8.1 Reporting Requirements

All AEs, whether serious or not, will be recorded throughout the trial. At each contact with the site, the patient will be asked about AEs. All AEs, either observed by the Investigator or reported by the subject, will be recorded by the Investigator and evaluated.

Collection, Recording and Reporting of Adverse Events:

In line with the Medicines for Human Use (Clinical Trials) Regulations 2004, an accurate and up to date record of all AEs reported by investigators will be maintained throughout the trial. This record will include details of nature, onset, duration, severity, outcome and any relationship to the investigational product. The Sponsor, appropriate regulatory authority (e.g. Medicines and Healthcare Products Regulatory Agency (MHRA)) and Research Ethics Committee will be informed as required by current regulations.

The NCI Common Terminology Criteria for Adverse Events (CTCAE) Version 4.0 will be used to grade each AE. In the event of the specific AE term not being available in the CTCAE the associated body system 'AE term –other' will be used and the event graded in accordance with guidelines i.e.: Grade 1: mild, Grade 2: Moderate, Grade 3: severe, Grade 4: life-threatening and grade 5: Death. As determined by the treating clinician.

A non-leading, open question will be asked initially to evaluate for possible AEs. Examples include:

At screening (Visit 1-2) - 'Are you experiencing any symptoms?'

At subsequent visits (Visits 3 – 11) – 'How have you been since your last visit?'

As a minimum requirement, all Serious Adverse Events (SAEs) will be reported to Biotie Therapies and their parent company, Acorda. Events such as medication errors and suspected transmission of an infectious agent via a trial product will always be considered as medical events of special interest and will be reported to Biotie Therapies and Acorda irrespective of seriousness. These events will need to be reported as an SAE.

**Pre-existing conditions**

Any pre-existing conditions should be reported as medical history, and should not be reported as an AE unless the condition worsens by at least one CTC grade during the trial. The condition, however, must be reported in the CRF.

### 8.1.1 Adverse Events

All medical occurrences which meet the definition of an AE (see Appendix 4 for definition) should be reported. Please note this includes all abnormal laboratory findings, including those that are not clinically significant.

### 8.1.2 Hypersensitivity, Infusion Reactions and Infusion Related Reactions

#### 8.1.2.1 Hypersensitivity and Infusion Reactions

Hypersensitivity reactions are defined according to the NCI-CTCAE Version 4.0 definition of allergic reaction / hypersensitivity, as follows:

Grade 1: transient flushing or rash, drug fever <38°C

Grade 2: rash, flushing, urticaria, dyspnoea, drug fever ≥38°C

Grade 3: symptomatic bronchospasm, requiring parenteral medication(s), with or without urticaria; allergy-related oedema/angioedema, hypotension

Grade 4: anaphylaxis (a life-threatening event characterized by the rapid onset [often within minutes] of airway obstruction [bronchospasm, stridor, hoarseness], urticaria, and/or hypotension)

#### 8.1.2.2 Infusion Related Reactions

Infusion-related reactions are defined according to the NCI-CTCAE Version 4.0 definition, as follows:

Grade 1: mild, transient reaction, infusion interruption not indicated; intervention not indicated

Grade 2: therapy or infusion interruption indicated but responds promptly to symptomatic treatment (for example, antihistamines, non-steroidal anti-inflammatory drugs [NSAIDS], narcotics, I.V. fluids); prophylactic medications indicated for ≤24 hrs

Grade 3: Prolonged (for example, not rapidly responsive to symptomatic medication and/or brief interruption of infusion); recurrence of symptoms following initial improvement; hospitalisation indicated for clinical sequelae

Grade 4: Life-threatening consequences; urgent intervention indicated consistent with usual medical practice, selected parenteral medications may be utilised as detailed below. Additional treatments, chosen according to clinical symptoms and local standards, may be utilised at investigator discretion.

### 8.1.3 Serious Adverse Events

Investigators should report AEs that meet the definition of an SAE (see Appendix 4 for definition).

#### 8.1.3.1 Monitoring Pregnancies for Potential Serious Adverse Events

It is important to monitor the outcome of pregnancies of patients in order to provide SAE data on congenital anomalies or birth defects.

In the event that a patient or their partner becomes pregnant during the SAE reporting period please complete a Pregnancy Notification Form (providing the patient's details) and return to the BUTEO Trials Office as soon as possible. If it is the patient who is pregnant provide outcome data on a follow-up Pregnancy Notification Form. Where the patient's partner is pregnant consent must first be obtained and the patient should be given a Pregnancy Release of Information Form to give to their partner. If the partner is happy to provide information on the outcome of their pregnancy they should sign the Pregnancy Release of Information Form. Once consent has been obtained provide details of the outcome of the pregnancy on a follow-up Pregnancy Notification Form. If appropriate also complete an SAE Form as detailed below.

### 8.1.4 Reporting Period

The reporting period for AEs will commence from the date of consent, (typically Visit 1: screening visit) and will continue until the follow-up visit ( Visit 11: Day 120), or alternatively up to 45 days post last trial drug infusion if the patient withdraws from the trial prior to completion of all 7 trial drug infusions.

The reporting period for SAE will commence from the date of consent, (typically Visit 1: screening visit) and will continue until the follow-up visit (Visit 11: Day 120), or alternatively up to 45 days post last trial drug infusion if the patient withdraws from the trial prior to completion of all 7 trial drug infusions.

SAEs that are judged to be at least possibly related to the IMP must still be reported in an expedited manner irrespective of how long after IMP administration the reaction occurred.

## 8.2 Reporting Procedure

### 8.2.1 Site

#### 8.2.1.1 Adverse Events

AEs should be reported on an AE Form (and where applicable on an SAE Form). AEs should be completed at each visit and the information recorded on the case report form (eRDC) /or paper form as appropriate.

In the event of the specific AE term not being available in the CTCAE the associated body system 'AE term –other' will be used and the event graded in accordance with guidelines i.e.: Grade 1: mild, Grade 2: Moderate, Grade 3: severe, Grade 4: life-threatening and grade 5: Death. As determined by the treating clinician.

#### 8.2.1.2 Serious Adverse Events

For more detailed instructions on SAE reporting refer to the SAE Form Completion Guidelines section of the ISF.

AEs defined as serious and which require reporting as an SAE should be reported on an SAE Form. When completing the form, the Investigator will be asked to define the causality and the severity of the AE which should be documented using the CTCAE version 4.0.

On becoming aware that a patient has experienced an SAE, the Investigator (or delegate) must complete, date and sign an SAE Form. The form should be faxed together with a SAE Fax Cover Sheet to the Trials Office using one of the numbers listed below as soon as possible and no later than 24 hrs after first becoming aware of the event:

To report an SAE, fax the SAE Form with an SAE Fax Cover Sheet to:

**0121 371 7246 (primary number)**

**0121 414 7989 (secondary number)**

On receipt the BUTEO Trials Office will allocate each SAE a unique reference number. This number will be transcribed onto the SAE Fax Cover Sheet which will then be faxed back to the site as proof of receipt. If confirmation of receipt is not received within 1 working day please contact the Trials Office. The SAE reference number should be quoted on all correspondence and follow-up reports regarding the SAE. The SAE Fax Cover Sheet completed by the BUTEO Trials Office should be filed with the SAE Form in the ISF.

For SAE Forms completed by someone other than the Investigator, the Investigator will be required to countersign the original SAE Form to confirm agreement with the causality and severity assessments. The form should then be returned to the BUTEO Trials Office in the post and a copy kept in the ISF.

Investigators should also report SAEs to their own Trust in accordance with local practice.

#### 8.2.1.3 Provision of Follow-up Information

Patients should be followed up until resolution or stabilisation of the event. Follow-up information should be provided on a new SAE Form (refer to the SAE Form Completion Guidelines for further information).

### 8.2.2 Trials Office

On receipt of an SAE Form seriousness and causality will be determined independently by a Clinical Coordinator. An SAE judged by the Investigator or Clinical Coordinator to have a reasonable causal relationship with the trial medication will be regarded as a Serious Adverse Reaction (SAR). The Clinical Coordinator will also assess all SARs for expectedness. If the event meets the definition of a SAR that is unexpected (i.e. is not defined in the Investigator Brochure) it will be classified as a Suspected Unexpected Serious Adverse Reaction (SUSAR).

### 8.2.3 Reporting to the Competent Authority and Research Ethics Committee

#### 8.2.3.1 Suspected Unexpected Serious Adverse Reactions

The BUTEO Trials Office will report a minimal data set of all individual events categorised as a fatal or life threatening SUSAR to the Medicines and Healthcare products Regulatory Agency (MHRA) and

Research Ethics Committee (REC) within 7 days. Detailed follow-up information will be provided within an additional 8 days.

All other events categorised as SUSARs will be reported within 15 days.

Concurrently with the above, the trials office will report all such events to Biotie Therapies (and Acorda) nominated safety representative (IMP provider).

#### 8.2.3.2 Serious Adverse Reactions

The BUTEO Trials Office will report details of all SARs (including SUSARs) to the MHRA and REC annually from the date of the Clinical Trial Authorisation, in the form of a Developmental Safety Update Report Adverse Events.

Details of all AEs will be reported to the MHRA on request.

#### 8.2.3.3 Other Safety Issues Identified During the Course of the Trial

The MHRA and REC will be notified immediately if a significant safety issue is identified during the course of the trial.

### 8.2.4 Investigators

Details of all SUSARs and any other safety issue which arises during the course of the trial will be reported to Principal Investigators. A copy of any such correspondence should be filed in the ISF.

### 8.2.5 Data Monitoring Committee

The independent Data Monitoring Committee (DMC) will review all SAEs.

### 8.2.6 Provider of Investigational Medicinal Product

All SAEs will be reported to the provider (Biotie Therapies and Acorda) of the Investigational Medicinal Product (BTT1023) within 24 hrs by email.

# 9. DATA HANDLING AND RECORD KEEPING

## 9.1 Data Collection

Data will be collected during this clinical trial. Data will be captured on case report forms (CRF), initially this will be on paper forms. Once the electronic remote data capture (eRDC) systems is implemented sites will be informed and further details provided. The Case Report Form (paper CRF/eRDC) is listed in Table 9 below.

**Table 9:** Description of Case Report Forms

| Form | Summary of data recorded | Schedule for submission |
|---|---|---|
| Eligibility Checklist | Confirmation of eligibility and satisfactory staging investigations where necessary | Faxed at point of registration. |
| Registration | Patient details and Registration Number | Faxed at point of registration. |
| **Dosing Forms** | | |
| BTT1023 Dosing | Dose level, Visit, Date, Dosing details | Within 2 weeks |
| **Sample Analysis Forms** | | |
| Pharmacokinetics | Date, Visit, Sample collection, time taken | Within 2 weeks |
| Pharmacodynamics | Date, Visit, Sample collection, time taken | Within 2 weeks |
| Biomarker Sample | Date, Visit, Sample collection, time taken | Within 2 weeks |
| ADA | Date, Visit, Sample collection, time taken | Within 2 weeks |
| Research Sample | Date, Visit, Sample collection, time taken | Within 2 weeks |
| **Assessment Forms** | | |
| Adverse Event | Start and stop dates, grade according to CTCAE version 4.0, causality | Every 4 weeks and as requested |
| Concomitant Medication | List of concomitant medication | Every 4 weeks and as requested |
| Biochemistry (Screening, Pre Dose & Post Dose Treatment, Follow Up) | Date, Visit, results, clinical significance | Within 2 weeks |
| ECG | Date, Visit, results | Within 2 weeks |
| Haematology | Date, Visit, results, clinical significance | Within 2 weeks |
| Medical History | Prior conditions, diagnosis, grade | Within 2 weeks |

| | | |
|---|---|---|
| Previous Treatment | Previous Treatment start/ end date, treatment type, dose and schedule if applicable | Within 2 weeks |
| Ethnicity | Date, Visit, Ethnicity | Within 2 weeks |
| Physical Examination | Date, Visit, investigations | Within 2 weeks |
| Pregnancy Test | Date, Visit, result | Within 2 weeks |
| Vital Signs | Date, Visit, pre- post dose time, results | Within 2 weeks |
| Mayo Risk Score | Date, Visit, results required for Mayo Risk Score Calculation, Mayo Risk Score | Within 2 weeks |
| MRI | Date, Visit, Results | Within 2 weeks |
| MELD Score | Date, Visit, Results | Within 2 weeks |
| T-Spot TB Test | Date, Visit, Results | Within 2 weeks |
| Fibroscan | Date, Visit, Results | Within 2 weeks |
| **Ad Hoc Forms** | | |
| Treatment Discontinuation | As required | Within 4 weeks of trial visit date |
| Serious Adverse Event Form | Details of the SAE | Within 24 hrs of being made aware of the SAE |
| Pregnancy Notification Form | Patient details and details of pregnancy | Within 24 hrs of being made aware of the pregnancy |
| Death Form | Date and cause of death | Immediately upon notification of death |
| Deviation Form | Completed in the event of a deviation from the protocol | Immediately upon discovering deviation. |
| Withdrawal Form | Used to notify the Trials Office of patient withdrawal from the trial | Immediately upon patient withdrawal |
| Quality of Life Forms | Patient response to QOL questions | Originals within 4 weeks of trial visit date. Copies to be kept in ISF. |
| IBD Diaries (*if applicable)* | Patient response to IBD Diary questions | Originals within 4 weeks of trial visit date. Copies to be kept in ISF |

The CRF must be completed, signed/dated (for paper CRFs only) and returned to the BUTEO Trials Office by the Investigator or an authorised member of the site research team (as delegated on the Site Signature and Delegation Log) within the timeframe listed above entries on the CRF should be made in ballpoint pen, in blue or black ink, and must be legible. Any errors should be crossed out

with a single stroke, the correction inserted and the change initialled and dated. If it is not obvious why a change has been made, an explanation should be written next to the change.

Data reported on each form should be consistent with the source data or the discrepancies should be explained. If information is not known, this must be clearly indicated on the form. All missing and ambiguous data will be queried. All sections are to be completed before returning.

The MELD Score CRF/ eRDC page should be completed with the Creatinine, bilirubin and INR results only. The MELD Score will be calculated by the BUTEO Trial Office using the pre-2016 MELD Score calculation (https://www.mdcalc.com/meld-score-original-pre-2016-model-end-stage-liver-disease).

Data provided on the Quality of Life Forms will not be captured in the source data or eRDC. The originals should be sent to the BUTEO Trial Office, and a copy filed within in the ISF.

In all cases it remains the responsibility of the Investigator to ensure that the CRF has been completed correctly and that the data are accurate.

The completed originals should be sent to the BUTEO Trials Office and a copy filed in the ISF.

Trial forms may be amended by the BUTEO Trials Office, as appropriate, throughout the duration of the trial. Whilst this will not constitute a protocol amendment, new versions of the form must be implemented by participating sites immediately on receipt.

### 9.2 Archiving

It is the responsibility of the Principal Investigator to ensure all essential trial documentation and source records (e.g. signed ICFs, ISFs, Pharmacy Files, patients' hospital notes, copies of CRFs etc.) at their site are securely retained for at least 15 years after the end of the trial or following the processing of all biological material collected for research, whichever is the later. Do not destroy any documents without prior approval from the CRCTU Document Storage Manager.

## 10. QUALITY MANAGEMENT

The BUTEO clinical trial in patients with PSC is being conducted under the auspices of the Cancer Research UK Clinical Trials Unit (CRCTU) according to the current guidelines for Good Clinical Practice (GCP). Participating sites will be monitored by CRCTU staff to confirm compliance with the protocol and the protection of patients' rights as detailed in the Declaration of Helsinki: October 1996 (Appendix 5).

### 10.1 Site Set-up and Initiation

All sites will be required to sign a Clinical Trial Site Agreement prior to participation. In addition all participating Investigators will be asked to sign the necessary agreements, Investigator Registration Forms and supply a current CV and GCP to the BUTEO Trials Office. All members of the site research team will also be required to sign the Site Signature and Delegation Log, which should be returned to the BUTEO Trials Office. Prior to commencing recruitment all sites will undergo a process of initiation. Key members of the site research team will be required to attend either a meeting or a teleconference covering aspects of the trial design, protocol procedures, AE reporting, collection and reporting of data and record keeping. Sites will be provided with an ISF and a Pharmacy File containing essential documentation, instructions, and other documentation required for the conduct of the trial. The BUTEO Trials Office must be informed immediately of any change in the site research team.

## 10.2 On-site Monitoring

Monitoring will be carried out as required following a risk assessment and as documented in the BUTEO Quality Management Plan. Additional on-site monitoring visits may be triggered for example by poor CRF return, poor data quality, low SAE reporting rates, excessive number of patient withdrawals or deviations. If a monitoring visit is required the Trials Office will contact the site to arrange a date for the proposed visit and will provide the site with written confirmation. Investigators will allow the BUTEO trial staff access to source documents as requested.

## 10.3 Central Monitoring

Trials staff will be in regular contact with the site research team to check on progress and address any queries that they may have. Trials staff will check incoming CRFs for compliance with the protocol, data consistency, missing data and timing. Sites will be sent Data Clarification Forms (DCFs) requesting missing data or clarification of inconsistencies or discrepancies.

Sites may be suspended from further recruitment in the event of serious and persistent non-compliance with the protocol and/or GCP, and/or poor recruitment. Any major problems identified during monitoring may be reported to the Trial Management Group and the Trial Steering Committee and the relevant regulatory bodies. This includes reporting serious breaches of GCP and/or the trial protocol to the main Research Ethics Committee (REC) and the Medicines for Healthcare products Regulatory Agency (MHRA).

## 10.4 Audit and Inspection

The Investigator will permit trial-related monitoring, audits, ethical review, and regulatory inspection(s) at their site, providing direct access to source data/documents.

Sites are also requested to notify the BUTEO Trials Office of any MHRA inspections.

## 10.5 Notification of Serious Breaches

In accordance with Regulation 29A of the Medicines for Human Use (Clinical Trials) Regulations 2004 and its amendments, the Sponsor of the trial is responsible for notifying the licensing authority in writing of any serious breach of:

- The conditions and principles of GCP in connection with that trial or;
- The protocol relating to that trial, within 7 days of becoming aware of that breach

For the purposes of this regulation, a “serious breach” is a breach which is likely to effect to a significant degree:

- The safety or physical or mental integrity of the subjects of the trial; or
- The scientific value of the trial

Sites are therefore requested to notify the Trials Office of a suspected trial-related serious breach of GCP and/or the trial protocol. Where the Trials Office is investigating whether or not a serious breach has occurred sites are also requested to cooperate with the Trials Office in providing sufficient information to report the breach to the MHRA where required and in undertaking any corrective and/or preventive action.

## 11. END OF TRIAL DEFINITION

The end of trial will be the date of Last Patient Last Visit (LPLV). The BUTEO Trials Office will notify the MHRA and REC that the trial has ended and will provide them with a summary of the clinical trial report within 12 months of the end of trial.

After closure of the trial with the MHRA, the Sponsor is no longer required to notify the MHRA and REC of changes of Principal Investigator. However, sites should continue to notify the BUTEO Trials Office of changes in Principal Investigator by completing and returning (where required) an Investigator Registration Form together with a current signed and dated CV.

The trial may close early if stage-1 continuation criterion (detailed in Section 12) is not met.

## 12. STATISTICAL CONSIDERATIONS

No specific toxicities are expected with the BTT1023 treatment however, a Dose Limiting Toxicity is defined as an AE that meets the criteria of grade 4 or grade 5 as defined in the Common terminology criteria for adverse events (CTCAE) (Appendix 2). However, if a definite diagnosis of cytokine release syndrome is made, it will be classed as a DLT at grade 3 and above* and must be considered at least possibly related to BTT1023.

If the trough levels of circulating BTT1023 are found to be <3 µg/ml, this indicates that a therapeutic level has not been reached and either the dose will escalate or the trial will stop. Details of the possible dose-pathways have been described in Section 3.

### 12.1 Definition of Outcome Measures

#### 12.1.1 Primary Outcome Measure

Response at Day 99: a reduction in serum ALP by 25% from baseline to Day 99. This is a binary indicator and is based on the change being -25% (a negative value indicates a reduction). The calculation method and formula can be found in the Statistical Analysis Plan.

#### 12.1.2 Secondary Outcome Measures

Secondary outcome measures listed in Section 2.2 will be analysed with regard to safety and tolerability, improvement from baseline to Day 99 and evaluation of changes in sVAP-1/SSAO as a biomarker of liver disease. Analysis methods for the secondary outcome measures will be detailed further in the Statistical Analysis Plan.

### 12.2 Analysis of Outcome Measures

This single arm Simon's design (Simon, R., 1989) includes an interim assessment of the accumulating data. The first assessment (stage-1) takes place once 18 patients have been evaluated for the primary outcome (response). If 3 or more responses are observed in stage-1 then the trial will continue into stage-2. Recruitment will not be halted while stage-1 is assessed. If the stage-1 criterion is not met then the trial will cease.

A further 19 patients will be recruited during stage-2. The treatment will be accepted as active if 9 or more objective responses are observed out of the total 37 patients recruited. As before, given uncertainty around patient dropout, a set of possible scenarios and the respective stop/go criteria and associated error rates are provided below. The final success criterion chosen maintains power,

and in doing so can incur an increased type-1 error rates (note that the stage-1 criterion is fixed since the final sample size is unknown at stage-1).

**Table 10:** Possible Stop/Go Guidelines and Associated Error Rates

| Patients (stage-1) | Responses (stage-1) | Patients (total) | Responses (total) | Type-1 error ($\alpha$) | Power (1- $\beta$) |
|---|---|---|---|---|---|
| 18 | 3 | 34 | 8 | 0.120 | 0.826 |
| 18 | 3 | 35 | 8 | 0.134 | 0.846 |
| 18 | 3 | 36 | 8 | 0.149 | 0.863 |
| **18** | **3** | **37** | **9** | **0.087** | **0.806** |
| 18 | 3 | 38 | 9 | 0.099 | 0.827 |
| 18 | 3 | 39 | 9 | 0.111 | 0.846 |
| 18 | 3 | 40 | 9 | 0.124 | 0.861 |
| 18 | 3 | 41 | 10 | 0.073 | 0.809 |

Not all of the alternative stop/go criteria detailed above are published in the referenced paper. These have been calculated by the trial Biostatistician and independently verified by a CRCTU Biostatistician.

Any patient without a Visit 10 (Day 99) ALP measurement, e.g. due to withdrawal, lost to follow-up or attendance failure, will be treated as a non-responder in the primary outcome analysis. Extreme values will be queried by the trials management team, and if present at the final analysis stage may be excluded as part on a sensitivity analysis.

Deviation(s) from the original statistical plan will be documented in version controlled updates to the statistical analysis plan.

### 12.3 Planned Sub Group Analyses

No subgroup analyses are planned.

### 12.4 Planned Interim Analysis

Stopping guidelines detailed in section 12.2.

### 12.5 Planned Final Analyses

Final analyses of the primary outcome will be performed when all patients have been followed to Day 99 and once the database has locked. Stopping guidelines detailed in section 12.2.

### 12.6 Power Calculations

The Phase II, single arm trial follows a Simon's 2-stage minimax design (Simon, R., 1989) with lower and upper acceptability bounds of 15 and 30% respectively and error rates $\alpha$=0.10 and $\beta$=0.20. Thirty-seven patients are required, however, to account for patient dropout, estimated at approximately 10%, the sample size is extended by a further 4 patients. As such the total target recruitment is 41 patients recruited from 6 UK centres over a three to four year period.

In this setting the interpretation of $\alpha$ is the probability satisfying stage-1 and observing 9 or more responses in 37 patients overall when the true response rate is 15%; a false positive result (Type I error). Beta ($\beta$) is the probability of failing to acknowledge activity when the true response rate is

30% (Type II error). As such the power, 1- β, is the probability of taking an effective treatment forward.

# 13. TRIAL ORGANISATIONAL STRUCTURE

## 13.1 Sponsor

The trial sponsor is the University of Birmingham.

## 13.2 Coordinating Centre

The trial is being conducted under the auspices of the Cancer Research UK Clinical Trials Unit (CRCTU), University of Birmingham according to their local procedures.

## 13.3 Trial Management Group

The Trial Management Group (TMG) is comprised of the Chief Investigator and other collaborators as detailed elsewhere in this document. The TMG will be responsible for the day-to-day running and management of the trial and will meet by teleconference or in person as required. Members of the TMG include the CI, CRCTU Trial Management Team Leader, Senior Trial Coordinator, Trial Coordinator, Lead Trial Statistician, Trial Statistician and a Pharmacy Representative. The TMG will meet or hold a teleconference every 2 months during recruitment, or as required.

## 13.4 Trial Steering Committee

A Trial Steering Committee (TSC) will provide overall supervision and representation for both the funders and sponsor of the trial, whilst providing advice through its independent chair. Membership includes members of the TMG, CRCTU Trial Management Team Leader, and selected Principal Investigators. Other members/observers may be invited upon request. The ultimate decision for the continuation of the trial lies with the TSC. The TSC will meet at least once a year or more often if required.

## 13.5 Data Monitoring Committee

Data analyses will be supplied in confidence to an independent DMC (DMC), which will be asked to give advice on whether the accumulated data from the trial, together with the results from other relevant research, justifies the continuing recruitment of further patients. The DMC will operate in accordance with a trial specific charter based upon the template created by the Damocles Group.

During the run-in period results will be provided to the DMC and discussed via teleconference at a minimum. The DMC will meet after the first 6 patients have completed treatment and the DLT reporting timeline has been completed for these 6 patients. This will be to review the run in phase of the trial. The DMC may halt recruitment if this DLT is unacceptable. After this phase, the DMC will be scheduled to meet one month prior to the due date of the Annual Safety Report and annually thereafter.

During the recruitment phase of the Simon's 2-stage component the DMC will meet at the end of stage 1, after 18 evaluable patients have been assessed for the primary outcome (ALP response at Day 99) and yearly thereafter. A meeting will be arranged to coincide with the reporting of Stage-1 stop/go guidelines (see Section 12).

Additional meetings may be called if recruitment is much slower/ faster than anticipated and the DMC may, at their discretion, request to meet more frequently or continue to meet following completion of recruitment. An emergency meeting may also be convened if a safety issue is

identified. The DMC will report directly to the Trial Management Group (TMG) who will convey the findings of the DMC to the Trial Steering Committee and funders. The DMC may consider recommending the discontinuation of the trial if the recruitment rate or data quality are unacceptable or if any issues are identified which may compromise patient safety.

The DMC will report directly to the BUTEO Trial Management Group (TMG) who will convey (when applicable) the findings of the DMC to the sponsors, Biotie Therapies, MHRA and the Ethics Committee. The DMC have the right to recommend closure of the trial if any issues are identified which may compromise patient safety.

### 13.6 Finance

This is a clinician-initiated and clinician-led trial funded by the National Institute for Health Research (NIHR) with receives funds directly from the Department of Health (DoH). Biotie Therapies will be supplying BTT1023, the investigational medicinal product (IMP), free of charge to all individual NHS trusts that will be directly treating patients as part of this clinical trial.

***Payments to individual NHS trusts (Per-patient payment)***

Refer to the Clinical Trial Site Agreement for details.

## 14. ETHICAL CONSIDERATIONS

The trial will be performed in accordance with the recommendations guiding physicians in biomedical research involving human subjects, adopted by the 18$^{th}$ World Medical Association General Assembly, Helsinki, Finland, June 1964, amended at the 48$^{th}$ World Medical Association General Assembly, Somerset West, Republic of South Africa, October 1996 (website: http://www.wma.net/en/30publications/10policies/b3/index.html).

The trial will be conducted in accordance with the Research Governance Framework for Health and Social Care, the applicable UK Statutory Instruments, (which include the Medicines for Human Use Clinical Trials 2004 and subsequent amendments and the General Data Protection Regulation 2018 and Data Protection Act 2018 for health and care research) and the Principles of Good Clinical Practice (GCP). This trial will be carried out under a Clinical Trial Authorisation in accordance with the Medicines for Human Use Clinical Trials regulations. The protocol will be submitted to and approved by the main Research Ethics Committee (REC) prior to circulation.

Before any patients are enrolled into the trial, the Principal Investigator at each site is required to obtain local R&D approval. Sites will not be permitted to enrol patients until written confirmation of R&D approval is received by the Trials Office.

It is the responsibility of the Principal Investigator to ensure that all subsequent amendments gain the necessary local approval. This does not affect the individual clinicians' responsibility to take immediate action if thought necessary to protect the health and interest of individual patients.

## 15. CONFIDENTIALITY AND DATA PROTECTION

Personal data recorded on all documents will be regarded as strictly confidential and will be handled and stored in accordance with the General Data Protection Regulation 2018 and Data Protection Act 2018 for health and care research. The University of Birmingham, as the Sponsor for the BUTEO trial, will be using information from patient medical records in order to undertake this trial and will act as

the data controller for the trial. This means that the University of Birmingham are responsible for looking after the information and using it properly. University of Birmingham and the NHS will keep identifiable information about the patients for at least 25 years after the trial has finished, to allow the results of the trial to be verified if needed.

All information collected by the Sponsor will be securely stored at the Trials Office at the University of Birmingham on paper and electronically and will only be accessible by authorised personnel. The only people in the University of Birmingham who will have access to information that identifies a patient will be people who manage the trial or audit the data collection process.

The NHS will use the patient's name and contact details to contact patients about the research trial, and make sure that relevant information about the trial is recorded for their care, and to oversee the quality of the trial. With the patient's permission, the research team (at site) will notify the patient's GP that they intend to participate in the trial.

With the patient's consent, their full name (on consent form only), initials, date of birth and hospital number, will be collected at trial entry. The research team (at site) will send a copy of the signed consent form in the post or by fax to the Trials Office. This will be used to perform in-house monitoring of the consent process.

In routine communication between the hospital and the Trials Office, the patient will only be identified by trial number and date of birth. Data may be provided to the Trials Office on paper or electronically.

By taking part in the trial, the patient will be agreeing to allow research staff from the Trials Office at the University of Birmingham to look at the trial records, including their medical records. It may be necessary to allow authorised personnel from government regulatory agencies (e.g. Medicines and Healthcare products Regulatory Agency (MHRA), the Sponsor and/or NHS bodies to have access to patient medical and research records. This is to ensure that the trial is being conducted to the highest possible standards. Anonymised data from the trial may also be provided to other third parties (e.g. the manufacturer of the trial treatment) for research, safety monitoring or licensing purposes.

The Investigator must maintain documents not for submission to the Trials Office (e.g. Patient Identification Logs) in strict confidence. In the case of specific issues and/or queries from the regulatory authorities, it will be necessary to have access to the complete trial records, provided that patient confidentiality is protected.

## 16. INSURANCE AND INDEMNITY

University of Birmingham employees are indemnified by the University insurers for negligent harm caused by the design or co-ordination of the clinical trials they undertake whilst in the University's employment.

In terms of liability at a site, NHS Trust and non-Trust hospitals have a duty to care for patients treated, whether or not the patient is taking part in a clinical trial. Compensation is therefore available via NHS indemnity in the event of clinical negligence having been proven.

The University of Birmingham cannot offer indemnity for non-negligent harm. The University of Birmingham is independent of any pharmaceutical company, and as such it is not covered by the Association of the British Pharmaceutical Industry guideline's for patient compensation.

## 17. PUBLICATION POLICY

Results of this trial will be submitted for publication in a peer reviewed journal. The manuscript will be prepared by the Trial Management Group (TMG) and authorship will be determined by mutual agreement.

Any secondary publications and presentations prepared by Investigators must be reviewed by the TMG. Manuscripts must be submitted to the TMG in a timely fashion and in advance of being submitted for publication, to allow time for review and resolution of any outstanding issues. Authors must acknowledge that the trial was performed with the support of the University of Birmingham. Intellectual property rights will be addressed in the Clinical Trial Site Agreement between Sponsor and site.

## 18. REFERENCE LIST


Biotie Therapies Corp., (2008). BTT-1023 anti-VAP-1 monoclonal antibody toxicity study by intravenous (bolus) administration to cynomolgus monkeys for 14 weeks followed by an 8 week recovery period.

Biotie Therapies Corp., (2010). BTT12-CD015: A multiple ascending dose study to assess the safety and pharmacokinetics of repeated intravenous doses of BTT1023 in patients with rheumatoid arthritis - a double-blind randomized placebo-controlled sequential group trial.

Hirschfield, G. M., T. H. Karlsen, K. D. Lindor and D. H. Adams (2013). "Primary sclerosing cholangitis." The Lancet **382**(9904): 1587-1599.

Jalkanen, S. and M. Salmi (2001). "Cell surface monoamine oxidases: enzymes in search of a function." The EMBO Journal **20**(15): 3893-3901.

Kurkijärvi, R., D. H. Adams, R. Leino, T. Möttönen, S. Jalkanen and M. Salmi (1998). "Circulating Form of Human Vascular Adhesion Protein-1 (VAP-1): Increased Serum Levels in Inflammatory Liver Diseases." The Journal of Immunology **161**(3): 1549-1557.

Kurkijärvi, R., G. G. Yegutkin, B. K. Gunson, S. Jalkanen, M. Salmi and D. H. Adams (2000). "Circulating soluble vascular adhesion protein 1 accounts for the increased serum monoamine oxidase activity in chronic liver disease." Gastroenterology **119**(4): 1096-1103.

Lalor, P. F., S. Edwards, G. McNab, M. Salmi, S. Jalkanen and D. H. Adams (2002). "Vascular Adhesion Protein-1 Mediates Adhesion and Transmigration of Lymphocytes on Human Hepatic Endothelial Cells." The Journal of Immunology **169**(2): 983-992.

Liaskou, E., M. Karikoski, G. M. Reynolds, P. F. Lalor, C. J. Weston, N. Pullen, M. Salmi, S. Jalkanen and D. H. Adams (2011). "Regulation of mucosal addressin cell adhesion molecule 1 expression in human and mice by vascular adhesion protein 1 amine oxidase activity." Hepatology **53**(2): 661-672.

Simon, R (1989). "Optimal Two-Stage Designs for Phase II Clinical Trials". Controlled Clinical Trials **10**:1-10.

Weston, C. J., E. L. Shepherd, L. C. Claridge, P. Rantakari, S. M. Curbishley, J. W. Tomlinson, S. G. Hubscher, G. M. Reynolds, K. Aalto, Q. M. Anstee, S. Jalkanen, M. Salmi, D. J. Smith, C. P. Day and D. H. Adams (2015). "Vascular adhesion protein-1 promotes liver inflammation and drives hepatic fibrosis." The Journal of Clinical Investigation **125**(2): 501-520.

## APPENDIX 1 – EQUATION FOR ESTIMATED GLOMERULAR FILTRATION RATE

### COCKCROFT-GAULT

$$\text{Creatinine clearance (estimated GFR)} = \frac{[(140 - \text{age in years}) \times (\text{wt in kg})] \times 1.23}{\text{serum creatinine in micromol/l}}$$

For women multiply the result of calculation by 0.85.

## APPENDIX 2 - COMMON TOXICITY CRITERIA GRADINGS

Toxicities will be recorded according to the Common Terminology Criteria for Adverse Events (CTCAE), version 4.0. The full CTCAE document is available on the National Cancer Institute (NCI) website, the following address was correct when this version of the protocol was approved:

http://ctep.cancer.gov/protocolDevelopment/electronic_applications/ctc.htm

## APPENDIX 3 – T-SPOT.TB

**Oxford Diagnostics Laboratories**

**SERVICE DESCRIPTION**

Oxford Diagnostic Laboratories (ODL®) offers a national TB testing service to laboratories and clinicians using the T-SPOT®.*TB* test.

Further information on the T-SPOT.*TB* test, including its limitations, is set out in our published documentation.

**Specimen Acceptance Criteria**

We require 6ml of blood collected in one standard heparin (green-topped) vacutainer tube. Paediatric samples require less blood (please call the laboratory to seek advice, as blood volumes depend on age). Samples must be accompanied with a completed ODL test Request Form and must reach ODL within 32 hrs of venepuncture. Blood samples should be stored at 18-25°C; do not refrigerate samples. Please ensure that you include your Customer Account Number and Quote/Agreement Number on all test requests. Specimens must arrive by 2.00pm Monday to Saturday. ODL is closed on public holidays and over the Christmas and New Year period each year (please call us, or consult our website, to find out the exact dates for this year).

We place no restriction on the numbers of samples that can be sent at one time, however, if sending large numbers of samples we would appreciate advance notification to help us better plan our workflow. We endeavour to provide results two working days after the sample is received by ODL.

**What will Oxford Diagnostic Laboratories do?**

1. We will carry out the Service, as described here and in the Terms and Conditions of Service, and following the instructions given on the test Request Form.

2. The service results will be sent by email (and fax or post if requested) to the requesting laboratory within 2 working days of receipt of sample. For incidents, results are reported within three working days.

3. The invoice for the Service will be sent by post within 3 further working days, unless otherwise agreed.

4. ODL will be responsible for disposal of any samples provided.

**Results Interpretation and Quality Control**

A typical result would be expected to have few or no spots in the Nil Control and _20 spots in the Positive Control. Each spot represents the footprint of an individual cytokine-secreting T cell, and evaluating the number of spots obtained provides a measurement of the abundance of *M. tuberculosis* complex sensitive effector T cells in the peripheral blood.

A Nil Control spot count in excess of 10 spots should be considered as 'Indeterminate'. Refer to the T-SPOT.*TB* Technical Handbook for possible causes (download from www.oxfordimmunotec.com). If this occurs, another sample should be collected from the individual and sent to ODL for testing.

Typically, the cell functionality Positive Control spot count should be _ 20 or show saturation (where spots are too numerous to count).

A small proportion of patients may have T cells which show only a limited response to PHA. Where the Positive Control spot count is< 20 spots, it should be considered as 'Indeterminate', unless either Panel A or Panel B is 'Positive' as described below, in which case the result is valid.

The T-SPOT.*TB* test results are interpreted by subtracting the spot count in the Nil Control well from the spot count in each of the Panel wells, according to the following algorithm:

- The test result is 'Positive' if (Panel A minus Nil Control) and / or (Panel B minus Nil Control) _ 6 spots.
- The test result is 'Negative' if both (Panel A minus Nil Control) and (Panel B minus Nil Control) _ 5 spots. This includes values less than zero.

Due to potential biological and systematic variations, where the higher of (Panel A minus Nil Control) and (Panel B minus Nil Control) is5, 6 or 7 spots, the result may be considered as Borderline (equivocal). Borderline (equivocal) results, although valid, are less reliable than results where the spot count is further from the cut-off. Retesting of the patient, using a new sample, is therefore recommended.

**A 'Positive' result indicates that Tuberculosis infection is likely.**

**A 'Negative' result indicates that Tuberculosis infection is unlikely.**

The results should be used and interpreted only in the context of the overall clinical picture. A negative test result does not exclude the possibility of exposure to or infection with *M. tuberculosis.*

## APPENDIX 4 - DEFINITION OF ADVERSE EVENTS

**Adverse Event**

Any untoward medical occurrence in a patient or clinical trial subject administered a medicinal product and which does not necessarily have a causal relationship with this treatment.

Comment:

An AE can therefore be any unfavourable and unintended sign (including abnormal laboratory findings), symptom or disease temporally associated with the use of an investigational medicinal product, whether or not related to the investigational medicinal product.

**Adverse Reaction**

All untoward and unintended responses to an IMP related to any dose administered.

Comment:

An AE judged by either the reporting Investigator or Sponsor as having causal relationship to the IMP qualifies as an AR. The expression reasonable causal relationship means to convey in general that there is evidence or argument to suggest a causal relationship.

**Serious Adverse Event**

Any untoward medical occurrence or effect that at any dose:

- Results in death
- Is life-threatening*
- Requires hospitalisation** or prolongation of existing inpatients' hospitalisation
- Results in persistent or significant disability or incapacity
- Is a congenital anomaly/birth defect
- Or is otherwise considered medically significant by the Investigator***
- Any suspected or definite diagnosis of cytokine release syndrome grade 3 – 5. *In order to confirm the case of cytokine release syndrome, an additional blood sample will need to be taken for cytokine assay (e.g. TNF-α, IFNγ, IL-6). This information is to be provided as part of the SAE form.*

Comments:

The term severe is often used to describe the intensity (severity) of a specific event. This is not the same as serious, which is based on patients/event outcome or action criteria.

* Life threatening in the definition of an SAE refers to an event in which the patient was at risk of death at the time of the event; it does not refer to an event that hypothetically might have caused death if it were more severe.

**Hospitalisation is defined as an unplanned, formal inpatient admission, even if the hospitalisation is a precautionary measure for continued observation. Thus hospitalisation for protocol treatment (e.g. line insertion), elective procedures (unless brought forward because of worsening symptoms) or for social reasons (e.g. respite care) are not regarded as an SAE.

*** Medical judgment should be exercised in deciding whether an AE is serious in other situations. Important AEs that are not immediately life threatening or do not result in death or hospitalisation

but may jeopardise the subject or may require intervention to prevent one of the other outcomes listed in the definition above, should be considered serious.

**Serious Adverse Reaction**

An Adverse Reaction which also meets the definition of a Serious Adverse Event.

**Suspected Unexpected Serious Adverse Reaction**

A SAR that is unexpected i.e. the nature, or severity of the event is not consistent with the applicable product information.

A SUSAR should meet the definition of an AR, UAR and SAR.

**Unexpected Adverse Reaction**

An AR, the nature or severity of which is not consistent with the applicable product information (e.g. Investigator Brochure for an unapproved IMP or (compendium of) Summary of Product Characteristics (SPC) for a licensed product).

When the outcome of an AR is not consistent with the applicable product information the AR should be considered unexpected.

## APPENDIX 5 - WMA DECLARATION OF HELSINKI

**WORLD MEDICAL ASSOCIATION DECLARATION OF HELSINKI**

**Recommendations guiding physicians in biomedical research involving human subjects**

Adopted by the 18th World Medical Assembly Helsinki, Finland, June 1964and amended by the29th World Medical Assembly, Tokyo, Japan, October 1975, 35th World Medical Assembly, Venice, Italy, October 1983, 41st World Medical Assembly, Hong Kong, September 1989 and the 48th General Assembly, Somerset West, Republic of South Africa, October 1996

**INTRODUCTION**

It is the mission of the physician to safeguard the health of the people. His or her knowledge and conscience are dedicated to the fulfilment of this mission.

The Declaration of Geneva of the World Medical Association binds the physician with the words, "The Health of my patient will be my first consideration," and the International Code of Medical Ethics declares that, "A physician shall act only in the patient's interest when providing medical care which might have the effect of weakening the physical and mental condition of the patient."

The purpose of biomedical research involving human subjects must be to improve diagnostic, therapeutic and prophylactic procedures and the understanding of the aetiology and pathogenesis of disease.

In current medical practice most diagnostic, therapeutic or prophylactic procedures involve hazards. This applies especially to biomedical research.

Medical progress is based on research which ultimately must rest in part on experimentation involving human subjects.

In the field of biomedical research a fundamental distinction must be recognized between medical research in which the aim is essentially diagnostic or therapeutic for a patient, and medical research, the essential object of which is purely scientific and without implying direct diagnostic or therapeutic value to the person subjected to the research.

Special caution must be exercised in the conduct of research which may affect the environment, and the welfare of animals used for research must be respected.

Because it is essential that the results of laboratory experiments be applied to human beings to further scientific knowledge and to help suffering humanity, the World Medical Association has prepared the following recommendations as a guide to every physician in biomedical research involving human subjects. They should be kept under review in the future. It must be stressed that the standards as drafted are only a guide to physicians all over the world. Physicians are not relieved from criminal, civil and ethical responsibilities under the laws of their own countries.

**I. BASIC PRINCIPLES**

1. Biomedical research involving human subjects must conform to generally accepted scientific principles and should be based on adequately performed laboratory and animal experimentation and on a thorough knowledge of the scientific literature.

2. The design and performance of each experimental procedure involving human subjects should be clearly formulated in an experimental protocol which should be transmitted for consideration, comment and guidance to a specially appointed committee independent of the investigator and the sponsor provided that this independent committee is in conformity with the laws and regulations of the country in which the research experiment is performed.

3. Biomedical research involving human subjects should be conducted only by scientifically qualified persons and under the supervision of a clinically competent medical person. The responsibility for the human subject must always rest with a medically qualified person and never rest on the subject of the research, even though the subject has given his or her consent.

4. 4. Biomedical research involving human subjects cannot legitimately be carried out unless the importance of the objective is in proportion to the inherent risk to the subject.

5. Every biomedical research project involving human subjects should be preceded by careful assessment of predictable risks in comparison with foreseeable benefits to the subject or to others. Concern for the interests of the subject must always prevail over the interests of science and society.

6. The right of the research subject to safeguard his or her integrity must always be respected. Every precaution should be taken to respect the privacy of the subject and to minimize the impact of the study on the subject's physical and mental integrity and on the personality of the subject.

7. Physicians should abstain from engaging in research projects involving human subjects unless they are satisfied that the hazards involved are believed to be predictable. Physicians should cease any investigation if the hazards are found to outweigh the potential benefits.

8. In publication of the results of his or her research, the physician is obliged to preserve the accuracy of the results. Reports of experimentation not in accordance with the principles laid down in this Declaration should not be accepted for publication.

9. In any research on human beings, each potential subject must be adequately informed of the aims, methods, anticipated benefits and potential hazards of the study and the discomfort it may entail. He or she should be informed that he or she is at liberty to abstain from participation in the study and that he or she is free to withdraw his or her consent to participation at any time. The physician should then obtain the subject's freely-given informed consent, preferably in writing.

10. When obtaining informed consent for the research project the physician should be particularly cautious if the subject is in a dependent relationship to him or her or may consent under duress. In that case the informed consent should be obtained by a physician who is not engaged in the investigation and who is completely independent of this official relationship.

11. In case of legal incompetence, informed consent should be obtained from the legal guardian in accordance with national legislation. Where physical or mental incapacity makes it impossible to obtain informed consent, or when the subject is a minor, permission from the responsible relative replaces that of the subject in accordance with national legislation. Whenever the minor

child is in fact able to give consent, the minor's consent must be obtained in addition to the consent of the minor's legal guardian.

12. The research protocol should always contain a statement of the ethical considerations involved and should indicate that the principles enunciated in the present Declaration are complied with.

**II. MEDICAL RESEARCH COMBINED WITH PROFESSIONAL CARE**

**(Clinical Research)**

1. In the treatment of the sick person, the physician must be free to use a new diagnostic and therapeutic measure, if in his or her judgement it offers hope of saving life, re-establishing health or alleviating suffering.

2. The potential benefits, hazards and discomfort of a new method should be weighed against the advantages of the best current diagnostic and therapeutic methods.

3. In any medical study, every patient - including those of a control group, if any - should be assured of the best proven diagnostic and therapeutic method. This does not exclude the use of inert placebo in studies where no proven diagnostic or therapeutic method exists.

4. The refusal of the patient to participate in a study must never interfere with the physician-patient relationship.

5. If the physician considers it essential not to obtain informed consent, the specific reasons for this proposal should be stated in the experimental protocol for transmission to the independent committee (I, 2).

6. The physician can combine medical research with professional care, the objective being the acquisition of new medical knowledge, only to the extent that medical research is justified by its potential diagnostic or therapeutic value for the patient.

**III. NON-THERAPEUTIC BIOMEDICAL RESEARCH INVOLVING HUMAN SUBJECTS (Non-Clinical Biomedical Research)**

1. In the purely scientific application of medical research carried out on a human being, it is the duty of the physician to remain the protector of the life and health of that person on whom biomedical research is being carried out.

2. The subject should be volunteers - either healthy persons or patients for whom the experimental design is not related to the patient's illness.

3. The investigator or the investigating team should discontinue the research if in his/her or their judgement it may, if continued, be harmful to the individual.

4. In research on man, the interest of science and society should never take precedence over considerations related to the wellbeing of the subject.

**Supplementary appendix 2: Additional methods**

*Trial secondary outcomes*

Secondary outcome measures of safety and tolerability included: Treatment compliance as determined by patient withdrawal and the frequency of severe (S)AE and AEs; changes in QoL questionnaires; liver fibrosis as determined by Enhanced Liver Fibrosis (ELF; iQUR Ltd, Royal Free Hospital, UK) (1) and Fibroscan; liver function as determined by aspartate aminotransferase (AST), alanine aminotransferase (ALT), alkaline phosphatase (ALP), gamma glutamyl transferase (GGT), bilirubin, albumin, and international normalized ratio (INR) levels (all measured by the Clinical Laboratory Services, Queen Elizabeth Hospital, Birmingham, UK), and composite risk scores (Mayo PSC Risk score and Model for End Stage Liver Disease (MELD)); disease progression as determined by LiverMultiscan liver inflammation and fibrosis (LIF) scores (2); and, disease activity as assessed by measuring changes in sVAP-1/SSAO expression.

*Serum processing for measurement of circulating timolumab, sVAP-1, circulating markers of fibrosis, and amine oxidase activity*

Venous blood was collected into Vacutainer SST tubes (BD). Tubes were inverted gently five times and left to clot for 30-60 minutes at room temperature before centrifuging at 1,300 x *g* for 10 minutes in a swing-bucket rotor (or 15 minutes in a fixed-angle rotor) at room temperature. 1 mL aliquots of serum were stored in screw-top cryovials at -80$^{o}$C.

*Measurement circulating markers of fibrosis*

Formation of collagens (Pro-C3, P4NP7S, Pro-C5), degradation of interstitial matrix (C3M, C4M2, C5M), and more exploratory markers of fibrosis (COL18N, NL-NE, and ELM7) were analysed by Nordic Bioscience (Denmark) using validated competitive ELISAs. Levels of each marker were measured on days one (prior to first infusion), 50 (treatment mid-point), 99 (treatment endpoint) and 120 (follow-up). Some samples had values below the limit of detection for proC5 (41.6 ng/mL), C5M (2.0 ng/mL), COL18N (4.8 ng/mL), NL-NE (3.2 ng/mL), ELM7 (0.90 ng/mL). The concentration for these was set to zero. A total of 14 patients were included in this analysis.

*Serum amine oxidase activity measurement*

Serum amine oxidase activity was measured using an Amplex UltraRed-based assay to detect the evolution of hydrogen peroxide. Each well of a black-walled 96-well plate (ThermoFisher, UK) contained 200 μM Amplex Ultrared (ThermoFisher, UK), 1 U/mL horseradish peroxidase (Merck, UK), 1 mM VAP-1 substrate benzylamine (Merck) and diluted serum sample (mixed 1:1 with Dulbecco’s phosphate buffered saline, ThermoFisher, UK) in the absence or presence of the amine oxidase inhibitor LJP1586 (1 μM, a gift from S.Jalkanen, University of Turku, Finland). A calibration curve with known concentrations of hydrogen peroxide (Merck) was prepared in parallel, in addition to a positive control (containing 50 ng recombinant VAP-1 (BioLegend, UK)) or off-the-clot human serum (TCS)). Amplex UltraRed fluorescence (excitation: 530 nm, emission 590 nm, with slit widths 25 nm and 35 nm respectively, gain of 40) was then monitored using a Synergy HT (Biotek) plate reader over 1 hour at 37$^{o}$C with readings taken every 5 minutes. Steady-state enzyme activity was calculated by subtracting the background rate acquired in the presence of LJP1586 and converted

to moles of hydrogen peroxide evolved by comparison to the calibration curve. Serum from 13 patients were measured for each visit, and subsequently aggregated into pre-treatment (visits 1-3: screening visits 1 and 2, pre-first infusion visit 3 (day 1)), treatment (infusion visits 4-9: days 8, 22, 36, 50, 64, and 78) and post-treatment (follow-up visits 10-11: day 99 and 120) groups.

*Determination of circulating immune cell populations by flow cytometry*

Venous blood was collected into 10 mL Vacutainer EDTA tubes (BD) and inverted gently 8 times prior to processing or shipping. Samples were processed within four hours of receipt or stored at 4$^{o}$C and processed as soon as possible and within 24 hours. Samples were collected at each visit for 10 patients. Peripheral blood mononuclear cells (PBMCs) were isolated over Lymphoprep (Stemcell Technologies, UK) at 700 x *g* for 20 minutes. PBMCs were washed with saline (ThermoFisher, UK) and cell pellets resuspended at a concentration of 10x10$^{6}$ cells/mL in CryoStor CS5 freezing media (Merck, UK). Cells were transferred to a Biocision CoolCell (VWR Int.) before being transferred to -80$^{o}$C until required.

Vials of PBMCs were removed from frozen storage and washed once with PBMC media (RPMI-1640 (Merck, UK) containing 10% v/v foetal bovine serum (Merck, UK) and 200 IU/mL interleukin (IL)-2 (Miltenyi Biotec, UK)). Cells were pelleted by centrifugation at 800 x *g* for 5 minutes and rested overnight in an incubator at 37$^{o}$C, 5% $CO_2$ in PBMC media. Cells were then resuspended in saline containing a viability dye (LIVE/DEAD fixable yellow dead cell stain kit, ThermoFisher) before being split into separate tubes for further labelling with the following flow cytometry antibodies: Panel 1 (T-cells) – CD127-FITC (ThermoFisher), CD8-PECF594 (BD Bioscience), CD4-PerCPCy5.5 (ThermoFisher), CD25-BV421 (BD Bioscience), CD3-APC-

eFluor780 (BD Bioscience); Panel 2 (delta 2 TCR) – Vδ2 TCR-FITC (BD Bioscience), CD45-PE (ThermoFisher), CD3-APC-eFluor780 (BD Bioscience); Panel 3 (NK, NKT, Th17, B cells) – CD56-PE (ThermoFisher), CD4-PerCPCy5.5 (ThermoFisher), CD161-PE-Cy7 (ThermoFisher), CD19-APC (ThermoFisher), CD3-APC-eFluor780 (BD Bioscience); Panel 4 (Monocytes) – CD66b-FITC (BD Bioscience), CD3/CD56/CD19-PE (ThermoFisher), CD16-PECF594 (BD Bioscience), HLA-DR-eFluor450 (ThermoFisher), CD14-APC-eFluor780 (ThermoFisher). Samples were then fixed with 1% w/v formaldehyde solution (BDBioscience), then analysed using a CyAn ADP flow cytometer (Beckman Coulter) and FlowJo software (v10.0.8). Isotype controls were used to select cell populations and fluorescence-minus-one controls (FMO) were used to define populations such as CD161 and CD16 (see gating strategy, Figure S1A). Initial populations were gated by forward and side scatter (FS and SS respectively), singlets were selected by FS/pulse width and live cells as VioGreen- population (Live/Dead). Gating strategies for CD3+CD4+, CD3+CD8+ T-cells and Treg (CD3+CD4+CD127+) (Fig S1A); CD3+CD45+delta2 TCR+ cells (Fig S1B); identification of B cells (CD3-CD19+), NK cells (CD3-CD56+), NKT cells (CD3+CD56+), and Th17 cells (CD3+CD4+CD161+, fluorescence-minus-one (FMO) control also shown for CD161 antibody) (Fig S1C); and for monocyte populations, initially gated as CD3-CD19-Cd56-CD66b-HLADR+ and further differentiated as classical (CD14+CD16-), intermediate (CD14+CD16+) and non-classical (CD14-CD16+) populations (FMO for CD16 shown to illustrate specificity of staining) (Fig S1D) are shown. Data are presented as percentage total viable cell population averaged across three timepoints; pre-treatment (visits 1-3: screening visits 1 and 2, pre-first infusion visit 3 (day 1)), treatment (infusion visits 4-9: days 8, 22, 36, 50, 64, and 78) and post-treatment (follow-up visits 10-11: day 99 and 120) groups.

*Additional statistical analysis methods*

Phase I: Dose Confirmation

In the event that the dose limiting toxicity (DLT) rate was acceptable, but the pharmacokinetic (PK) data did not meet the success criteria, the trial allowed for a 3+3 design, using escalating doses of timolumab (BTT1023) to 12 mg/kg and possibly 16 mg/kg. After a cohort of three patients, if no DLTs at day 99 were observed, the dose would be expanded by three patients. If the DLT rate remained acceptable but the PK levels still did not meet the success criteria, the dose would be escalated. If at the highest dose the PK data suggested insufficient levels of timolumab, then the trial would be stopped.

Alternatively, if the PK values were found to be too high (such as resulting in trough levels consistently exceeding 100 µg/mL), then there was potential to de-escalate the dose in agreement with the Data Monitoring Committee and after regulatory approval. The trial was to be stopped at any stage where patient safety was compromised.

Phase II: Simon's Two-Stage Minimax Design

The two-stage design incorporated an interim analysis of the accumulating data after 18 patients had received the trial treatment at the confirmed dose. At this point the design required that at least 3/18 patients to have had a successful response to allow the trial to continue. If the stage 1 criterion was not met, and less than 3/18 evaluable patients had a successful response, the trial was to stop early. However, if the criterion was met, then further patients could be recruited until 37 evaluable patients were recruited. At the final assessment, the treatment would be concluded to be acceptably active if at least 9/37 patients had a successful response.

Statistical analyses for the dose-confirmatory phase of the trial were conducted on patients who completed the full seven infusions as scheduled in the trial protocol. Statistical analyses for the dose-expansion, phase II portion of the trial were conducted on a modified intention-to-treat basis whereby only patients who had received at least one infusion at the confirmed dose of timolumab were be analysed. A sensitivity analysis was also performed using the per-protocol population i.e., those patients who completed the full seven infusions as scheduled in the trial protocol.

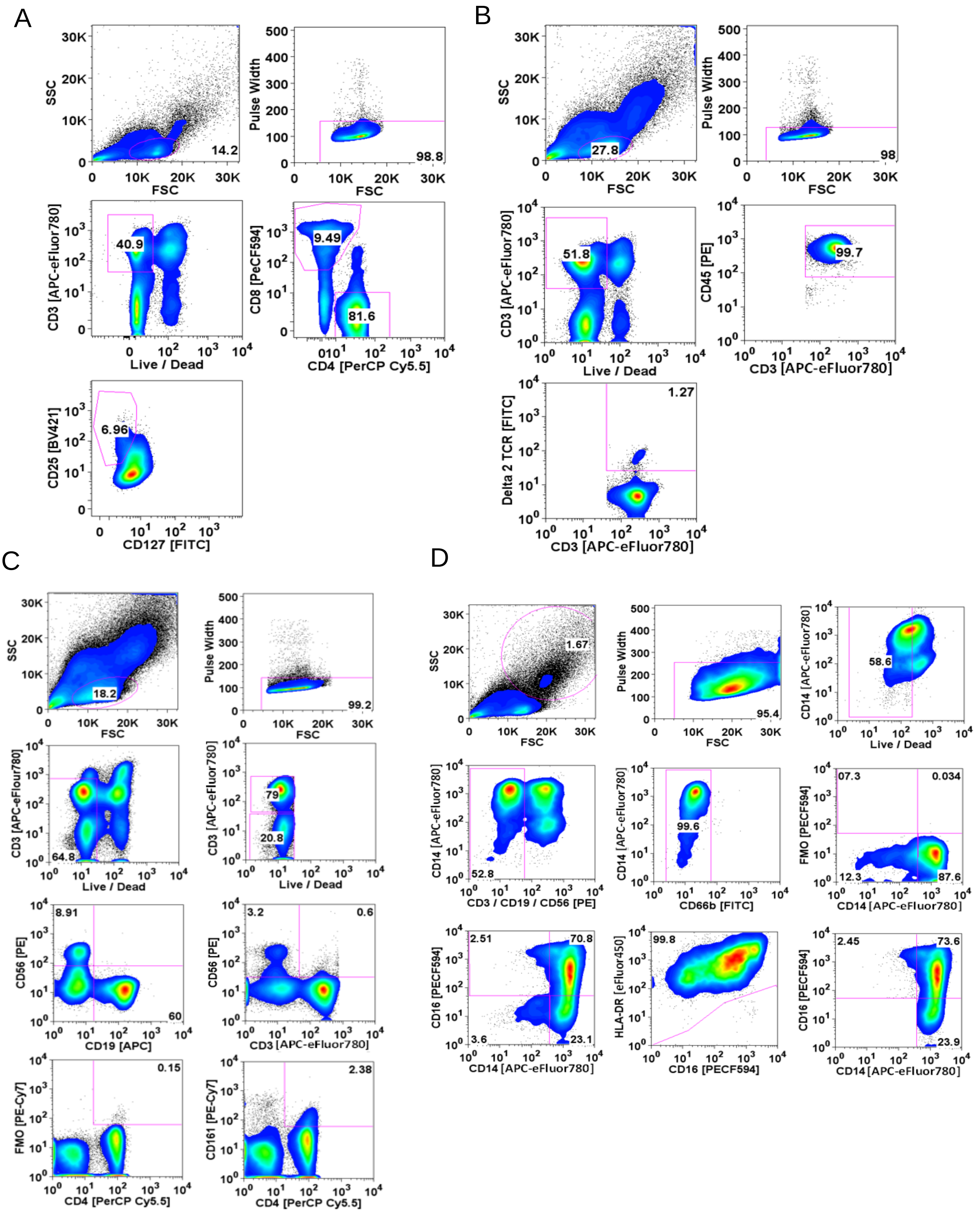


*Fig. S2: Flow cytometry gating strategy*

Gating strategies for determination of circulating immune cell populations by flow cytometry are shown. See text for details.

*References*

1. Vali Y, Lee J, Boursier J, Spijker R, Löffler J, Verheij J, et al. Enhanced liver fibrosis test for the non-invasive diagnosis of fibrosis in patients with NAFLD: A systematic review and meta-analysis. Journal of Hepatology. 2020;73(2):252-62.

2. Pavlides M, Banerjee R, Sellwood J, Kelly CJ, Robson MD, Booth JC, et al. Multiparametric magnetic resonance imaging predicts clinical outcomes in patients with chronic liver disease. J Hepatol. 2016;64(2):308-15.

**Supplementary appendix 3: BUTEO trial statistical analysis plan**

# BUTEO

## Statistical Analysis Plan

*A single arm, two-stage, multi-centre, phase II clinical trial investigating the safety and activity of the use of BTT1023, a human monoclonal antibody targeting vascular adhesion protein (VAP-1), in the treatment of patients with primary sclerosing cholangitis (PSC)*

Version: 2.0 27th January, 2020

**Sponsor:** University of Birmingham **Number:** RG_13-0271.2.4

**EudraCT number:** 2014-002393-37

**Cancer research UK Clinical Trials Unit Reference:** HE2022

**Author(s):**

**Name:** Victoria Homer **Signature:**

**Trial role:** Trial Biostatistician

**Date:**

**Reviewed and approved by:**

**Name:** Daniel Slade **Signature:**

**Trial role:** Senior Biostatistician

**Date:**

**Name:** Prof Philip Newsome **Signature:**

**Trial role:** Chief Investigator

**Date:**

Cancer Research UK Clinical Trials Unit
@CRCTU

## Key personnel involved in the Statistical Analysis Plan:

| Name | Trial role |
|---|---|
| Dr Gideon Hirschfield | Chief Investigator |
| Professor David Adams | Co-Investigator |
| Richard Fox | Senior Biostatistician (former) |
| Amanda Kirkham | Trial Biostatistician (former) |
| Anna Rowe | Senior Trial Coordinator |
| Jess Douglas | Trial Coordinator |
| Victoria Homer | Trial Biostatistician |
| Daniel Slade | Senior Biostatistician |

## Document Control Sheet

| Statistical Analysis Plan version: | Reason for update: |
|---|---|
| Version 0.1 12-08-2016 | Initial draft |
| Version 0.2 21-10-2016 | Initial report with amendments |
| Version 0.3 14-11-2016 | Initial report with further amendments |
| Version 0.4 21-12-2016 | Re-structured and amended |
| Version 0.5 23-02-2017 | Amendments |
| Version 1.0 27th April 2017 | Sign off |
| Version 1.1 9th May 2017 | Amendment to baseline definition |
| Version 1.2 12th April 2018 | Section 8.1.2: Addition of PK data to be analysed and further exploratory analyses maybe performed;<br>Section 8.7: Inclusion of exploratory subgroup analyses maybe performed. |
| Version 1.3 24th January 2020 | Section 8.1.1 and 8.1.2: Alterations to definitions of baseline periods for calculations of (absolute) differences and percentage changes<br>Section 8.1.2: Addition of sVAP-1/SSAO biomarker<br>Section 8.1.3: Addition of exploratory biomarker analyses |
| | |

## CONTENTS

| | Page |
|---|---|
| **1. Introduction** | **4** |
| 1.1. Purpose of the Statistical Analysis Plan | 4 |
| 1.2. Summary of the Trial | 4 |
| 1.2.1. Trial Design | 4 |
| 1.2.2. Objectives | 4 |
| 1.2.2.1. Dose Confirmatory Phase | 4 |
| 1.2.2.2. Simon's Two-Stage Phase II | 4 |
| 1.2.3. Outcome Measures | 4 |
| 1.2.3.1. Dose Confirmatory Phase | 4 |
| 1.2.3.2. Simon's Two-Stage Phase II | 5 |
| 1.2.4. Patient Population | 5 |
| 1.2.5. Trial Duration | 5 |
| 1.2.6. Trial Treatment Schema | 6 |
| 1.2.7. Schedule of Events | 7 |
| 1.2.8. Trial Design Flow Diagram | 10 |
| **2. Timing and Reporting of Interim and Final Analyses** | **11** |
| 2.1. Dose Confirmatory Phase | 11 |
| 2.2.1. Phase II – Interim Analysis (stage 1) | 11 |
| 2.2.2. Phase II – Final Analysis (stage 2) | 11 |
| **3. Recruitment and Randomisation** | **11** |
| 3.1. Recruitment | 11 |
| 3.2. Randomisation | 12 |
| 3.3. Ineligible Patients | 12 |
| **4. Data Quality** | **12** |
| **5. Trial Population** | **12** |
| 5.1. Safety Population | 13 |
| 5.2. Evaluable Patients | 13 |
| **6. Treatment Received Reporting** | **13** |
| **7. Toxicity and Safety Reporting** | **13** |
| **8. Analyses** | **14** |
| 8.1. Definition and Calculation of Outcome Measures | 14 |
| 8.1.1. Primary Outcome Measure | 14 |
| 8.1.2. Secondary Outcome Measures | 14 |
| 8.2. Descriptive Analyses | 15 |
| 8.3. Hypothesis Testing for the Primary Outcome | 15 |
| 8.3.1. Null Hypothesis | 15 |
| 8.3.2. Methods | 16 |
| 8.3.3. Sample Size Calculations | 16 |
| 8.3.3.1. Dose Confirmatory Stage | 16 |
| 8.3.3.2. Simon's Two-Stage Design | 16 |
| 8.4. Hypothesis Testing for Secondary Outcome Measures | 17 |
| 8.5. Additional Analyses | 17 |
| 8.6. Decision Criteria | 17 |
| 8.6.1. Dose Confirmatory Stage | 17 |
| 8.6.2. Simon's Two-Stage Phase II | 17 |
| 8.7. Subgroup Analyses | 17 |
| **9. Health Economics** | **17** |
| **10. Statistical Software** | **17** |
| **11. Storage and Archiving** | **18** |
| **12. References** | **18** |

# INTRODUCTION

## 1.1 Purpose of the Statistical Analysis Plan

This Statistical Analysis Plan (SAP) provides guidelines for the analysis and presentation of results for the Buteo trial. This plan, along with all other documents relating to the analysis of this trial, will be stored in the 'Statistical Documentation' section of the Trial Master File. The statistical analysis will be carried out by the Trial Statistician.

## 1.2 Summary of the Trial

### 1.2.1 Trial Design

Begins with an initial dose confirmatory stage continuing on into a Simon's two-stage, single-arm, open-label, multi-centre phase II trial.

### 1.2.2 Objectives

#### 1.2.2.1 Dose Confirmatory Phase

- To determine a dose of BTT1023 that provides an acceptable level of pharmacokinetic (PK) activity and is deemed to be safe, meeting the acceptable Dose Limiting Toxicity (DLT) level.

  This will be done by evaluating:
  - The number of DLTs per dose level of BTT1023
  - The PK data of BTT1023

#### 1.2.2.2 Simon's Two-Stage Phase II

***Primary objectives:***

- To evaluate the safety, effective dose, and tolerability of BTT1023 in patients with PSC
- To determine the activity of the anti-VAP-1 antibody BTT1023 in patients with PSC as measured by a decrease in alkaline phosphatase levels (ALP) (primary endpoint) with secondary endpoints to include various measures of liver injury and fibrosis.

***Secondary objectives:***

- To determine the mechanisms of action of BTT1023 through in vivo assessment of VAP-1/semicarbazide-sensitive amine oxidase (SSAO) enzyme activity and immune cell function.
- To evaluate the potential of a novel Magnetic Resonance Imaging (MRI) based assessment of liver fibrosis and biliary strictures for assessing therapeutic response in PSC.
- Assess the use of sVAP-1/SSAO as a biomarker to monitor disease progression in PSC.

### 1.2.3 Outcome Measures

#### 1.2.3.1 Dose Confirmatory Phase

- ***Dose Limiting Toxicity (DLT) Definition***
  - Any grade 4 or 5 adverse event (AE) as defined by CTCAE v4.0 (Protocol Appendix 2) and considered to be at least possibly related to BTT1023 treatment
  - Grade 3 Cytokine Release Syndrome considered to be at least possibly related to BTT1023 treatment
  - The confirmatory stage DLT reporting period is from treatment visit 3 up to visit 10 (day 99 follow-up visit)
  - The acceptable level of DLTs per dose is 1 in 6 patients (~17%)
- ***Pharmacokinetic (PK) Data:*** *t*o confirm that the BTT1023 dose is at the required activity level, the trough levels of BTT1023 must meet the stipulated success criteria of 3μg/ml free circulating antibody as measured during the dose confirmatory stage; if PK results show the levels are too low then the dose will be escalated; and if the PK results show the levels are too high then the dose can be reduced. Data will be produced and analysed by Biotie.

#### 1.2.3.2 Simon's Two-Stage Phase II

***Primary outcome measures:***

- **Response at day 99:** a reduction in serum alkaline phosphatase (ALP) by 25% or more from baseline to day 99.

***Secondary outcome measures:***

- **Safety and tolerability:** Treatment compliance (including patient withdrawal), Serious Adverse Event (SAE) and Adverse Event (AE) frequency

- **Response at day 99 in:**
  - **Quality of life questionnaires:** EQ-5D, Fatigue Severity Scale, Pruritus Visual Analogue Score (VAS), inflammatory bowel disease (IBD) Diaries (if applicable).
  - **Tests of liver fibrosis:** enhanced liver fibrosis (ELF) and Fibroscan.
  - **Individual markers of liver biochemistry and function:** aspartate transaminase [AST], alanine transaminase [ALT], ALP, gamma glutamyl transferase [GGT], bilirubin, Albumin, International Normalised Ratio [INR]) and composite risk scores (Mayo PSC risk score and model for end-stage liver disease [MELD] score
  - **LiverMultiscan® MRI imaging:** Liver MRI is an emerging method for monitoring liver disease and its treatment
  - **sVAP-1/SSAO**: as a biomarker of liver disease activity across the study period

### 1.2.4 Patient Population

Patients who have been clinically diagnosed with PSC, as confirmed in one of the trial centres, and who also meet the inclusion and exclusion criteria.

### 1.2.5 Trial Duration

It is anticipated that recruitment will take 2 years. The treatment period is 78 days and all patients will be followed up until day 120, i.e. 42 days after the last administration of study treatment.

### 1.2.6 Trial Treatment Schema

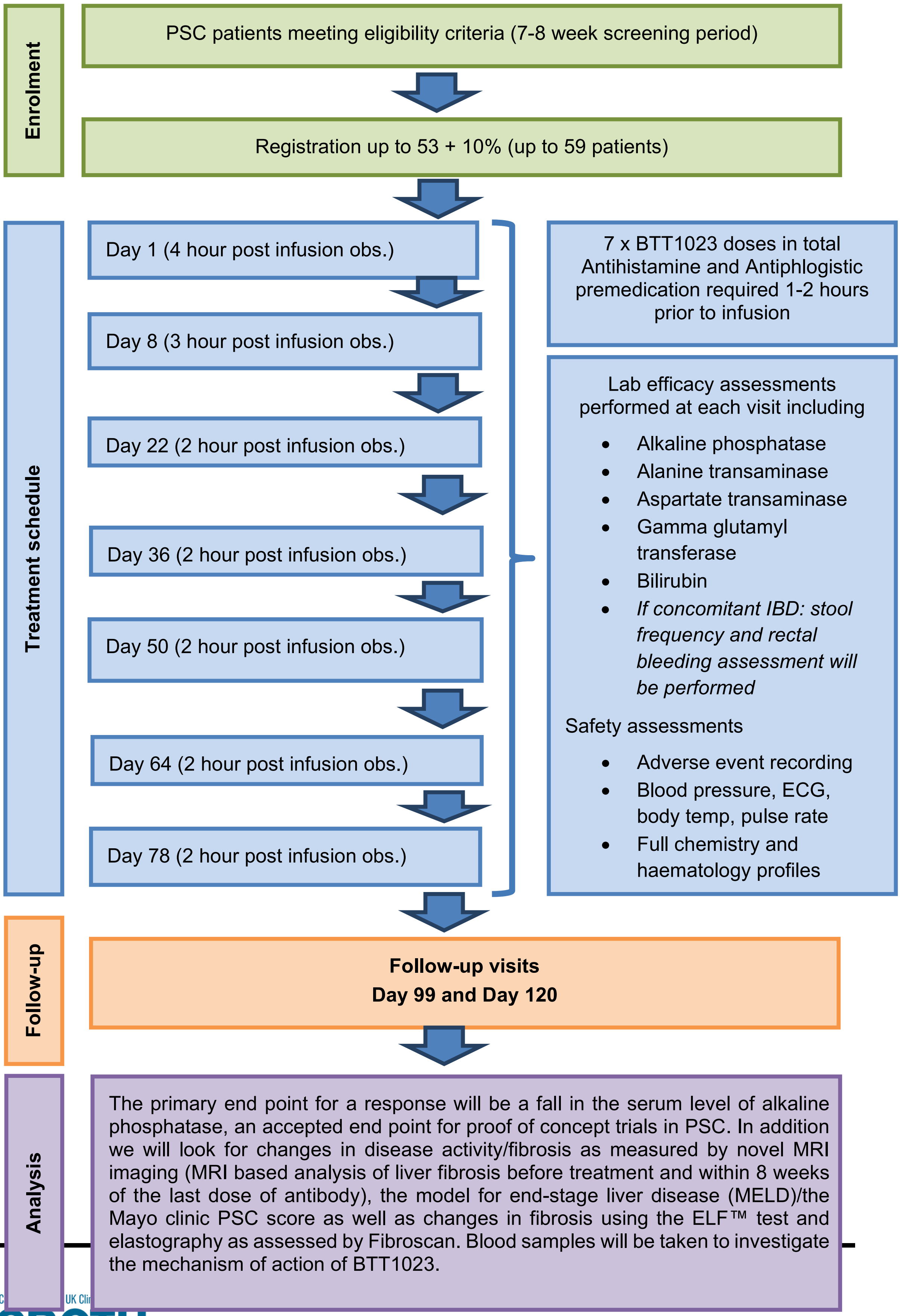


CRCTU-STA-QCD-002, version 1.0

### 1.2.7 Schedule of Events

*Table 1: Schedule of events for the duration of the Buteo trial*

| | Screening | | Treatment | | | | | | | Follow-up | |
|---|---|---|---|---|---|---|---|---|---|---|---|
| | Screening Visit 1 (-8 weeks) | Screening Visit 2 (-1 week) | Visit 3 (Infusion visit Day 1) | Visit 4# (Infusion visit Day 8) | Visit 5# (Infusion visit Day 22) | Visit 6# (Infusion visit Day 36) | Visit 7# (Infusion visit Day 50) | Visit 8# (Infusion visit Day 64) | Visit 9# (Infusion visit Day 78) | Visit 10# (Day 99) | Visit 11# (Day 120) |
| Informed consent | X | | | | | | | | | | |
| Eligibility assessment | X | X | | | | | | | | | |
| Patient history | X | X | | | | | | | | | |
| Physical exam | X | | X | | X | | X | | X | X | X |
| Vital signs [1] | X | | X | X | X | X | X | X | X | X | X |
| Vital signs during and post infusion [2] | | | X | X | X | X | X | X | X | | |
| ECG [3] | X | | X | X | X | X | X | X | X | X | |
| BTT1023 infusion | | | X | X | X | X | X | X | X | | |
| Standard blood tests[4] | X | X | X | X | X | X | X | X | X | X | X |
| Standard blood tests 2hrs post infusion [5] | | | X | X | X | X | X | X | X | | |
| Blood + serum sample for biomarker and research blood analysis | X | X | X | X | X | X | X | X | X | X | X |

CRCTU-STA-QCD-002, version 1.0

| | Screening | | Treatment | | | | | | | Follow-up | |
|---|---|---|---|---|---|---|---|---|---|---|---|
| | Screening Visit 1 (-8 weeks) | Screening Visit 2 (-1 week) | Visit 3 (Infusion visit Day 1) | Visit 4# (Infusion visit Day 8) | Visit 5# (Infusion visit Day 22) | Visit 6# (Infusion visit Day 36) | Visit 7# (Infusion visit Day 50) | Visit 8# (Infusion visit Day 64) | Visit 9# (Infusion visit Day 78) | Visit 10# (Day 99) | Visit 11 # (Day 120) |
| Blood serum for PK[6] | | | X | X | X | X | X | X | X | X | X |
| Blood serum for Anti-Drug Antibodies (ADA) | X | | X[8] | | | | X[8] | | X[8] | X | X |
| Blood serum for PD (sVAP-1) | X | | X[9] | X[8] | X[8] | X[8] | X[8] | X[8] | X[10] | X | X |
| Pregnancy test (female patients only) | X | X | | | X | | X | | X | | X |
| MRI scan [7] | X | | | | | | | | | | X |
| Mayo clinic PSC score | | X | | | | | | | | X | |
| MELD score | | X | | | | | | | | X | |
| Serum ELF | | X | | | | | | | | X | |
| Fibroscan® | | X | | | | | | | | X | |
| T-SPOT.TB test | X | | | | | | | | | | |
| Quality of life questionnaires | | X | | | | X | | | | X | |
| Adverse/ Clinical events | X | | X | X | X | X | X | X | X | X | X |
| Concomitant medications | X | X | X | X | X | X | X | X | X | X | X |

CRCTU-STA-QCD-002, version 1.0

1 Vital signs: heart rate, blood pressure, temperature and respiratory rate to be performed pre-infusion Visits 3-11

2 Vital signs: heart rate, blood pressure, temperature and respiratory rate to be performed:

- **Day 1:** 30 and 60 minutes after the start of the infusion, 30, 60, 90, 120, 180 and 240 minutes post end of drug infusion.
- **Day 8:** 30 and 60 minutes after the start of the infusion, 30, 60, 90, 120 and 180 minutes post end of drug infusion.
- **Day 22, 36, 50, 64 and 78:** 30 and 60 minutes after the start of the infusion, 30, 90, and 120 minutes post end of drug infusion.

3 12 lead ECG to be performed pre-infusion and 60 minutes after the start of infusion.

4 Full blood count (FBC), urea, electrolytes, liver function tests (LFTs), international normalised ratio (INR) to be performed pre-infusion **Day 1, 8, 22, 36, 50, 64, 78, 99 and 120** *(see visit schedule for +/- days applicable to each visit).*

5 FBC, urea, electrolytes, LFTs, INR to be performed 120 minutes after the start of infusion.

6 Blood serum for pharmacokinetics. See Tables 6-8 for timings.

7 MRI within 8 weeks of start of treatment and 2 weeks of end of treatment (last dose of antibody).

8 Blood serum samples: Pre-infusion.

9 Blood serum samples: Pre-infusion, 180 and 360 minutes and 24 hours post end of drug infusion.

10 Blood serum samples: Pre-infusion, 120 minutes and 24 hours post end of drug infusion.

# see Section 7 for +/- days allowed at each visit

## 1.2.8 Trial Design Flow Diagram

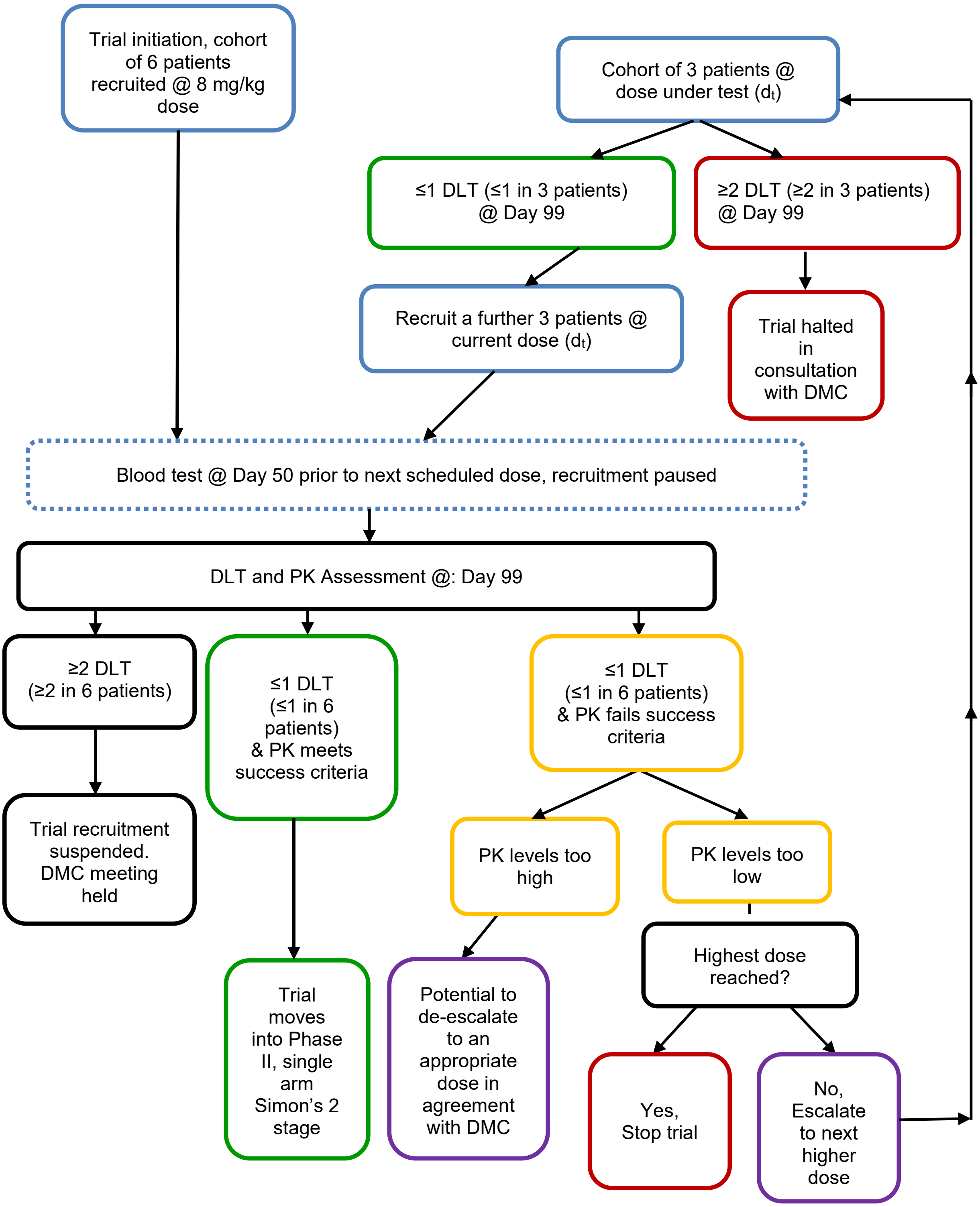


Flow diagram showing the trial decision guidelines for the run-in period of the trial design incorporating a conventional 3+3 cohort design if dose escalation is required.

# 2. TIMING AND REPORTING OF INTERIM AND FINAL ANALYSES

## 2.1 Dose Confirmatory Phase

- Initial dose confirmatory phase analyses comprise of safety, tolerability and minimum effective dose (MED) estimation
- Safety and tolerability data will be collated and analysed after day 99 for each recruited patient and be carried out by CRCTU
- The MED estimation will be obtained from pharmacokinetic (PK) analysis of blood samples taken at the day 50 treatment visit – this work will be carried out by Biotie and results supplied to CRCTU
- This analysis and reporting schedule will be performed for each subsequent dose level (12, 16 mg/kg), should dose escalation be necessary
- All data available will be reviewed by the DMC after each cohort of six patients during the dose confirmatory stage

## 2.2 Phase II – Interim Analysis (Stage 1)

- Only one interim analysis is planned and will take place once 18 patients have been evaluated for the primary outcome (response in ALP level, measured from baseline to day 99)
- The interim report will be prepared and supplied to the DMC when the study has recruited and evaluated 18 patients at the chosen MED dose of BTT1023 (including those recruited on the MED dose during the dose confirmatory stage), or annually whichever is earliest
- The DMC report will include (but not limited to): recruitment, CRF return rates, data quality, protocol deviations, withdrawal rates, patient characteristics, treatment compliance, primary and secondary outcome measures and toxicity/adverse events
- The DMC may consider closing the trial early or request more frequent meetings if the recruitment rate or data quality are unacceptable, if there are safety concerns, or if the interim results indicate lack of treatment efficacy
- An emergency meeting may be convened at any time if a safety issue of concern is identified

## 2.3 Phase II – Final Analysis (Stage 2)

- The final analysis report containing all available safety data, together with primary and secondary outcomes measures analyses will be prepared once 37 evaluable patients have reached the primary endpoint
- The final analysis will be reported within one year of the date of Last Patient Last Visit (LPLV) – defining the end of trial

# 3. RECRUITMENT AND RANDOMISATION

## 3.1 Recruitment

The analyses will include:

- Date the snapshot was taken
- Dates when the trial opened and closed for recruitment
- Recruitment over time (monthly or quarterly) and an average monthly recruitment rate
- Recruitment by site and/or clinician
- Cross-tabulation of recruitment by site / clinician (rows – in order of opening) and time interval (columns) – time when sites not open is shaded out

***Data sources:*** Registration Form

## 3.2 Randomisation

This is a single arm trial so no randomisation is required for this trial design.

## 3.3 Ineligible Patients

Ineligible patients are defined as those registered patients who are subsequently found to not meet the eligibility criteria of the trial. The number (%) of ineligible patients and reasons for their ineligibility will be reported. Protocol deviations relating to treatment will be reported as part of treatment compliance (section 6).

At the second screening visit, if patients are found to have an ALP level outside the ±25% level of the first screening visit, these patients will be ineligible. This analysis will be performed by the screening team, but CRCTU have been requested to check the findings.

Patients will be ineligible for Phase II trial assessment if they have been recruited in to the trial during the dose confirmatory phase and receiving any dose of BTT1023 that is different to the identified accepted dose level.

***Data sources:*** Biochemistry Forms - screening visit 1 and screening visit 2.

# 4. DATA QUALITY

Completion rates for CRF's and outcome variables will be analysed at least annually in accordance with the current version of the statistical data validation plan. Any missing data or forms will be brought to the attention of the trial coordinator for resolution. The number of expected and completed CRFs will be reported.

Timeliness of treatments and tests received will be checked in order to assess whether a patient remains on protocol. A report on follow up rates up to 120 days will be produced.

Statistical data validation is detailed in the data validation plan.

# 5. TRIAL POPULATION

The trial has two populations: one for the dose confirmatory phase I and another for phase II. Some patients will be in both populations, if they are given what is later found to be the confirmed MED dose for phase II testing – these patients will be included in the phase II data analysis. Statistical analyses will be carried out on a modified intention-to-treat (mITT) basis in which only patients who have received at least one infusion at the confirmed dose of BTT1023 will be analysed.

Patients missing Visit 7 (day 50) blood sample for testing BTT1023 PK levels will be replaced on the trial. Data from replaced patients will be used for safety monitoring but will not form part of the data set for the outcomes measures analyses.

Patient who are recruited but fail to receive any BTT1023 doses will be replaced on the trial.

Patients that cannot be evaluated for the primary outcome (i.e. due to withdrawal or lost to follow up) will be treated as non-responders. Patients that have withdrawn from treatment will continue to be followed in the trial. Protocol deviations relating to treatment will be reported as part of treatment compliance (section 6). Extreme values will be queried by the trial management team and if present at the final analysis stage may be excluded as part of a sensitivity analysis.

Demographic data will be given for patients in the study. This will cover age, sex, ethnicity and clinical baseline outcome characteristics. No hypothesis testing will be carried out. Appropriate summary measures such as mean, median, minimum, maximum and interquartile ranges will be presented for continuous variables; and number and percentage for categorical variables.

## 5.1 Safety Population

Only eligible patients who have received treatment with BTT1023 at any dose level will be evaluated for treatment safety. Patients who are recruited but fail to receive any BTT1023 doses will not be assessed for safety.

## 5.2 Evaluable Patients

- **Confirmatory Stage:** All eligible patients that have received treatment with any dose level of BTT1023
- **Phase II Stage:** All eligible patients are required to have started treatment receiving at least one infusion of the confirmed acceptable dose of BTT1023 as identified in the prior confirmatory stage

***Data sources:*** Registration Form, Ethnicity Form, Vital Signs Form, Treatment Discontinuation Form, Withdrawal Form, Death Form, Medical History Form

# 6. TREATMENT RECEIVED REPORTING

The treatment dose received will depend upon whether a patient is recruited during the dose confirmatory stage or the phase II stage of the trial.

The following will be reported:

- The number of patients receiving treatment
- The dose received by each patient
- The number of cycles received and in total
- Number of dose reductions and delays
- Reasons for dose reductions and delays
- Any concomitant medication received during the trial period
- Level of treatment compliance
- The number of patients who did not start treatment, including the reason(s) for not starting treatment

***Data sources:*** Patient Registration Form, Vital Signs –Treatment Visits3-9 Forms, Concomitant Medication Form, SAE Forms, Treatment Discontinuation Form, Withdrawal Form, Death Form

# 7. TOXICITY AND SAFETY REPORTING

For the duration of the trial, toxicity and safety will be monitored. Figures for AE and SAE data (including any expected SARs) will be reported separately although some AEs maybe duplicated if reported in both AE and SAE categories – where this occurs it will be stipulated.

- The dose confirmatory phase will report:
    - The number of DLT's and number of patients experiencing DLTs will be tabulated per dose level
    - The number of patient withdrawals per dose level
    - The total number of patients will be provided for each dose level of the drug given

    (NB. the acceptable rate of DLTs is 1 in 6 patients receiving the same dose (~17%))

- The Simon's 2-Stage phase 2 will report:
    - The toxicities per cycle (visit) will be given as a listing of number of toxicities by CTCAE v4.0 grade (%) and number of patients
    - The number of related events will be tabulated by visit with line listings given of 3, 4, or 5 adverse events deemed at least possibly related to BTT1023
    - Toxicities will be tabulated by CTCAE v4.0 grade and classification (refer to Protocol appendix 2)
    - Duration of adverse events will be summarised,
    - The frequency of ongoing and resolution of adverse events will be tabulated by treatment visit
    - SAEs/SARs will be reported as frequency and number of patients experiencing them, together with outcome (e.g. death, resolved etc.)

***Data sources:*** BTT1023 Dosing Form, AE Forms, SAE Forms, Treatment Discontinuation Form, Withdrawal Form, Death Form, Deviation Form

# 8. ANALYSES

## 8.1 Definition and Calculation of Outcome Measures

### 8.1.1 Primary Outcome Measure

- **A Reduction in serum alkaline phosphatase**

The primary outcome measure is the response at day 99 in serum alkaline phosphatase (ALP), requiring a reduction of 25% or more from baseline ALP level will be measured at pre-infusion on the first treatment visit (overall trial visit 3), and again at follow-up visit 10 day 99. The response will be calculated using the formulae:

$$\%\ Change = \frac{ALP_{Day99} - ALP_{Visit\ 3\ (pre-infusion)}}{ALP_{Visit\ 3\ (pre-infusion)}} \times 100$$

Using the above formulae, a negative value indicates a reduction, whereas a positive value indicates an increase.
The clinically meaningful reduction required corresponds to a value of -25% or less (≤ -25%). The number and proportion of patients with a clinically meaningful reduction will be reported. The proportion of patients with a clinically meaningful reduction will be calculated as

$$\%\ of\ patients\ with\ clinically\ meaningful\ reduction = \frac{Patients\ with\ clinically\ meaningful\ reduction}{All\ patients\ in\ the\ mITT\ population}$$

Where patients have their follow up visit 10 ALP sample missing, they shall be treated as a non-responder and included in the denominator of the above equation. The number of non-responders for the primary outcome will be reported.
***Data sources:*** Biochemistry Forms, use pre-infusion Treatment visit 1 for baseline measurement and visit 10 (day 99 visit) for post-infusion measurement. Centralised ALP sample form at visit 3 (first treatment visit), and visit 10 (first follow-up visit, day 99).

### 8.1.2 Secondary Outcome Measures

- **Tolerability and treatment compliance**

In addition to safety, lack of tolerability will be assessed based on data obtained from the patient withdrawal form: dose received by each patient, phase recruited to, the number of treatments completed, reasons for withdrawal, and length of time on treatment will be described. Any common tendencies will be reported.
***Data sources****:* Treatment Discontinuation Form, Withdrawal Form, BTT1023 Dose From, Registration Form, Ethnicity Form

- **Questionnaire Data:**

The questionnaire data will be used to calculate any change (improvement or worsening) from baseline to day 99 (visit 10). Where questionnaire data was collected at visit 3 (treatment visit 1), this will be taken to be the baseline visit. This is the case for the following questionnaire data: EQ-5D-5L; FSS; and pruritus VAS. Where questionnaire data was not collected at visit 3, as is the case for IBD diaries, the reference baseline visit will be taken to be screening visit 2. The calculation will be reported as the absolute value together with the percentage change to enable direct comparisons for each patient.
In addition, the data will be plotted as repeated measures over time to demonstrate graphically the behaviour over the treatment period, and will also be analysed using an appropriate repeated measures analysis method. Further exploratory analyses may be performed.
Data will be tabulated together with the dose received by the patient and appropriate patient demographics.
Questionnaires to be analysed are:

- **EQ-5D-5L Health Questionnaire**, (*Data sources: EQ-5D-5L total will be supplied, BTT1023 Dosing Form, Registration Form, Ethnicity Form)*
- **Fatigue Severity Scale (FSS)**, (*Data sources: FSS total will be supplied, BTT1023 Dosing Form, Registration Form, Ethnicity Form)*
- **Pruritus Visual Analogue Scale (VAS)**, *(Data sources: Pruritis VAS total will be supplied, BTT1023 Dosing Form, Registration Form, Ethnicity Form)*
- **Inflammatory Bowel Disease Diaries (IBD)**, *(Data sources: Inflammatory Bowel Disease Diaries)*

● **Measurement Data:**

The outcome measure is to calculate any change (improvement or worsening) from baseline to day 99 (visit 10). The baseline measurement will be taken as the first treatment visit (overall trial visit 3) where available. This is the case for the following measurement data items: ELF; LFTs; PK; and sVAP-1/SSAO biomarker data. Where data was not collected at trial visit 3, the baseline will be taken as screening visit 2. This therefore applies for to: Fibroscan; MELD; Mayo PSC risk score. The calculation will be reported as the absolute value together with the percentage change to enable direct comparisons for each patient.
The data will be plotted as repeated measures over time to demonstrate graphically the behaviour over the treatment period, and will also be analysed using an appropriate repeated measures analysis method. Further exploratory analyses may be performed.

Data will be tabulated together with the dose received by the patient and appropriate patient demographics.

Measurements to be analysed are:

- **Enhanced Liver Fibrosis (ELF)***, (Data sources: ELF Test total will be supplied, BTT1023 Dosing Form, Registration Form, Ethnicity Form)*
- **Fibroscan**, *(Data sources: Fibroscan Form, BTT1023 Dosing Form, Registration Form, Ethnicity Form)*
- **Liver Function Tests (LFT)** including AST, ALT, ALP, GGT, Albumin, Bilirubin – direct and indirect and INR, *(Data sources: All Biochemistry Forms, BTT1023 Dosing Form, Registration Form, Ethnicity Form)*
  *NB: pre-infusion Treatment visit 1 gives baseline and Visit 10 gives day 99 measurements*
- **Model for End Stage Liver Disease (MELD)**, *(Data sources: MELD Score Form & score will be supplied, BTT1023 Dosing Form, Registration Form, Ethnicity Form)*
- **Mayo PSC Risk Score**, *(Data sources: Mayo PSC Risk Score Form & score will be supplied, BTT1023 Dosing Form, Registration Form, Ethnicity Form)*
- **PK Data**, *(Data Source: data supplied by external laboratory and uploaded into the database)*
- **sVAP-1/SSAO Biomarker** *(Data Source: data supplied by external laboratory and uploaded into the database)*

● **MRI Data**

The raw MRI data will be sent to Perspectum Diagnostics and they will forward the results to complete the CRF (e.g. cT1 informs about fibrosis and/or inflammation, T2* informs about iron status and fat % informs about fat). Patients will have a scan pre- and post- therapy. The difference in in the two measurements for each patient (screening visit 1 (day 0) and follow-up visit 11 (day 120)) will be calculated and will be reported as the absolute value and percentage change to enable direct comparison.

In addition the data will be plotted as repeated measures over time to demonstrate graphically the behaviour over the treatment period, and will also be analysed using an appropriate repeated measures analysis method. Further exploratory analyses may be performed.

MRI data will be tabulated together with the dose received by the patient and appropriate patient demographics.

*(Data sources: MRI Form, BTT1023 Dosing Form, Registration Form, Ethnicity)*

### 8.1.3 Exploratory Analyses

Exploratory biomarkers of fibrosis are to be sent to Nordic Biosciences. The outcome measure is to calculate any change (improvement or worsening) from first treatment visit (trial visit 3 overall) to day 99 (visit 10).
The data will be plotted as repeated measures over time to demonstrate graphically the behaviour over the treatment period, and will also be analysed using an appropriate repeated measures analysis method.

Data will be tabulated together with the dose received by the patient and appropriate patient demographics.

The following exploratory biomarkers are to be analysed

- PRO-C3
- P4NP7S
- PRO-C5
- C3M
- CM2
- C5M
- COL-18N
- EL-NE
- ELM7

(Data Source: data supplied by external laboratory and uploaded into the database)

## 8.2 Descriptive Analyses

- Response rate for patients treated on the phase II dose will be reported as a percentage, with all patients who have 25% or more decrease in serum ALP (baseline to day 99) in the numerator and all patients who are response evaluable (including early deaths) in the denominator
- For specific outcome measure calculations see section 8.1, as the trial has not been powered for statistical analyses for any secondary outcome measures, these will undergo exploratory analyses and reported descriptively
- Descriptive statistics will present patient demographic information
- Descriptive statistics will present safety data including: DLT, AE and SAE information
- The mean, median, minimum, maximum and interquartile ranges will be reported for continuous variables where applicable
- Number (count), proportion and percentages will be reported for categorical variables
- The number of evaluable patients for each outcome will be reported.

## 8.3 Hypothesis Testing for the Primary Outcome

### 8.3.1 Null Hypothesis

The null hypothesis for phase II is that the response rate for PSC patients receiving BTT1023 is 15%. (The alternative hypothesis is that the response rate for PSC patients receiving BTT1023 is 30%. The response is the number of patients experiencing a reduction in inflammation, indicated using a reduction in serum alkaline phosphatase (ALP) by 25% or more from baseline to Day 99).

### 8.3.2 Methods

**Stage 1 of accrual:** 18 response evaluable patients will be analysed in the first stage. Using response hypotheses above (section 8.3.1), the treatment will be rejected at the end of the first stage of accrual if only 0, 1 or 2 objectives responses are seen. Otherwise an additional 19 patients will be accrued.
**Stage 2 of accrual:** An additional 19 patients will be accrued. The treatment will be accepted as active if 9 or more objective responses are observed out of the total 37 patients accrued.

### 8.3.3 Sample Size Calculations

The total sample size for the entire trial (dose confirmatory stage plus Phase II) may require up to a maximum of 59 patients, after being inflated to account for potential patient drop-out.

#### 8.3.3.1 Dose Confirmatory Stage

The dose confirmatory stage may require a maximum of 18 patients. The sample size has been based on the classical 3+3 design, investigating three fixed dose increments (8, 12 and 16 mg/kg).

#### 8.3.3.2 Simon's Two-Stage Design

The Simon's two-stage design requires a total of 37 evaluable patients receiving the confirmed dose.

This was calculated using the following parameters: alpha = 0.10, beta = 0.2, $P_0$ = 0.15, $P_1$ = 0.30.

[**Note**: the values for $P_0$ (0.15) and $P_1$ (0.30) correspond to the required reduction in patients experiencing raised levels of ALP from 85% to 70%, i.e. 1-0.85=0.15 and 1-0.70=0.30]

The Simon's two-stage minimax design requires 3/18 successes at the interim analysis to continue, and 9/37 successes at the final analysis.

The phase II Simon's two-stage design incorporates an interim analysis of the accumulating data. The interim analysis (stage-1) takes place once 18 patients have been evaluated for the primary outcome – which is based on the response level of ALP. If three or more successful responses (i.e. 25% or more reduction in ALP level) are observed in stage-1 then the trial will continue into stage-2. Recruitment will not be halted while stage-1 is assessed. Further patients will be recruited during stage-2 in order to obtain the necessary sample size of 37 patients; allowing for 10% patient drop out during trial duration, this number could reach a total of 41 patients being required.
If overall there are nine or more successful responses from 37 evaluable patients then we conclude that the treatment warrants further investigation. If the prescribed patient number is not met then the appropriate decision criterion, corresponding to the total number of evaluable patients, will be selected from table 2. Patients treated at the confirmed dose during the dose confirmatory stage will contribute to the total evaluable patient requirement.

Method for calculation used "Sample Size Tables for Clinical Studies Software", Sze-Huey Tan (2008).

*Table 2: Possible stop/go guidelines and associated error rates for the Simon's Two-Stage design utilised for the Phase II trial component (bold indicates design parameters chosen)*

| Patients (stage 1 interim) | Responses (stage 1 interim) | Patients (total) | Responses (total) | Type-1 Error (α) | Power (1-β) |
|---|---|---|---|---|---|
| 18 | 3 | 34 | 8 | 0.120 | 0.826 |
| 18 | 3 | 35 | 8 | 0.134 | 0.846 |
| 18 | 3 | 36 | 8 | 0.149 | 0.863 |
| **18** | **3** | **37** | **9** | **0.087** | **0.806** |
| 18 | 3 | 38 | 9 | 0.099 | 0.827 |
| 18 | 3 | 39 | 9 | 0.111 | 0.846 |
| 18 | 3 | 40 | 9 | 0.124 | 0.861 |
| 18 | 3 | 41 | 10 | 0.073 | 0.809 |

## 8.4 Hypothesis Testing for Secondary Outcome Measures

No hypothesis testing will be done for the secondary outcome measures.

## 8.5 Additional Analyses

Experimental variables derived from laboratory analyses of peripheral blood will be presented qualitatively and quantitatively, and related to treatment time-points, and clinical and drug variables.

## 8.6 Decision Criteria

**8.6.1. Dose Confirmatory Period:** during this phase of the trial the decision criteria comprises from the observation (or not) of DLTs and also the trough levels of circulating BTT1023 must have reached a minimum level of ≥3 µg/ml.

If ≤1 DLT is observed in a cohort of 6 patients receiving the same BTT1023 dose within the DLT monitoring period and trough levels of circulating BTT1023 are found to be <3 µg/ml (indicating that a therapeutic level **has not** been reached) either the dose will escalate or the trial will stop if the maximum test dose of BTT1023 has been reached.

If ≤1 DLT is observed in a cohort of 6 patients receiving the same BTT1023 dose within the DLT monitoring period and trough levels of circulating BTT1023 are found to be ≥3 µg/ml (indicating that a therapeutic level **has** been reached) this dose will be confirmed as the MED and the trial will continue into the Phase II stage of the trial.

If ≥2 DLTs are observed in a cohort of 6 patients receiving the same BTT1023 dose, within the DLT monitoring period, and the trough levels of circulating BTT1023 are found to be ≥3 µg/ml, then the dose will de-escalate. If the dose received is already at the lowest test dose, then the trial will stop.

If ≥2 DLTs are observed in a cohort of 6 patients receiving the same BTT1023 dose, within the DLT monitoring period, and the trough levels of circulating BTT1023 are found to be <3 µg/ml, then the trial will stop due to futility (i.e. the dose is producing toxic effects and is not achieving the required PK levels).

**8.6.2. Simon's Two-Stage Phase II:** this includes an interim assessment of the accumulating data. The first assessment (stage-1) takes place once 18 patients have been evaluated for the primary outcome (response). If 3 or more responses are observed in stage-1 then the trial will continue into stage-2. Recruitment will not be halted while stage-1 is assessed. If the stage-1 criterion is not met then the trial will cease. A further 19 patients will be recruited during stage-2.

## 8.7 Subgroup Analyses

Exploratory subgroup analyses may be performed based on stage of liver disease and/or prior treatments. For exploratory subgroup analyses, only the primary outcome measure (with central processing) will be assessed.

## 8.8 Sensitivity Analyses

Sensitivity analyses will also be performed in the per-protocol population, which is defined as those patients who completed the treatment as originally allocated with no dose modification or missing doses (i.e. patients that have received all 7 infusions as scheduled in the protocol at the MED dose). For sensitivity analyses, only the primary outcome measure (with central processing) will be assessed.

# 9. HEALTH ECONOMICS

No health economic analyses will be performed.

# 10. STATISTICAL SOFTWARE

Analyses will be conducted using R and associated packages.

## 11. STORAGE AND ARCHIVING

Snapshots of data used in interim and final analysis, programs and analyses will be stored and archived in relevantly labelled files in the following location: CRCTU Shared Areas (S:) / Stats / Shared / Trials Work / EDD / BUTEO /.

## 12. REFERENCES

[1] Richard Simon, Optimal Two-Stage Designs for Phase II Clinical Trials. *Controlled Clinical Trials 10:1-10 (1989)*

**Supplementary appendix 4: Treatment compliance and analysis populations**

| Phase Recruited Into | Included in Phase I DLT/Dose Evaluation | Included in Phase II Interim Analysis | Included in Phase II Final Analysis | Number of Timolumab Infusions | Comments |
|---|---|---|---|---|---|
| I | No | Yes | Yes | 1 | Withdrew (followed up as per SoC) and replaced for DLT evaluation, however evaluable for phase II interim as they had requisite initial and follow-up ALP |
| I | Yes | Yes | Yes | 7 | |
| I | Yes | Yes | Yes | 7 | |
| I | Yes | Yes | Yes | 7 | |
| I | Yes | Yes | Yes | 7 | |
| I | Yes | Yes | Yes | 7 | |
| I | Yes | Yes | Yes | 7 | |
| II | n/a | No | No | 0 | Patient found to be ineligible post registration – excluded from all analysis |
| II | n/a | Yes | Yes | 5 | Withdrew (followed up as per protocol) however as they had at least one timolumab infusion they are evaluable for phase II outcomes |
| II | n/a | Yes | Yes | 6 | Missed one treatment cycle, however evaluable for all phase II outcomes |
| II | n/a | Yes | Yes | 7 | |
| II | n/a | Yes | Yes | 7 | |
| II | n/a | Yes | Yes | 7 | |
| II | n/a | Yes | Yes | 7 | |
| II | n/a | Yes | Yes | 7 | |
| II | n/a | Yes | Yes | 7 | |
| II | n/a | Yes | Yes | 7 | |
| II | n/a | Yes | Yes | 7 | |

| Phase Recruited Into | Included in Phase I DLT/Dose Evaluation | Included in Phase II Interim Analysis | Included in Phase II Final Analysis | Number of Timolumab Infusions | Comments |
|---|---|---|---|---|---|
| II | n/a | Yes | Yes | 7 | Centrally reviewed ALP sample missing, therefore was included in all phase II analysis but classed as a non-responder (as per SAP) |
| The following patients were recruited during the interim assessment, before the Data Monitoring Committee had made their decision to discontinue the trial | | | | | |
| II | n/a | No | Yes | 7 | |
| II | n/a | No | Yes | 7 | |
| II | n/a | No | Yes | 7 | |
| II | n/a | No | Yes | 7 | |

Note: Information for each patient recruited is entered into a separate row within the table.

ALP, alkaline phosphatase; DLT, dose limiting toxicity; SoC, standard of care.

**Supplementary appendix 5: Confirmation of timolumab dose**

The confirmatory dose stage of the BUTEO trial determined a dose of timolumab (BTT1023) that provided an acceptable level of pharmacokinetic (PK) activity and was deemed to be safe, meeting the acceptable dose limiting toxicity (DLT) level. This was achieved by evaluating:

- The number of DLTs per dose level of timolumab (Table S4), and;
- PK data per dose level of timolumab (Figure S3).

*Table S5: All grade 3 or higher adverse events experienced by patients during the dose confirmation stage*

| Adverse Event Toxicity | Grade | Serious | Relatedness | DLT* |
|---|---|---|---|---|
| GGT increased | 4 | No | Unrelated | No |
| Infusion related reaction | 3 | No | Definitely related | - |
| Alkaline phosphatase increased | 3 | No | Unrelated | - |
| GGT increased | 3 | No | Unrelated | - |
| Infusion related reaction | 3 | Yes | Definitely related | - |
| Alkaline phosphatase increased | 3 | No | Unrelated | - |
| Hypotension | 3 | No | Unrelated | - |
| GGT increased | 3 | No | Unrelated | - |
| GGT increased | 3 | No | Unrelated | - |
| Cough | 3 | No | Unrelated | - |
| Pain | 3 | No | Unrelated | - |
| GGT increased | 3 | No | Unrelated | - |
| Alkaline phosphatase increased | 3 | No | Unrelated | - |
| Alkaline phosphatase increased | 3 | No | Unrelated | - |
| GGT increased | 3 | No | Unrelated | - |
| GGT increased | 3 | No | Unrelated | - |
| GGT increased | 3 | No | Unrelated | - |
| GGT increased | 3 | No | Unrelated | - |

Note: Events are grouped between dashed horizontal lines to indicate those that were experienced by the same patient.

* Defined as an adverse event that meets the criteria of grade 3 cytokine release syndrome or grade 4 or 5 for any criteria, as defined in the NCI-CTCAE v4.0 and considered to be at least possibly related to timolumab treatment

DLT, dose limiting toxicity; GGT, gamma-glutamyl transferase.

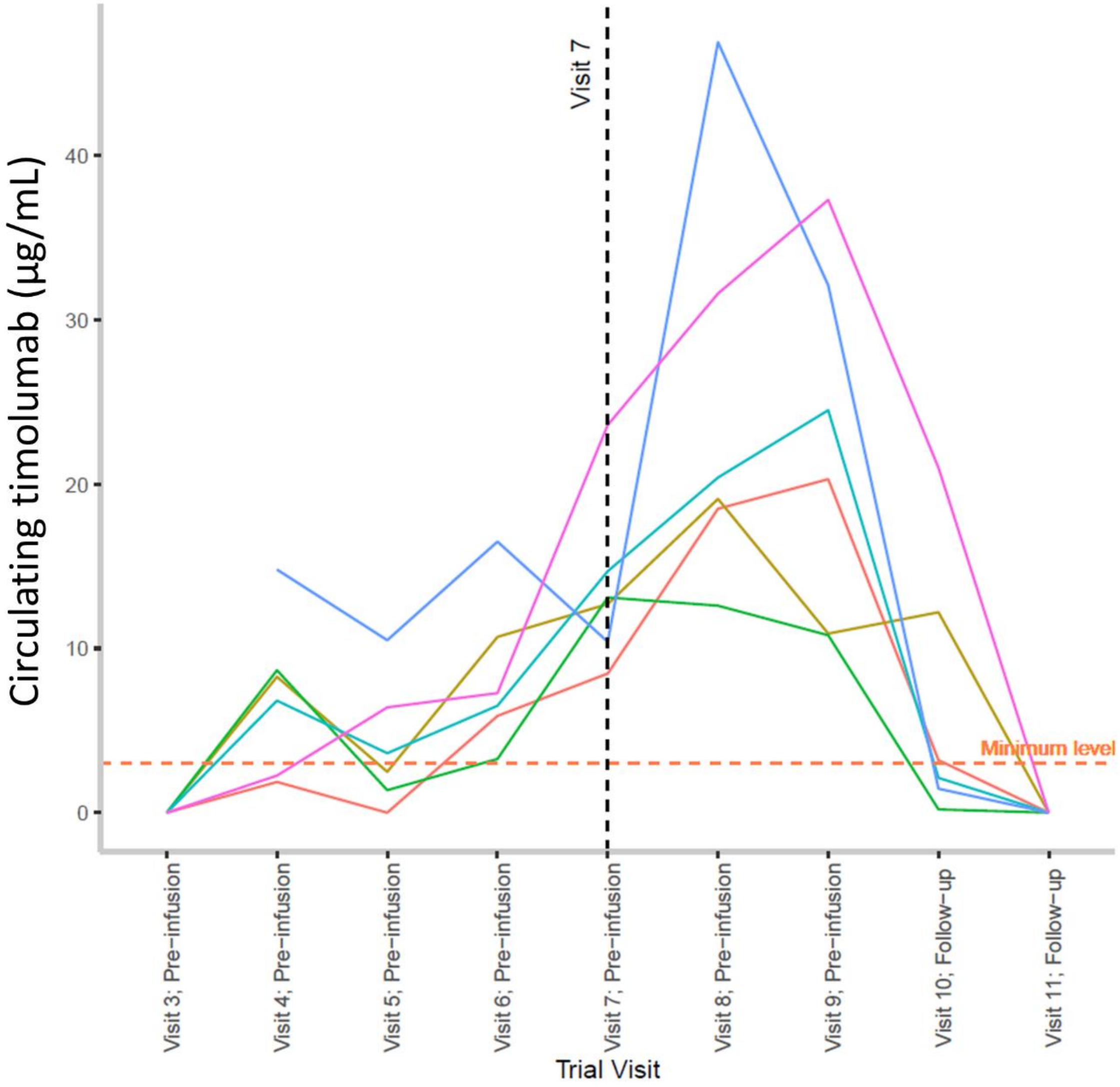


*Fig. S5: Repeated measures of circulating timolumab concentration in all patients*

The criterion for continuation to the Simon's two-stage expansion was for timolumab circulating trough concentration to reach 3 µg/mL 8 weeks from the first infusion as indicated by the horizonal dotted orange line. Week eight after the first infusion falls between visit 7 (day 50) and visit 8 (day 64), as indicated by the vertical dotted black line.

## Supplementary appendix 6: Secondary outcomes

*Table S6A: Serum liver tests and risk scores*

| | | Visit 3 | Visit 10 | Diff |
|---|---|---|---|---|
| AST | Median (IQR) | 71<br>(48.25, 102.25) | 73.5<br>(47.75, 94.5) | -10<br>(-15.8, 3.25) |
| | Range | (26, 250) | (23, 230) | (-337, 44) |
| ALT | Median (IQR) | 71<br>(60.5, 118.5) | 72<br>(53, 123) | -11<br>(25, 5) |
| | Range | (27, 255) | (29, 249) | (-36, 126) |
| ALP | Median (IQR) | 345<br>(228.25, 529.25) | 354.5<br>(273.25, 495.75) | -12<br>(-48.75, 23.25) |
| | Range | (180, 1069) | (142, 1407) | (-124, 452) |
| GGT | Median (IQR) | 460<br>(245, 984.75) | 342<br>(213, 924) | -8<br>(-39, 99) |
| | Range | (76, 1773) | (86, 2032) | (-138, 259) |
| Bilirubin (direct) | Median (IQR) | 8.5<br>(5, 24.5) | 8<br>(5, 17) | 0<br>(-2, 3) |
| | Range | (2, 35) | (3, 47) | (-11, 27) |
| Albumin | Median (IQR) | 42<br>(36.75, 43.75) | 42<br>(36.25, 43) | 0<br>(-2.75, 1.75) |
| | Range | (30, 49) | (29, 47) | (-4, 7) |
| INR | Median (IQR) | 1<br>(1, 1) | 1<br>(1, 1.08) | 0<br>(0,0) |
| | Range | (0.9, 1.2) | (0.8, 1.2) | (-0.1, 0.1) |
| Mayo PSC Risk score | Median (IQR) | 0.75<br>(0.25, 1.14) | 0.62<br>(0.28, 1.12) | -0.12<br>(-0.19, 0.08) |
| | Range | (0.03, 2.68) | (0.02, 2.84) | (-0.58, 0.4) |
| MELD | Median (IQR) | 7<br>(6, 10) | 7<br>(6, 8) | 0<br>(-1, 0) |
| | Range | (6, 12) | (5, 11) | (-3, 5) |

*Abbreviations*: AST: Aspartate aminotransferase. ALT: Alanine aminotransferase. ALP: Alkaline Phosphatase. GGT: Gamma Glutamyl Transferase. INR: International Normalised Ratio. MELD: Model for End-stage Liver Disease.

*Table S6B: LiverMultiscan LIF score*

| | | Visit 1 | Visit 11 | Difference |
|---|---|---|---|---|
| ROI LIF | Median (IQR) | 1.14 (0.56, 1.74) | 1.26 (0.67, 2.55) | 0.31 (-0.04, 0.75) |
| | Range | (0, 3.42) | (0.47, 4) | (-1.81, 3.55) |

Abbreviations: ROI: Region of Interest. LIF: Liver Inflammation and Fibrosis

**Supplementary appendix 7: Circulating populations of inflammatory cells**

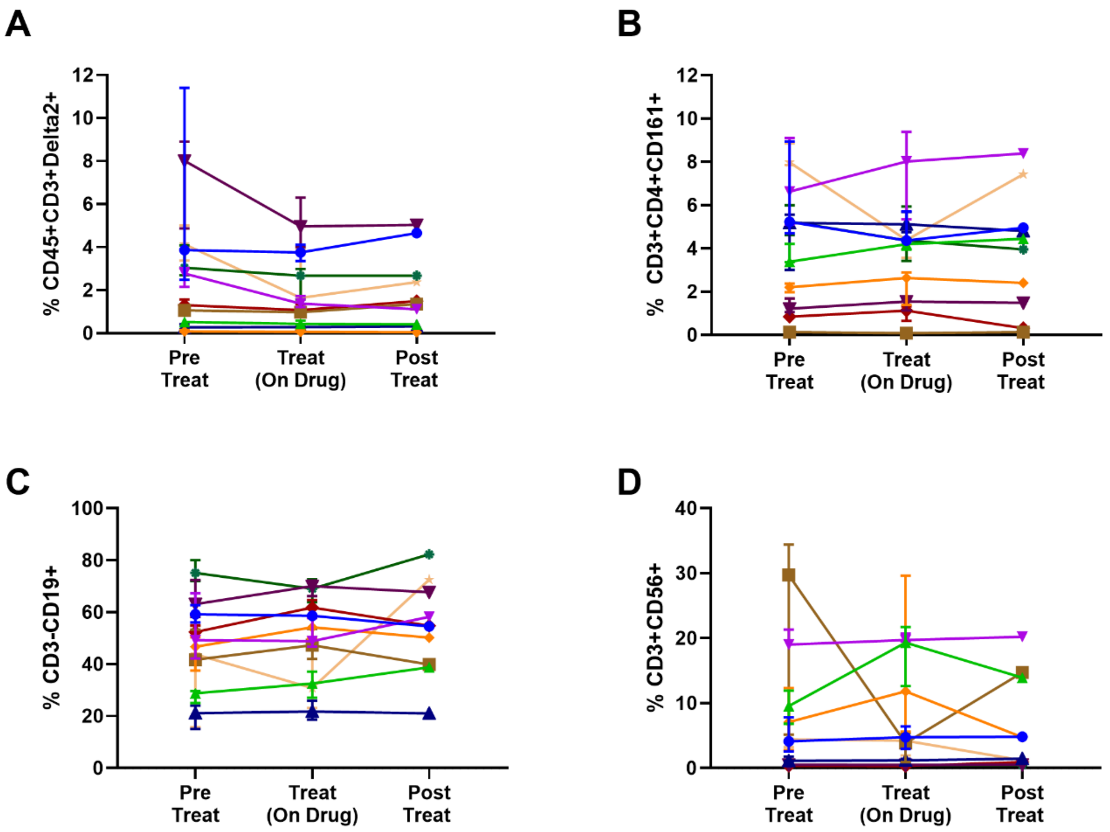


*Fig. S7: Proportions of circulating inflammatory cells that may also contribute to the pathogenesis of PSC*

Cryopreserved peripheral blood mononuclear cells were thawed and immunophenotyped using flow cytometry for (A) gamma-delta T-cells (CD3+CD45+delta2 TCR+), (B) Th17 cells (CD3+CD4+CD161+), (C) B cells (CD3-CD19+) and (D) NKT cells (CD3+CD56+). Aggregated data collected during pre-treatment (screening visits 1 and 2, and pre-treatment (visits 1-3: screening visits 1 and 2, pre-first infusion visit 3 (day 1)), treatment (infusion visits 4-9: days 8, 22, 36, 50, 64, and 78) and post-treatment (follow-up visits 10-11: day 99 and 120) groups. There were no significant differences between groups (Kruskal-Wallis test with Dunn's multiple comparison test). Median +/- interquartile range.

**Supplementary appendix 8: Markers of fibrogenesis and fibrolysis**

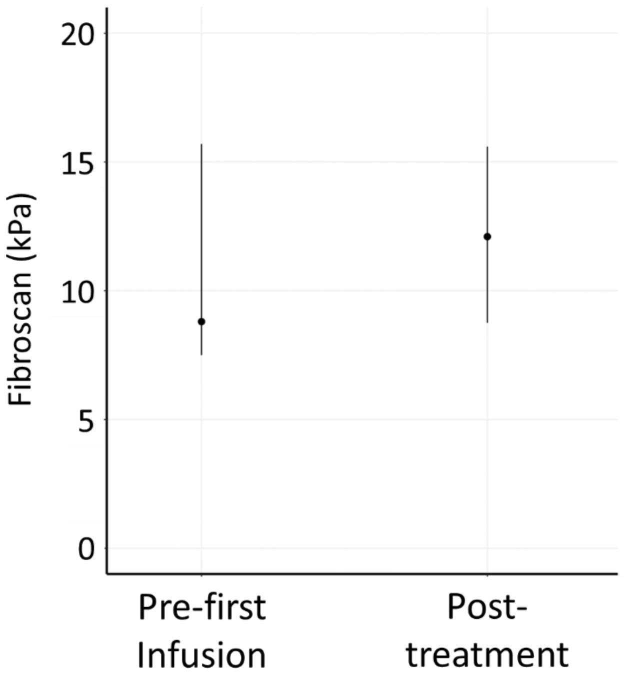


*Fig. S8A: Liver fibrosis*

Liver fibrosis as determined by Fibroscan pre-first infusion on visit 3, day 1, and post-treatment on visit 10, day 99. Medians with interquartile ranges are shown. kPa, kilopascal.

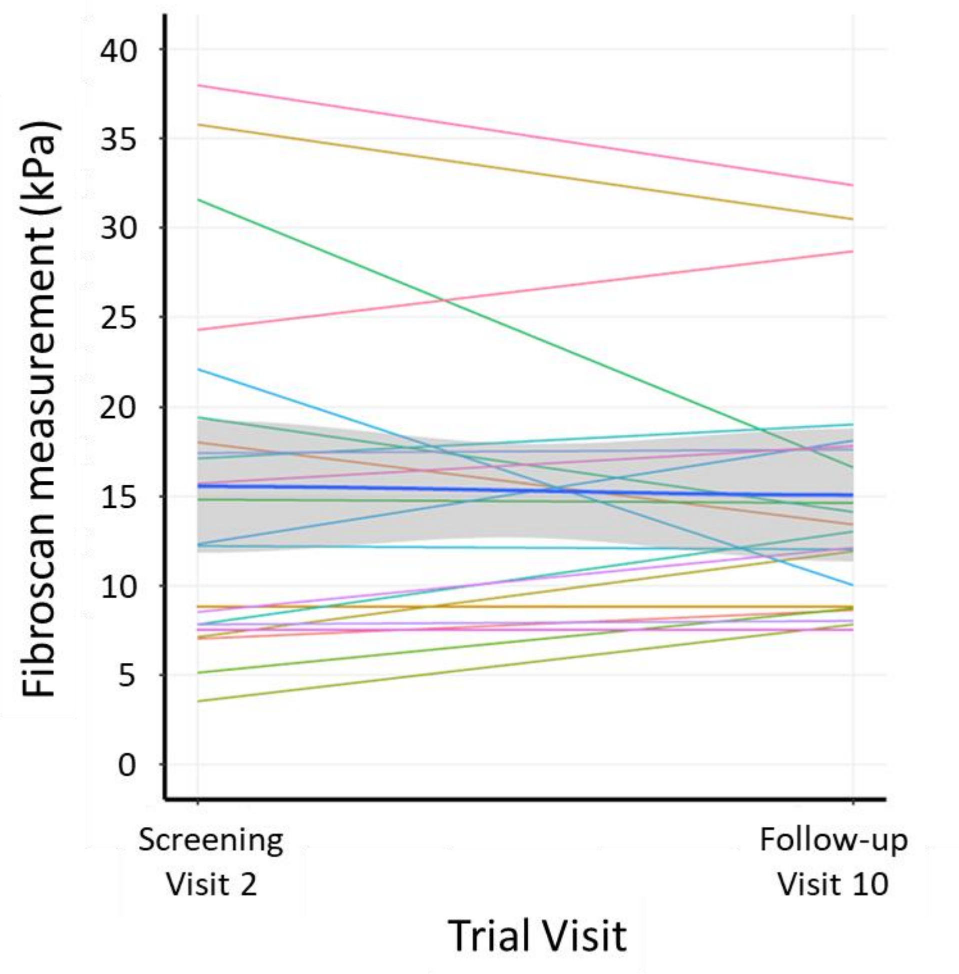


*Fig. S8B: Repeated measures plot of Fibroscan measurements for all evaluable patients in the mITT population.*

A loess smoother trend line is shown in dark blue (thicker line) with uncertainty depicted by the shaded grey region. Since Fibroscan was only completed at screening visit 2 and follow-up visit 10, any repeated measures analysis would be inappropriate, and has, therefore, not been performed.

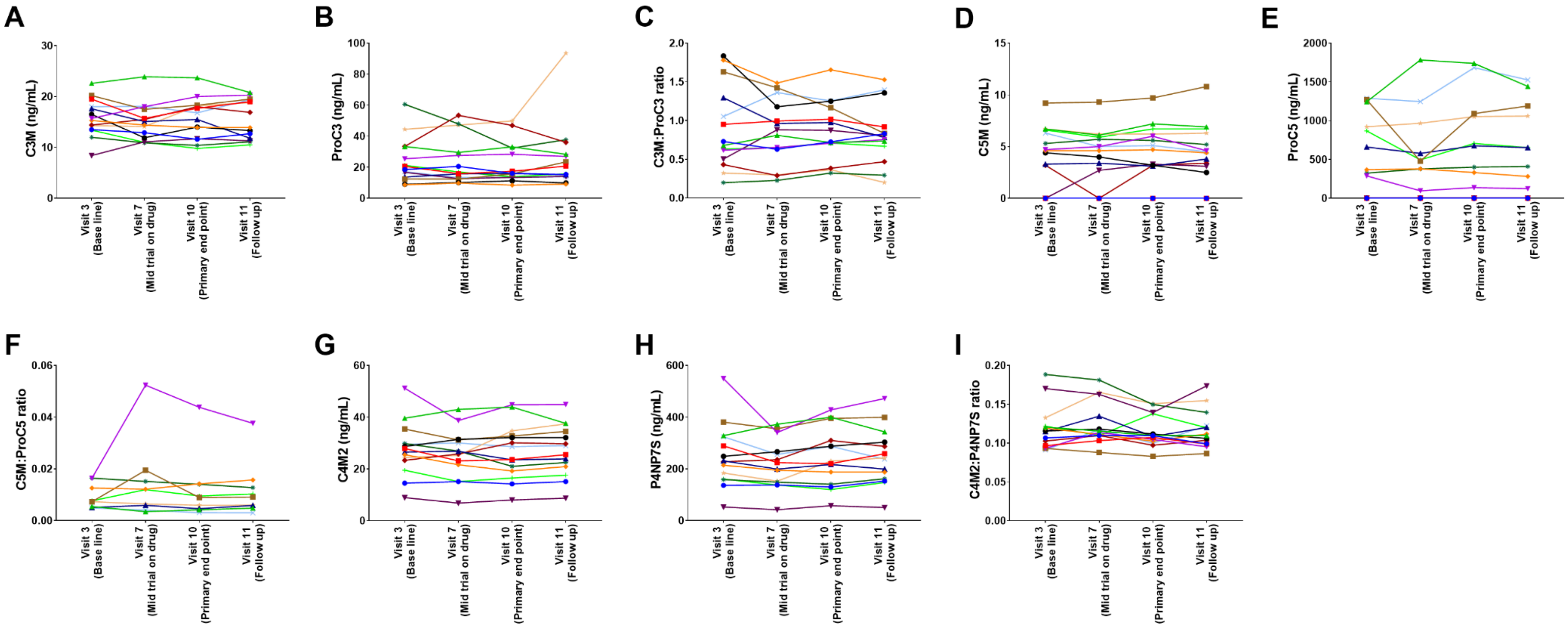


*Fig. S8C: Concentration of circulating markers of ECM synthesis and turnover*

Concentrations of circulating (A) C3M, (B) ProC3, (D) C5M, (E) ProC5, (G) C4M2 and (H) P4NP7S (markers of ECM turnover) were measured by Nordic Bioscience at baseline (visit 3), mid-treatment (visit 7), at the time of primary endpoint (visit 10) and at last follow up (visit 11). There were no significant differences observed for either the raw data or ratio of ECM synthesis and breakdown products ((C) C3M:ProC3, (F) C5M:ProC5, (I) C4M2:P4NP7S; Kruskal-Wallis test with Dunn’s multiple comparison test). Median +/- interquartile range. Note that samples with ProC5 concentrations below the limit of detection were assigned a value of zero and were subsequently excluded from the calculation of C5M:ProC5 ratio.

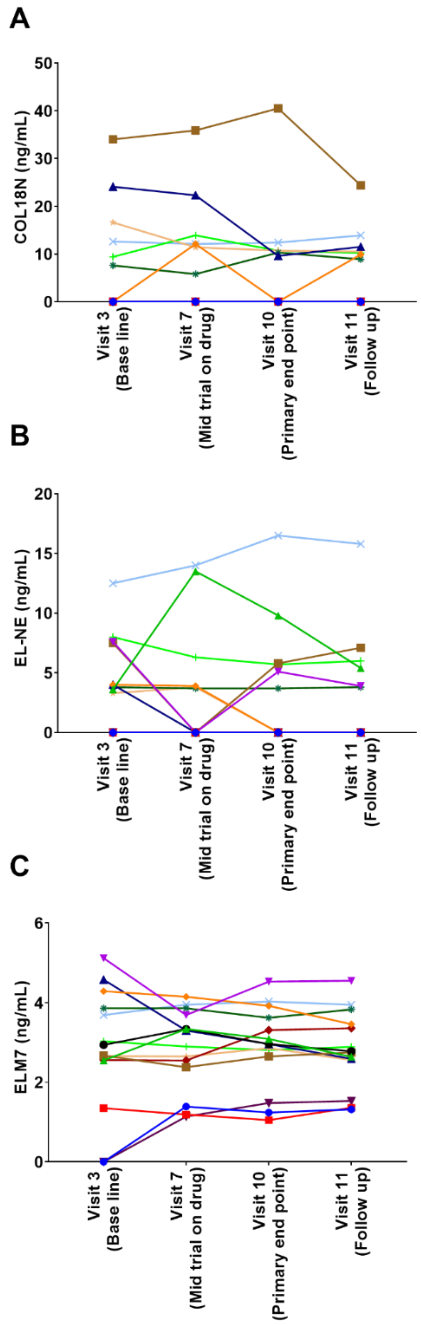


*Fig. S8D: Concentration of exploratory circulating markers of fibrotic tissue remodelling*

Concentrations of circulating (A) COL18N, (B) EL-NE and (C) ELM7 (markers of ECM turnover) were measured by Nordic Bioscience at baseline (visit 3), mid-treatment (visit 7), at the time of primary endpoint (visit 10) and at last follow up (visit 11). There were no significant differences observed for the raw data (Kruskal-Wallis test with Dunn's multiple comparison test). Median +/- interquartile range. Note that samples with concentrations below the limit of detection were assigned a value of zero.
